# Integrated ‘Shield‐Spear’ Biological Patch for Fibrosis‐Free Bladder Reconstruction

**DOI:** 10.1002/advs.202503975

**Published:** 2025-08-13

**Authors:** Xiaoqi Wu, Huitong Ruan, Xiaolin Zhang, Weihan Zheng, Muhetaierjiang Mutailifu, Ling Wang, Liu Yu, Ruijun Peng, Rui Zhao, Zihan Wang, Jie Xu, Shaochuan Li, Yaobin Wu, Wenguo Cui, Mujun Lu

**Affiliations:** ^1^ Department of Urology and Andrology Ren Ji Hospital School of Medicine Shanghai Jiao Tong University Shanghai 200001 China; ^2^ Department of Orthopaedics Shanghai Key Laboratory for Prevention and Treatment of Bone and Joint Diseases Shanghai Institute of Traumatology and Orthopaedics Ruijin Hospital Shanghai Jiao Tong University School of Medicine 197 Ruijin 2nd Road Shanghai 200025 China; ^3^ Guangdong Engineering Research Center for Translation of Medical 3D Printing Application Guangdong Provincial Key Laboratory of Medical Biomechanics Department of Human Anatomy School of Basic Medical Sciences Southern Medical University Guangzhou 510515 China; ^4^ South China Agricultural University Guangzhou 510642 China

**Keywords:** aptamer, bladder injury repair, bladder tissue engineering, extracellular vesicles, nerve repair, wet‐adhesive hydrogel

## Abstract

Bladder reconstruction without fibrosis remains a global challenge. Current bladder defect treatments primarily focus on repair at the level of vascularization, failing to balance healing and excessive collagen deposition, and neglecting the exacerbation of fibrosis due to neural dysregulation. In this study, an integrated ‘shield‐spear’ patch composed of an anionic ‘shield’ hydrogel (HAD) and neuro‐targeted ‘spear’ engineered extracellular vesicles (S100Aptamer‐EVs) using Schiff base chemistry and Michael addition reactions is engineered. This novel approach represents the first attempt to synergistically balance the contradiction between fibrosis and wound healing through the ‘shield‐spear’ strategy, achieving extensive bladder reconstruction without fibrosis. In a large animal model of beagles, the outer layer of the ‘shield‐spear’ patch acts as an anionic shield, neutralizing scavenger receptors and selectively capturing GATA6^+^ peritoneal macrophages to suppress collagen overexpression. The inner layer enables the unidirectional release of S100AptEVs, targeting the activation of Schwann cells to express the brain‐derived neurotrophic factor neuroprotective factor, downregulating the TGFβ/Smad fibrosis pathway, thereby collaboratively inhibiting neurogenic fibrosis and activating Cadherin signaling to promote wound healing. The integrated ‘shield‐spear’ patch facilitated surgical manipulation in large animals and provides a promising approach for tissue‐engineered bladder reconstruction without fibrosis.

## Graphical abstract

Fibrosis‐free bladder reconstruction remains challenging. This study pioneers an integrated “shield‐spear” patch: The outer anionic hydrogel layer captures GATA6+ macrophages to suppress collagen overexpression, while the inner S100 aptamer‐engineered EVs target Schwann cells to downregulate the TGFβ/Smad pathway—inhibiting fibrosis with enhanced wound healing. Validated in beagles, this dual‐functional patch synergistically offers a novel strategy for scarless bladder reconstruction.

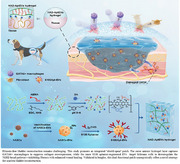

## 1 Introduction

Treatment of clinical conditions such as spinal cord injury, congenital malformations, and malignant tumors can mandate bladder wall resection.^[^ ^1^ ^]^ Treatment of bladder defects can lead to bladder wall thickening, reduced compliance, and formation of irreversible fibrotic lesions, ultimately leading to the loss of bladder contraction. Prevention of postoperative bladder fibrosis after reconstruction remains a global challenge.^[^ ^2^ ^]^ The current gold standard for the treatment of bladder defects is substitution with gastrointestinal tissues. However, this procedure is associated with bladder fibrosis.^[^ ^3^ ^]^ With the development of tissue engineering and regenerative medicine, flexible patches have been employed as alternative measures,^[^ ^3^, ^4^ ^]^ as evidenced by their feasibility in promoting bladder vascularization repair.^[^ ^4a^ ^]^ However, tissue‐engineered bladders are still associated with bladder fibrosis caused by the dual impact of collagen remodeling imbalance and neural dysregulation.^[^ ^3b^ ^]^ Current research is focused on a single aspect, either collagen remodeling or neural repair, failing to demonstrate fibrosis‐free reconstruction.^[^ ^5^ ^]^ Therefore, an integrated patch that is both suitable for surgical use and capable of dual regulation is required for fibrosis‐free bladder reconstruction.^[^ ^3b^ ^]^

Preventing abnormal deposition of collagen at the defect site is the key to fibrosis‐free bladder reconstruction.^[^ ^3a,b^ ^]^ Recent studies indicate that after abdominal surgery, free GATA6^+^ macrophages in the peritoneal cavity rapidly accumulate at the wound site^[^ ^6^ ^]^ through regulation by genes related to inflammation and extracellular matrix (ECM).^[^ ^7^ ^]^ This results in an imbalance in collagen remodeling in the bladder, leading to bladder wall thickening, reduced compliance, and formation of a fibrotic bladder with small capacity and high pressure. This is one of the extravesical factors influencing fibrosis healing after bladder injury.^[^ ^8^ ^]^ Additionally, local nerve injury following bladder defects can lead to neurogenic fibrosis and exacerbate functional impairment.^[^ ^9^ ^]^ Peripheral nerves are primarily composed of axons and Schwann cells (SCs). Following bladder nerve damage, SCs play a crucial role in the reconstruction of neural myelin sheaths and axonal regeneration.^[^ ^10^ ^]^ Brain‐derived neurotrophic factor (BDNF), they reduce the expression of Type I collagen (COL1), thereby inhibiting the occurrence of neurofibrosis.^[^ ^11^ ^]^ In addition, the BDNF analog 7,8‐Dihydroxyflavone has also been shown to alleviate myocardial fibrosis. Therefore, SCs and the BDNF they secrete play a significant role in improving bladder fibrosis and may be key factors in ameliorating neurogenic bladder fibrosis.^[^ ^11b^ ^]^ Therefore, the loss of local SCs after bladder injury is one of the “intravesical” factors contributing to fibrosis healing. Currently, most reported materials only achieve vascularization repair of the bladder, overlooking the extensive deposition of ECM during the defect repair process and the role of nerve damage in fibrosis;^[^ ^3^, ^4^ ^]^ Additionally, multi‐layered physically superimposed multifunctional biological scaffolds, due to the inconsistency in the physicochemical properties and biocompatibility of each layer, are not conducive to surgical management and clinical application.^[^ ^4^, ^12^ ^]^ Therefore, it is necessary to design an integrated anti‐bladder fibrosis strategy that can simultaneously balance the excessive recruitment of GATA6^+^ macrophages during wound healing while enhancing the neuroprotective and fibrosis‐regulating roles of SCs. This strategy should synergistically improve fibrosis healing in the bladder from both “intravesical” and “extravesical” perspectives.

In this study, a ‘shield‐spear’ bladder patch is proposed for fibrosis‐free bladder repair. Common clinical practices involve the use of tissue adhesives to seal traumatic defects; however, their weak adhesive properties often lead to indiscriminate tissue adhesions, which are not conducive to anti‐fibrotic repair.^[^ ^13^ ^]^ Autologous tissues or biomimetic patches, such as PCL and hyaluronic acid (HA), require suture fixation. However, suturing causes secondary damage to the already fragile bladder smooth muscle and serosa layers, leading to inflammatory infiltration and exacerbating fibrotic thickening.^[^ ^4^, ^14^ ^]^ Polyanionic materials, such as heparin, polyguanosine acid, and HA, can attract GATA6^+^ macrophages with positive charges on their surfaces, disrupting the mutual recognition between GATA6^+^ macrophages and effectively preventing their aggregation at the wound site.^[^ ^6a^ ^]^ Therefore, it is necessary to develop a wet adhesive bladder patch that can serve as a polyanion shield to disrupt GATA6^+^ aggregation and achieve anti‐fibrotic reconstruction. Moreover, the activation of SCs can improve peripheral nerve repair and inhibit neurogenic bladder fibrosis.^[^ ^11^, ^15^ ^]^ Extracellular vesicles (EVs) derived from adipose‐derived stem cells (ASCs) can activate SCs through paracrine effects, upregulating BDNF expression to inhibit neuropathy.^[^ ^11^, ^16^ ^]^ Previous studies used composite ASCs‐EVs incorporated into alginate/chitosan scaffolds to induce SC activation.^[^ ^17^ ^]^ However, traditional scaffolds often release EVs diffusely into the surrounding tissue, failing to concentrate at the wound site where SCs are located.^[^ ^18^ ^]^ Additionally, natural EVs cannot specifically target SCs, resulting in inefficient nerve repair at the wound site.^[^ ^19^ ^]^ Currently, no materials are capable of simultaneously intervening in GATA6^+^ aggregation and improving neural protection against fibrosis.^[^ ^3a,b^ ^]^ Therefore, we designed an integrated ‘shield‐spear’ Janus bladder patch, with the ‘shield’ side capable of trapping and disrupting GATA6^+^ macrophage aggregation, thereby inhibiting excessive ECM deposition. Meanwhile, the ‘spear’ side unidirectionally releases engineered EVs to the defect site, targeting SCs and efficiently inducing their secretion of BDNF. This dual functionality of the integrated ‘shield‐spear’ effectively suppressed fibrosis progression (Figure S1, Supporting Information).

**Figure 1:**
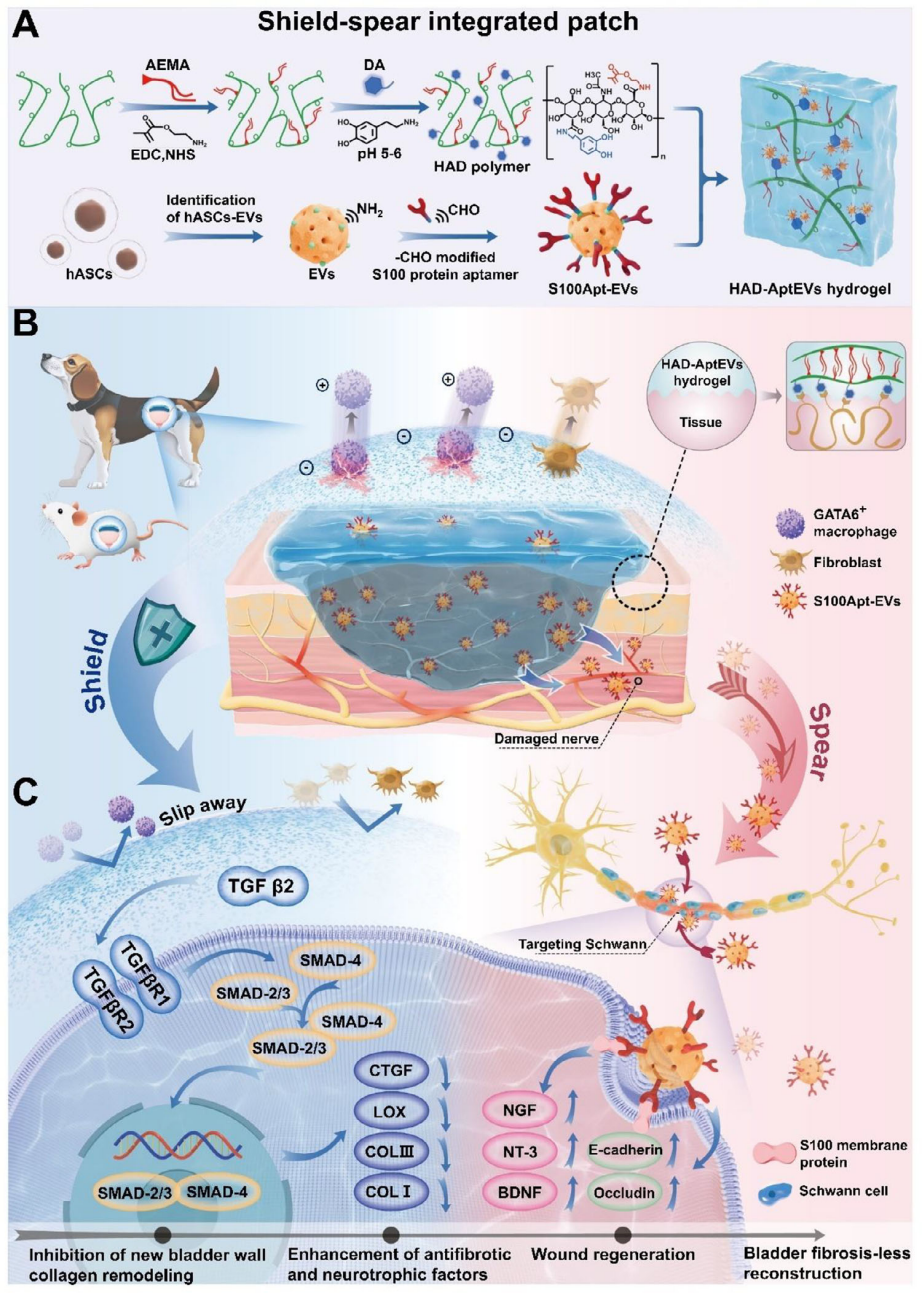
The design concept of ‘shield‐spear’ integrated biological patch. A) The schematic diagram of synthesis of the integrated ‘shield‐spear’ Janus HAD‐AptEV patch. B) The integrated ‘shield‐spear’ patch could efficiently adhere to large‐area defects without suturing in rats and beagle dogs to achieve fibrosis‐free bladder reconstruction. C) The molecular mechanism of the integrated ‘shield‐spear’ patch in inhibiting fibrosis, activating neuroprotection, and promoting wound healing.

Herein, a ‘shield‐spear’ integrated bladder patch (HAD‐AptEV hydrogel) was developed for fibrosis‐free bladder reconstruction, aimed at capturing GATA6^+^ macrophages and targeting the activation of SCs. We utilized an active esterification reaction to develop an HA‐based polyanionic ‘shield’ hydrogel (HAD); employing Schiff base chemistry, we grafted aldehyde‐modified S100 protein aptamer sequences (S100Apt‐EVs) onto the surface of ASCs‐EVs as the ‘spear’ targeting SCs,^[^ ^20^ ^]^ enhancing the uptake efficiency of EVs by SCs.^[^ ^21^ ^]^ Finally, the integrated HAD‐AptEV hydrogel was developed via a Michael‐type reaction (**Figure** **1**A). In vitro experiments demonstrated that the ‘shield’ side of the patch effectively captured GATA6^+^ macrophages, preventing the aggregation of fibroblasts at the wound site; the ‘spear’ side firmly adhered to the bladder defect and unidirectionally released S100Apt‐EVs toward the SCs at the injury site, promoting SCs to express BDNF, synergistically inhibiting fibrosis (Figure 1B). The Beagle large animal model demonstrated that the integrated ‘shield‐spear’ patch could rapidly respond to surgical management, effectively sealing bladder defects in a complex in vivo environment, maintaining bladder contractions and expansions, and preventing postoperative fibrous adhesion and stone formation. Molecular mechanism exploration revealed that the ‘shield‐spear’ patch targets and activates SCs through S100Apt‐EVs to secrete BDNF, regulating the microenvironment at the site of bladder injury, inhibiting the TGF‐β/Smad signaling pathway, downregulating LOX family proteins, and COL1 to inhibit fibrosis; additionally, the introduction of S100Apt‐EVs activated the Cadherin signalinpathway, promoting cell migration and re‐epithelialization for wound healing. Therefore, the ‘shield‐spear’ patch can balance the contradiction between wound healing and anti‐fibrosis, with nerve repair, achieving fibrosis‐free bladder reconstruction synergistically on both fronts (Figure 1C). In conclusion, the integrated ‘shield‐spear’ patch strategy is a novel tissue engineering strategy for the fibrosis‐free repair of large tissue defects after surgery.

## 2 Results and Discussion

### 2.1 Synthesis and Characterization of the ‘shield‐spear’ Hydrogel

**Figure 2:**
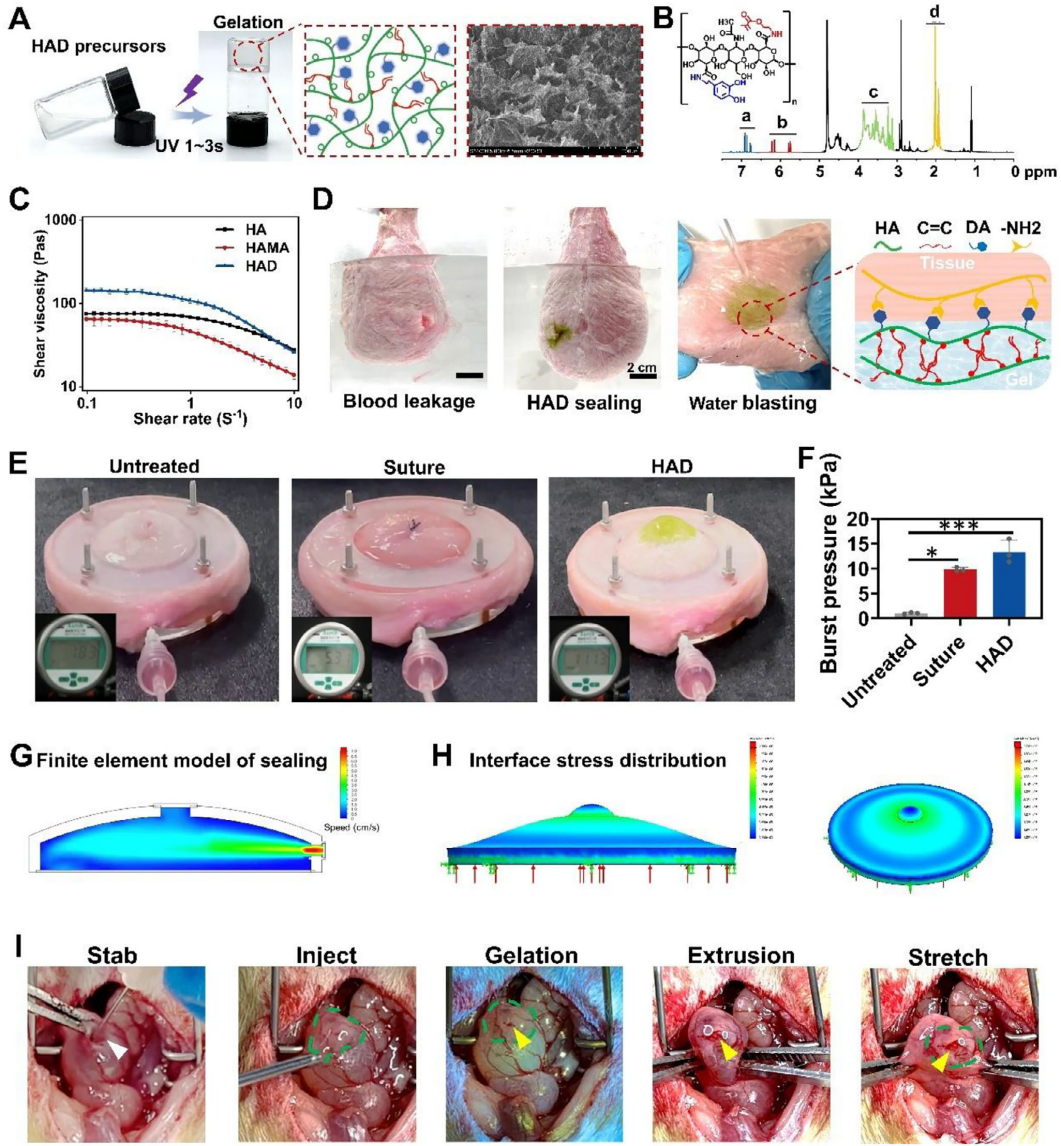
Characterization of HAD hydrogel. A) Photo of the HAD hydrogel rapidly solidifying under UV light. B) Proton nuclear magnetic resonance spectra of HAD. a labeled protons in the catechol ring of dopamine (δ = 6.5–7.2 ppm); b labeled C═C chain of 2‐aminoethyl methacrylate hydrochloride (AEMA) (δ = 5.68 and 6.13 ppm); c labeled protons in the ring structures of HA (δ = 4.0–3.0 ppm); d labeled C(═O) CH3 chain in HA (δ = 2.1 ppm). C) Rheological viscosity test of HAD, HA polymer grafted with AEMA, and HA under different shear rates. D) The ICG‐labeled HAD successfully adhered underwater, sealing a 2 cm diameter defect in the porcine bladder, preventing blood exposure. The HAD adhered strongly to the bladder and could withstand the pressure of high‐speed running water. E) Bursting pressure test of ex vivo porcine bladder with a 1 cm diameter defect sealed by surgical sutures and HAD hydrogels, respectively. Untreated bladders were used as controls. F) Maximum bursting pressure of bladder defect sealed with sutures and HAD. Untreated bladders were used as controls (All data are presented as mean ± SD from at least three independent experimental samples. *p < 0.05; ***p < 0.001). G) Finite element analysis: internal stress distribution map when physiological saline was injected into the bladder at 2 mL min^−1^. H) Stress–strain distribution map of HAD under maximum bladder pressure. I) Representative image of a 5‐millimeter hole created on the rat bladder and sealed with the HAD hydrogel: firm adhesion of the HAD prevented urinary leakage, bleeding, and withstood bladder compression and stretching.

The HAD precursor was synthesized by grafting 3,4‐dihydroxyphenylalanine (DA) and 2‐aminoethyl methacrylate (AEMA) onto the HA chain, enabling its adhesion and photocrosslinking capabilities (Figures 1A and **2**A). The selection of HA as the core chain was supported by its extensive application as a polyanionic ligand in clinical settings, and specifically because of its anti‐adhesive properties.^[^ ^6^, ^22^ ^]^ Proton nuclear magnetic resonance (NMR) and Fourier‐transform infrared spectroscopy analyses indicated the successful grafting of AEMA and DA groups onto the HA polymer chain. The peaks from 6.6 to 7.2 ppm in the HAD 1H NMR spectra indicated the protons in the catechol ring of the DA group. The peaks at 5.67 and 6.12 ppm were assigned to the C═C bond protons of the AEMA group (Figure 2B; Figure S2A,B, Supporting Information). The degree of substitution of AEMA in the HAD is 55.5%, and the degree of substitution of DA is 16%, by integrating the 1H NMR spectra (Figure S2C,D, Supporting Information), using calculation methods from a previous study.^[^ ^23^ ^]^ Subsequent to photocrosslinking of the aqueous HAD precursor solution in the presence of the LAP photoinitiator under ultraviolet (UV) irradiation (365 nm) for 3 s, the HAD hydrogel was formed with a porous microarchitecture (Figure 2A). Rheological assessments of the HAD precursor subjected to distinct shear rates substantiated its shear‐thinning behavior, which was analogous to that of HA polymer grafted with AEMA (HAMA) and the HA polymer solution (Figure 2C). The viscosity of the HAD precursor exceeded that of the HA and HAMA solutions. The hydroxyl groups within the DA moiety preserved the hydrogen‐bond interactions, culminating in viscosity levels similar to those of HA. Consequently, the HAD precursor exhibited injectable properties owing to its intrinsic shear‐thinning behavior. The photocrosslinked HAD hydrogel adhered to the bladder surface, and running water did not rinse it off for minutes (Figure 2D). Despite being exposed to stretching, bending, and twisting deformations, HAD maintained consistent adherence to the bladder surface (Figure S3, Supporting Information). UV‐vis spectroscopy analysis indicated that after 14 days of oxidation, the phenyl ring structure of the catechol groups in the HAD precursors underwent transformation to quinone rings. This observation is of utmost significance because it shows that the catechol groups in HAD undergo slow oxidation and maintain sustained adhesion.^[^ ^22^ ^]^ Conversely, in HAMA, the absence of catechol amine groups precludes the formation of quinone ring structures, thereby negating any oxidative adhesion processes (Figure S4, Supporting Information). Frequency sweep analysis indicated that an increased concentration of DA in the hydrogels led to a diminished storage modulus. Additionally, the storage modulus (G′) consistently exceeded the loss modulus (G′′), and no crossing point between G′ and G′′ was observed across the frequency range of 0.1 to 10 Hz or the strain range of 1% to 100% (Figure S5, Supporting Information). Additionally, the HAD hydrogels effectively sealed leakage in the bladder underwater. In a bladder with a defect diameter of 2 cm, internal blood rapidly leaked into the surrounding fluid environment. However, the sealing action of the HAD hydrogel successfully prevented blood leakage from the bladder defect, resulting in the surrounding fluid returning to a clear and transparent state. The HAD hydrogel exhibited good underwater adhesive properties owing to the strong interaction between its internal catechol groups and natural nucleophilic agents in the protein on the surface of the bladder tissue,^[^ ^24^ ^]^ ensuring that HAD maintains its integrity during friction with abdominal organs (Figure 2D).

To evaluate the adhesive properties of the HAD hydrogels quantitatively, we used a porcine bladder to perform fluid bursting pressure tests (Figure 2E; Figure S6, Supporting Information). With a hole of 1 cm diameter, the untreated porcine bladder tissues withstood a bursting pressure ≈0.8 ± 0.12 kPa (Figure 2e,f). Running water bursting out of the hole was detected (Movie S1, Supporting Information). Stabbed porcine bladder tissue treated with surgical sutures could endure a maximum bursting pressure of 9.8 ± 0.12 kPa (Figure 2E,F; Movie S1, Supporting Information). In contrast, after applying HAD hydrogels, the stabbed porcine bladder endured a maximum bursting pressure ≈15.8 ± 0.12 kPa, which was 15 times and 1.5 times that of the untreated and suture groups, respectively (Figure 2E,F; Movie S1, Supporting Information). Moreover, robust adhesion and fluid‐tight sealing were achieved within 1–3 s via photocrosslinking at the bladder defect by the ultrafast gelation of the HAD hydrogel, which was significantly shorter than the suturing duration. The stress‐strain distribution map of the bladder at the moment of bursting under fluid compression is shown in Figure 2g,h (Figure S7, Supporting Information). Based on finite element model calculations, filling the bladder at a flow rate of 2 mL min^−1^ (resulting in an internal pressure of 15 kPa) (Figure 2G) revealed stress concentration ranging from 5.03 × 10^5 ^to 1.33 × 10^6^ Pa at the interface between the HAD hydrogel and the defect (Figure 2H). At this moment, the interface between the hydrogel and the defect exhibited a deformation toward the surrounding area with a strain value of ε = 4.97 × 10^4^, while the top of the HAD gel exhibited a larger deformation with a strain value of ε = 5 × 10^6^ (Figure S7A, Supporting Information). By applying the same flow rate and internal bladder pressure to the untreated bladder defect model, the untreated bladder defect area generated uniformly distributed stress of 1.5 × 10^6^ to 2 × 10^6^ Pa (Figure S7B, Supporting Information). Furthermore, it exhibited a more pronounced expansion strain toward the surrounding area, with a strain magnitude of ε = 8.5 × 10^4^ (Figure S7B, Supporting Information). Finite element models demonstrated that the adhesive strength of HAD can withstand the deformation and tension caused by bladder expansion. Upon puncturing the rat bladder filled with urine, HAD solidified at the defect site within 3 s, sealing the defect (Figure 2I). The HAD effectively maintained bladder filling under compression and stretching, thereby achieving urinary fistula closure and hemostasis. These results demonstrate that the HAD gel patches possess appropriate mechanical strength and tissue adhesion, offering a rapid, convenient, and effective sealing solution for bladder defects.

### 2.2 Shield Effect by Inhibiting the Aggregation of GATA6^+^ Macrophages

**Figure 3:**
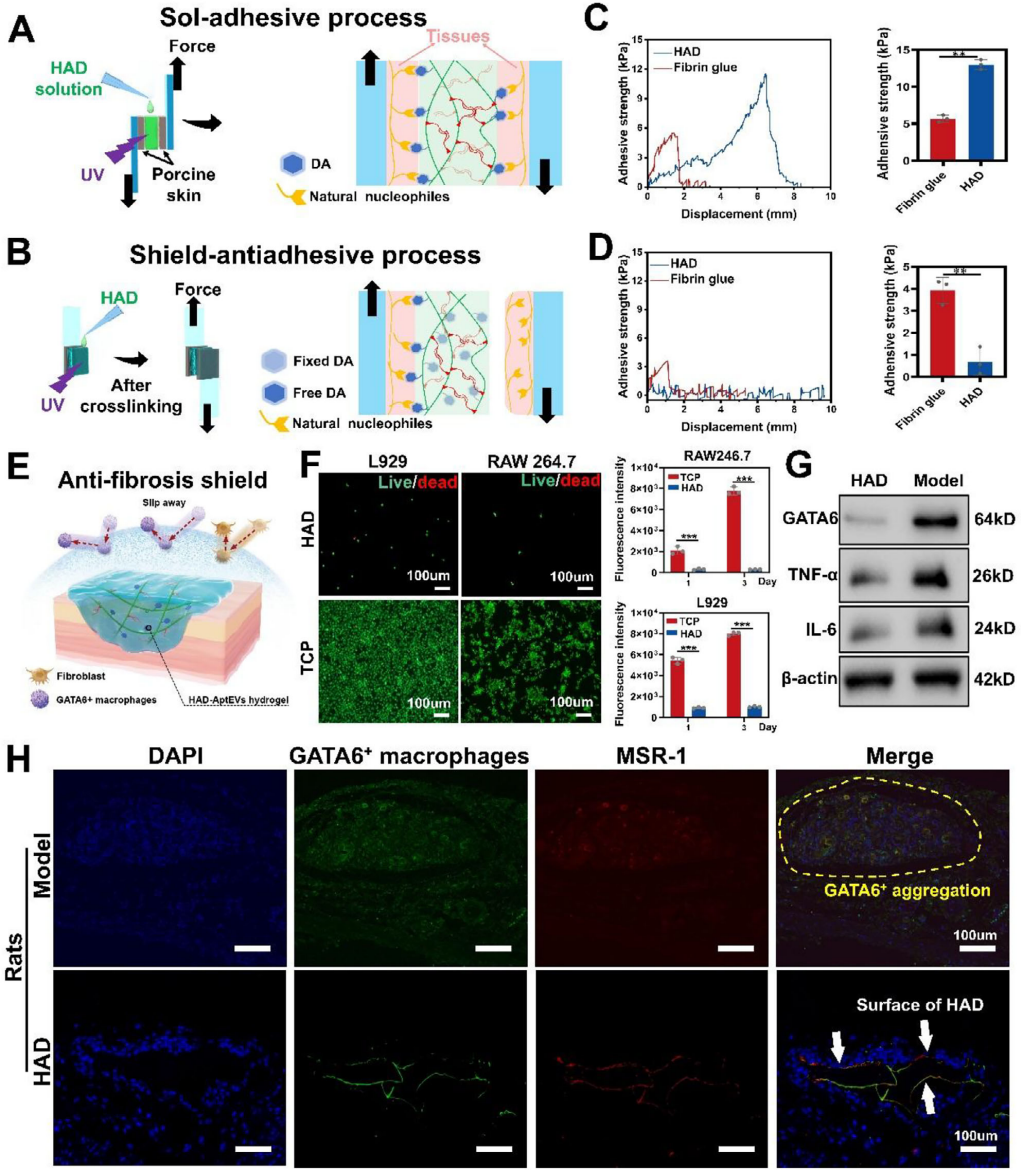
The “Shield‐spear” patch achieves the shield effect by resisting GATA6^+^ macrophage adhesion. A,B) Schematics showing the lap‐shear test procedure following the a) “sol‐adhesive” and b) “gel‐non‐adhesive” processes. Adhesive strength between the hydrogel and porcine skin following the C) “sol‐adhesive” and D) “gel‐non‐adhesive” processes. E) A schematic showing that the surface of ‘shield‐spear’ HAD hydrogel can inhibit the aggregation of macrophages and fibroblasts after crosslinking. F) L929 and RAW264.7 cell adhesion on HAD hydrogel and TCP (control). Culture system:5% CO2 concentration, 10% fetal bovine serum, and DMEM basal medium. The control group used TCP. G) Protein expressions of GATA6^+^, TNF‐α, and IL‐6 in the HAD and model group. The protein samples were obtained from SD rat bladder tissue 4 weeks after surgery. H) Tissue sections, GATA6^+^ macrophages, and positively charged type I scavenger receptor (MSR‐1) were stained with DAPI (blue), Alexa Fluor 488 (green), and Alexa Fluor 597 (red), respectively. Yellow dashed line represents GATA6^+^ macrophage aggregation; white arrows represent the surface of HAD hydrogels. The tissue samples were obtained from SD rat bladder tissue 4 weeks after surgery. Scale bars = 100 µm. All data are presented as mean ± SD from at least three independent experimental samples. **p < 0.01 and ***p < 0.001.

To quantitatively assess the anti‐fibrotic shielding effect of the HAD hydrogel, two different adhesive lap shear tests were conducted. For the “sol‐adhesive” process, HAD precursors were placed between two pieces of fresh, defatted pig skin followed by UV light application for photocrosslinking, ensuring tight adhesion of the HAD between the two pig skin pieces (**Figure** **3**A); a commercially available fibrin glue (Greenplast) was used as the control group.^[^ ^13^ ^]^ Under the adhesion test of “sol‐adhesive” process, the adhesive strength of the HAD hydrogel was 14.58±1.10 kPa, while that of the commercial fibrin glue was ≈5.1 kPa±0.12 kPa, indicating that HAD possessed significantly higher adhesive strength (*p* < 0.01) (Figure 3C). For the “shield‐anti‐adhesive” process, HAD precursors were first completely crosslinked on one piece of the fresh porcine stomach, forming a smooth‐surfaced HAD gel. The second piece of the fresh porcine stomach was attached to the smooth surface of the crosslinked HAD hydrogel (Figure 3B). Under the adhesion test of “shield‐anti‐adhesive” process, the adhesive strength of the surface of the HAD hydrogel decreased to ≈0 kPa (*p* < 0.01) (Figure 3D). The results of these two tests demonstrate that, when HAD comes into contact with tissues, crosslinking can form an adhesive bond, whereas the outer surface, which is not in contact with the tissues, forms a smooth shield, providing an anti‐adhesive protective barrier for anti‐fibrotic repair.

Fibroblasts and macrophages are recruited by the immune system to promote postoperative adhesion.^[^ ^6^, ^7^, ^25^ ^]^ We conducted an in vitro assessment of the anti‐adhesive properties of HAD using L929 fibroblasts and RAW264.7 macrophages, commonly implicated in postoperative adhesions. These cells were applied to the surface of the HAD hydrogel and cells on a glass slide (TCP) served as a control. Live/dead staining revealed minimal round cells on the HAD hydrogel surface, in contrast to the substantial number of cells adhering to the TCP (Figure 3F). The cell proliferation assay confirmed that the limited number of L929 fibroblasts and RAW264.7 macrophages that adhered to the HAD hydrogel surface did not proliferate during the three‐day culture. In contrast, the same cells exhibited normal proliferation on the slide (Figure S8A, Supporting Information). Polyanionic HA impedes the attachment and spread of most mammalian cells because of its negatively charged surface, which repels negatively charged cell membranes.^[^ ^26^ ^]^ The DA groups served as a cell adhesion site. However, the photocrosslinked HAD hydrogels were securely embedded in the network, preventing them from promoting cell adhesion. Therefore, HAD hydrogels can serve as a shield against postoperative adhesion after crosslinking (Figure 3E). Due to the cell adhesion resistance of the HAD hydrogel, it also inhibits the maintenance of GATA6^+^ expression in the injured bladder following bladder defects, and inhibits fibrosis by downregulating the expression of inflammation (IL‐6, TNF‐α) (Figure 3G).^[^ ^7^ ^]^ We repaired rat bladder defects with HAD patches, and after the bladder was completely healed, the neotissue was collected (4 weeks postoperatively) for observing the GATA6^+^ macrophages adsorbed on the surface of HAD gel using immunofluorescence. The rat bladders treated with sutures served as the model group. In the model group, the bladder epithelium accumulated numerous GATA6^+^ macrophages. These cells identified and recruited each other through the positively charged type I scavenger receptor (MSR‐1), thereby forming a large amount of fibrous connective tissue (Figure 3H) (yellow dashed lines represent the severe recruitment of GATA6^+^ macrophages).^[^ ^6a^ ^]^ In the HAD group, only a few GATA6^+^ macrophages adhered to the surface of HAD (indicated by white arrows), without a large accumulation (Figure 3H; Figure S8B–D, Supporting Information). This may be due to the polyanionic HA in the HAD gel attracting the positively charged MSR‐1 through electrostatic attraction, which disrupts the mutual recognition among GATA6^+^ macrophages,^[^ ^22^ ^]^ thereby preventing the aggregation of GATA6^+^ macrophages at the wound site and inhibiting fibrosis.

### 2.3 Controlled Unidirectional Release of EVs for Wound Healing Via Photocrosslinking

**Figure 4:**
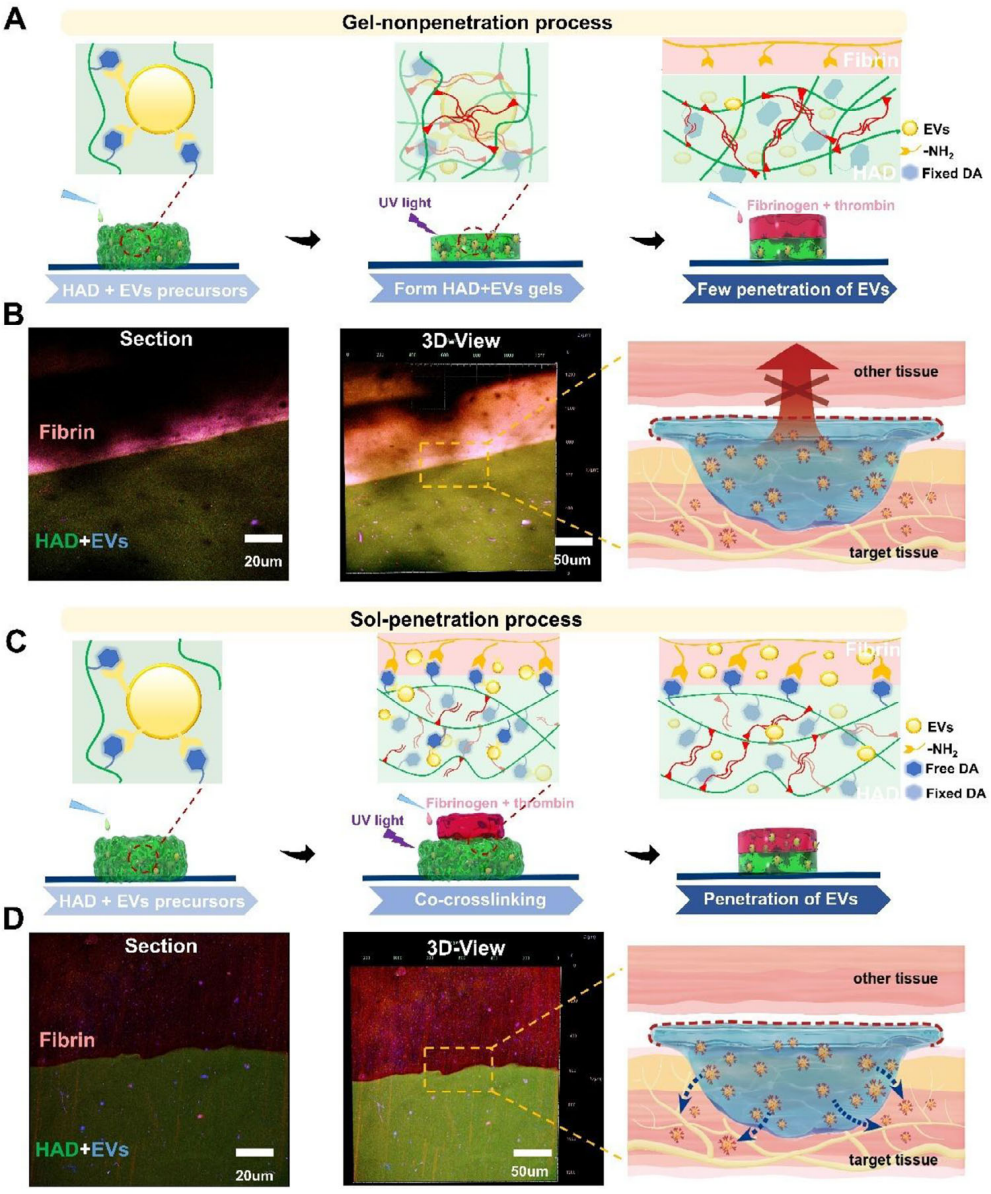
Controlled unidirectional release of EVs for wound healing via photocrosslinking. A) Schematic of the “gel‐non‐penetration” process. B) The HAD+EV hydrogel precursor was photocrosslinked first, followed by the formation of a fibrin gel on the surface of the HAD hydrogel, with few EVs penetrating the fibrin.C) Schematic of the “sol‐penetration” process. D) Upon contact of the HAD+EV hydrogel precursor with the fibrinogen solution, simultaneous gelation occurs, allowing the EVs within the HAD to penetrate the fibrin gel.

We designed a “gel‐non‐penetration” process and a “sol‐penetration” process to evaluate the bilateral EV‐controlled release effect of HAD+EV hydrogel after its in situ gelation in tissue wounds. In the “gel‐non‐penetration” process, the precursor solution of HAD+EV gel was first UV‐cured into a gel, followed by the addition of a fibrinogen solution and thrombin on the surface of the HAD gel to form a fibrin gel, which was left to stand for 5 days. Confocal microscopy was used to observe the interface between the HAD gel and the fibrin gel, revealing a clear and regular boundary, with no penetration or fusion between the two. The blue‐stained EVs were confined within the green HAD hydrogel area, and no blue fluorescence was observed in the red‐stained fibrin gel (**Figure** **4**B). This was attributed to the formation of an AEMA double‐bond network within the hydrogel upon UV light curing of the HAD precursor solution, which limited the number of free DA groups and consequently restricted the Michael addition reaction between the DA groups and the amine and thiol groups on the surface of the fibrin gel.^[^ ^12^, ^22^ ^]^ Crosslinking between DA and EVs also restricts the incorporation of EVs within the AEMA double‐bond network, making it difficult for them to penetrate the fibrin gel inside the HAD (Figure 4A).

In the “sol‐non‐adhesive” process, the HAD+EV hydrogel precursor was initially kept in contact with the fibrinogen solution, followed by UV irradiation and addition of thrombin. This allowed the HAD hydrogel precursor and fibrinogen solution to gel simultaneously, and the assembly was left to stand for 5 days. Confocal microscopy examination of the interface between the HAD hydrogel and the fibrin gel revealed intermingling and fusion of the two with a discontinuous boundary. The blue‐stained EVs penetrated the green‐labelled HAD hydrogel into the red‐labeled fibrin gel (Figure 4D). This is attributed to the presence of abundant free DA moieties in the flowable HAD hydrogel precursor. Upon direct contact between the HAD hydrogel precursor and the fibrinogen solution, DA promptly reacts with the amines and thiol groups on the surface of fibrinogen, resulting in covalent crosslinking. Concurrently, DA crosslinks with the amines on the surface of EVs, facilitating the co‐entry of EVs and free DA into the fibrin gel, thus establishing a unidirectional sustained release. Consequently, UV fixation performed after contact between the HAD hydrogel precursor and the fibrinogen solution reduced the constraint of the AEMA double‐bond network on DA and EVs (Figure 4C). Therefore, by controlling photocrosslinking, the HAD hydrogel can achieve a unidirectional sustained release of EVs into wounds, thereby enhancing the local concentration of EVs at the wound site.

### 2.4 S100 Protein Aptamer‐Modified EVs Targetedly Activate SCs Unveiling the Spear Effect

**Figure 5:**
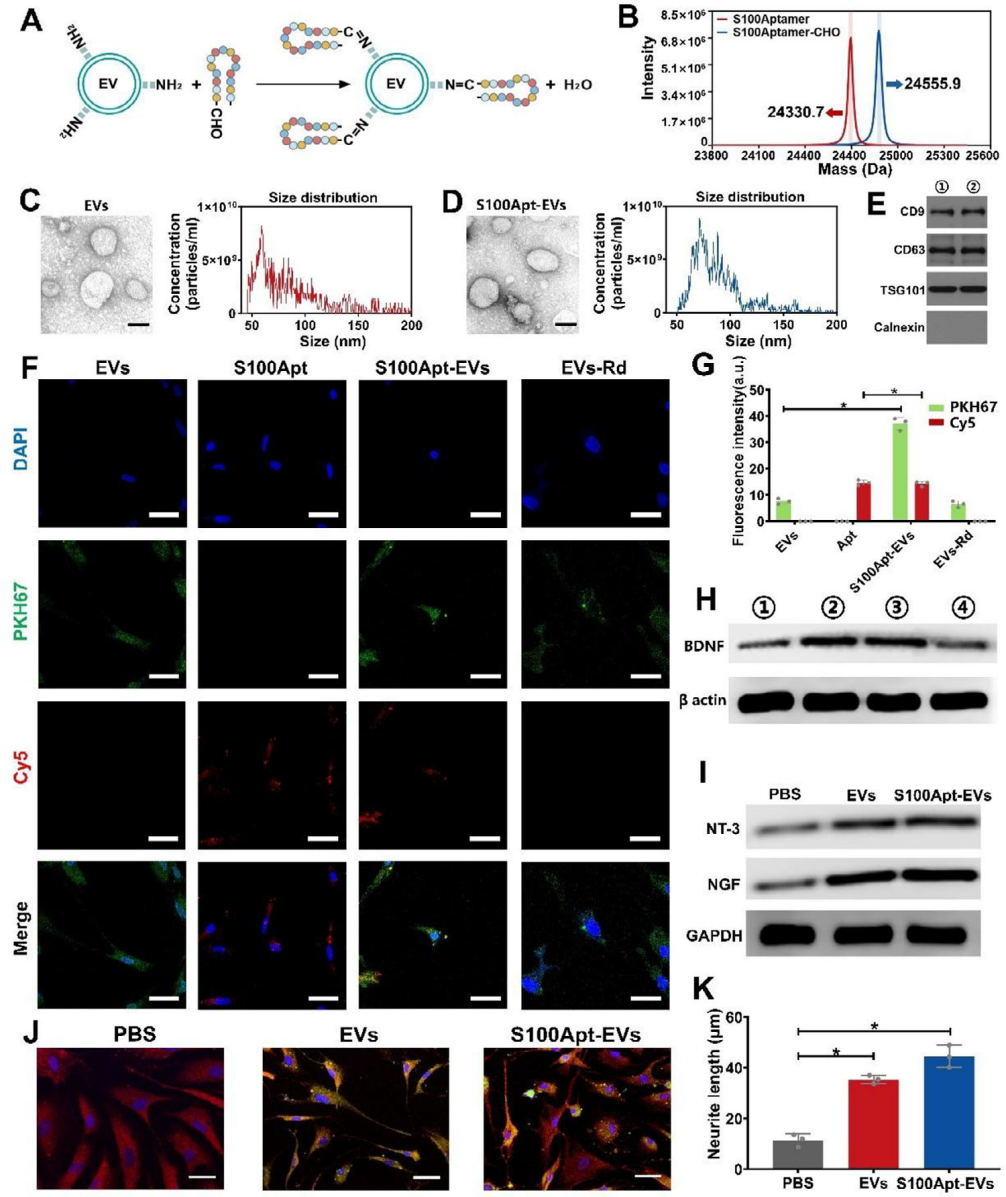
S100 protein aptamer enhances the efficiency of Schwann cell uptake of EVs, upregulates the expression of the anti‐fibrotic factor BDNF, and promotes nerve repair. A) Schematic representation of the chemical grafting between S100 aptamer and EVs. B) DNA mass spectroscopy shows the sequence of the S100 aptamer and the aldehyde‐modified S100 aptamer sequence. C) Transmission electron microscopy (TEM) analysis and diameter distribution of EVs. Scale bar = 100 nm. D) TEM analysis and diameter distribution of S100Apt‐EVs. Scale bar = 100 nm. E) Western blot analysis of protein markers (CD9, CD63, and TSG101) and the endoplasmic reticulum protein calnexin in EVs (①) and S100Apt‐EVs (②). F) Representative images of RSC96 Schwann cells internalizing PKH67‐labeled EVs (green) and Cy5‐labeled Apt (red). G) Quantitative analysis of fluorescence intensity (*p < 0.05). H) Differences in BDNF protein expression among various groups of RSC96 Schwann cells (①: PBS group; ②: S100Apt‐EVs; ③: S100Apt‐EVs + Proteinase K; ④: EVs). I) Protein expressions of neurotrophin‐3 and neurotrophic growth factor in Schwann cells among different groups. J) Immunofluorescence images of RSC96 cell morphology after co‐culture in different groups; EVs stained with PKH‐67 (green); cytoskeleton marked with Phalloidin‐Rhodamine (red). Scale bar = 100 µm. K) Branching length of RSC96 cells in different groups (*p < 0.05, n = 3 independent samples).

When systemically infused, unmodified EVs tend to accumulate rapidly in the liver and spleen, with limited distribution in the bladder.^[^ ^4^, ^27^ ^]^ The short circulation time of natural EVs impedes their effectiveness as therapeutic agents by preventing targeted delivery to damaged tissues.^[^ ^27^ ^]^ Owing to the presence of a specific S100 protein on the surface of SCs,^[^ ^20^ ^]^ we identified a nucleic acid aptamer sequence (S100Aptamer) capable of specifically recognizing SCs from an ssDNA sequence library using cell SELEX technology (Figure S9A,B, Supporting Information).^[^ ^28^ ^]^ The 5′ end of S100Apt was modified with an aldehyde group (‐CHO), which reacted with the amino groups of EV membrane proteins to form stable Schiff bases (**Figure** **5**A,B).^[^ ^21^ ^]^ EVs derived from ASCs and S100Apt‐EVs were identified by Western blot analysis, nanoparticle tracking analysis (NTA), and transmission electron microscopy (TEM). TEM revealed that both EVs and S100Apt‐EVs were ≈110 nm in size and exhibited cup‐shaped morphologies (Figure 5C). NTA analysis showed that the size of the EVs was between 62.34 and 123.11 nm, while that of S100Apt‐EVs ranged from 67.35 to 128.66 nm, with similar particle intensities between the two groups (Figure 5D). Additionally, the lysates of EVs and S100Apt‐EVs exhibited high levels of characteristic TSG101, CD9, and CD63 (Figure 5E). Next, we investigated the potential of S100Apt to enhance EV uptake by SCs. We incubated EVs with Cy5‐labeled S100Apt‐CHO utilizing a random sequence (Rd) with an aldehyde group as the negative control. Additionally, we used PKH67‐labeled EVs to assess the differences in uptake between EVs specifically labeled with S100Apt and those labeled with Rd sequences within SCs. Using SCs, we observed a robust PKH67 fluorescence signal in SCs treated with S100Apt‐EVs. In contrast, these signals were only weak in SCs treated with EVs or EVs‐Rd (*p* < 0.05; Figure 5F,G). Additionally, SCs treated with S100Apt‐EVs or Apt exhibited similar strong Cy5 fluorescence signals, whereas no Cy5 fluorescence signal was observed in SCs treated with EVs or EVs‐Rd (*p* < 0.05; Figure 5F,G). These results suggest that the specific S100 protein aptamer sequence enhanced the efficiency of EV recognition and uptake by SCs. Because secretion of BDNF by activated SCs plays a crucial role in inhibiting bladder fibrosis,^[^ ^11b^ ^]^ we investigated the differences in BDNF protein expressions among the SC groups. Compared with SCs treated with PBS, those treated with EVs expressed higher levels of BDNF, while the S100Apt‐EV group exhibited the highest level of BDNF expression (Figure 5H). ELISA also confirmed that Schwann cells in the S100Apt‐EVs group expressed more BDNF (Figure S9C, Supporting Information). Interestingly, treatment with proteinase K to completely degrade proteins in S100Apt‐EVs did not affect BDNF expression in SCs, indicating that S100Apt‐EVs do not transmit signals between cells via proteins. SCs treated with S100Apt‐EVs showed the highest levels of NT‐3, NGF, and other neurotrophic factors (Figure 5I). Repairing SCs plays a crucial role in guiding axon growth toward target organs by forming regenerative tracks, known as Bungners.^[^ ^10^ ^]^ Thus, we examined the effects of different co‐culture treatments on the development of SC branch projections. Fluorescence imaging and semi‐quantitative analysis demonstrated that the application of S100Aptamer‐EVs notably enhanced both the length and number of branching SCs, thereby facilitating the formation of Bungners, promoting neural repair and inhibiting neurogenic fibrosis (Figure 5J,K).

### 2.5 Integrated ‘shield‐spear’ Patch Achieved Fibrosis‐Free Reconstruction in Rat Hemicastration

**Figure 6:**
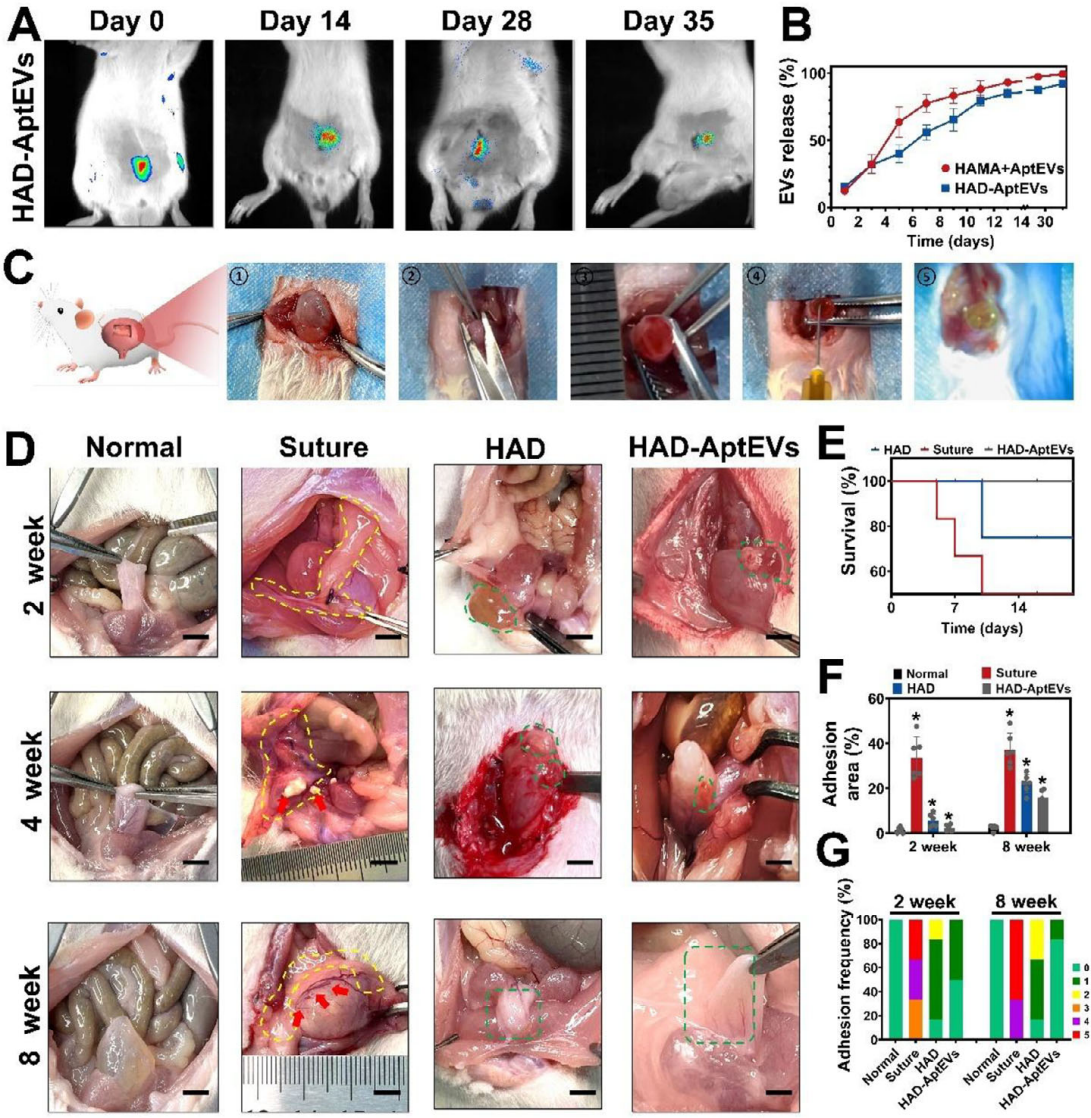
Fibrosis‐free healing with ‘shield‐spear’ Janus HAD‐AptEV patches in a rat hemisection bladder model. A) In vivo fluorescence imaging of rats in the HAD+EV and HAMA+EV groups at 35 days. B) Sustained release rate curves of EVs from HAD and HAMA hydrogels over 15 days in vitro. C) Schematic illustration of rat hemi‐bladder excision model and the sutureless sealing of extensive defects using ‘shield‐spear’ Janus HAD‐AptEV patches. D) Evaluation of bladder fibrosis and outer wall fibrous adhesion severity in Normal, Suture, HAD, and HAD‐AptEV groups at 2, 4, and 8 weeks. Scale bar: 5 mm. E) Survival rates of rats in each group within 20 days. F) Adhesion area percentage in each group. G) Fibrous adhesion scores at predetermined time points for each group. (*p < 0.05, n = 6 independent samples).

Before the formation of the HAD‐AptEV hydrogel, S100Apt‐EVs were stained with PKH26, and the HAD hydrogel was mixed with fluorescein isothiocyanate to facilitate the visualization of S100Apt‐EVs within the hydrogel. Fluorescence imaging showed that PKH26‐labeled S100Apt‐EVs were uniformly distributed within the HAD hydrogel (Figure S10, Supporting Information). The presence of DA in the HAD hydrogel resulted in the delayed release of EVs in vivo owing to its interaction with EVs (Figures S11 and S12, Supporting Information). Small animal in vivo imaging demonstrated that HAD maintained a stable and sustained release of EVs at the site of rat bladder defects for 35 days (**Figure** **6**A,B). Conversely, the absence of DA in the HAMA hydrogel led to the rapid release of EVs, as it lacked effective mechanisms to retain and restrict their release (Figures S11 and S12, Supporting Information).^[^ ^27^ ^]^

To evaluate the anti‐fibrosis effects of the ‘shield‐spear’ Janus HAD‐AptEV patch in vivo, we established a rat partial cystectomy model (Figure 6C). Rats were randomly assigned to the Normal, Suture (clinical suture treatment), HAD, or HAD‐AptEV groups (n = 6 per group, sampled at 2, 4, and 8 weeks). Upon laparotomy, we observed that surgical suturing could potentially cause puncture tissue damage in each group. Additionally, suturing procedures typically require at least 5 min, leading to challenging identification of bleeding points and prolonged surgical duration. In contrast, the HAD‐AptEV patch allowed for sutureless sealing of bladder fistulas and bleeding within 15 s (Figure 6C). The mortality rate of the animals was concentrated in two weeks post‐surgery. The mortality rates of the rats in the Suture and HAD groups 2 weeks postoperatively were 50% and 16.7%, respectively, whereas all rats in the HAD‐AptEV group survived (Figure 6E). The extent of fibrous adhesions in each group was scored using a standardized scoring system.^[^ ^22^ ^]^ Gross observations revealed significantly smaller fibrous adhesions and tissue calcification areas in the HAD‐AptEV group at 2, 4, and 8 weeks postoperatively than in the other groups (Figure 6D).

Two weeks later, the Suture group showed severe fibrous adhesions around the suture sites. The fibrous adhesion area in the Suture group involved 40% of the laparotomy area (Figure 6F), with an average adhesion score of 4 ± 0.1 (Figure 6G). Rats treated with HAD alone showed significant repair of bladder defects. Gross observations revealed that the degraded gel remnants in the HAD group were easily identifiable, maintaining a strong and stable adhesion to the bladder during the gradual oxidation process (indicated by green dashed lines), ensuring bladder integrity and normal urine storage. Although varying degrees of adhesions were observed between the bladder defect and the abdominal wall, the adhesion area in the HAD hydrogel group was less than 1/8 of that in the Suture group, with average adhesion scores of 1 ± 0.1 (*p* < 0.05; Figure 6F,G). Despite slight calcification, the HAD group exhibited satisfactory wound healing, comparable to that in the Normal group. In the HAD‐AptEV group, the ‘shield‐spear’ patch firmly adhered to the bladder surface and completely covered the defect, resulting in a smooth and intact bladder morphology capable of full expansion, ensuring normal urine storage (Figure 6D). Furthermore, compared to other groups, rats in the HAD‐AptEV group exhibited the smallest area of adhesion, demonstrating the lowest adhesion score of 0.5 ± 0.1 (Figure 6F,G).

At 4 and 8 weeks postoperatively, the bladder in the Suture group exhibited significant fibrous thickening and exacerbated fibrous proliferation in the outer wall, resulting in abnormal adhesions among the bladder, intestine, and abdominal wall (Figure 6D, indicated by yellow dashed lines). The bladder in the Suture group was enveloped by fibrous tissue and significantly compressed by fibrous adhesion tissue, leading to an indistinct overall bladder contour. In the Suture group, the fibrous adhesion area peaked at eight weeks, involving over 45% of the entire abdominal surface area. The average fibrous adhesion score in the Suture group at 8 weeks was 4.7 ± 0.1 (*p* < 0.05;Figure 6G), and bladder stones began to form inside the bladder (Figure 6D, indicated by red arrows) (Figure S13, Supporting Information). The occurrence of bladder stones in the Suture group may be attributed to the inflammation induced by suturing, leading to fibrosis and thickening of the bladder wall, resulting in bladder dysfunction and urinary obstruction. At 4 and 8 weeks postoperatively, significant degradation of the patches was observed in both HAD and HAD‐AptEV groups, without significant inflammatory infiltration or fibrous adhesions. New bladder tissue was regenerated in the defects covered by patches in the HAD and HAD‐AptEV groups with bladder contours and morphology similar to those in the Normal group. The average adhesion scores 8 weeks postoperatively were 1.17 ± 0.1 and 0.17 ± 0.1 in the HAD and HAD‐AptEV groups, respectively (*p*<0.05; Figure 6G), with no significant difference in fibrous adhesion severity. However, mild calcification was observed in the bladder wall of the HAD group at 4 weeks, whereas no calcification or fibrous thickening occurred in the bladder wall of the HAD‐AptEV group within 8 weeks of surgery (Figure 6D)

### 2.6 Molecular Mechanisms of Fibrosis‐Free Bladder Reconstruction Achieved by ‘shield‐spear’ Janus HAD‐AptEVs

**Figure 7:**
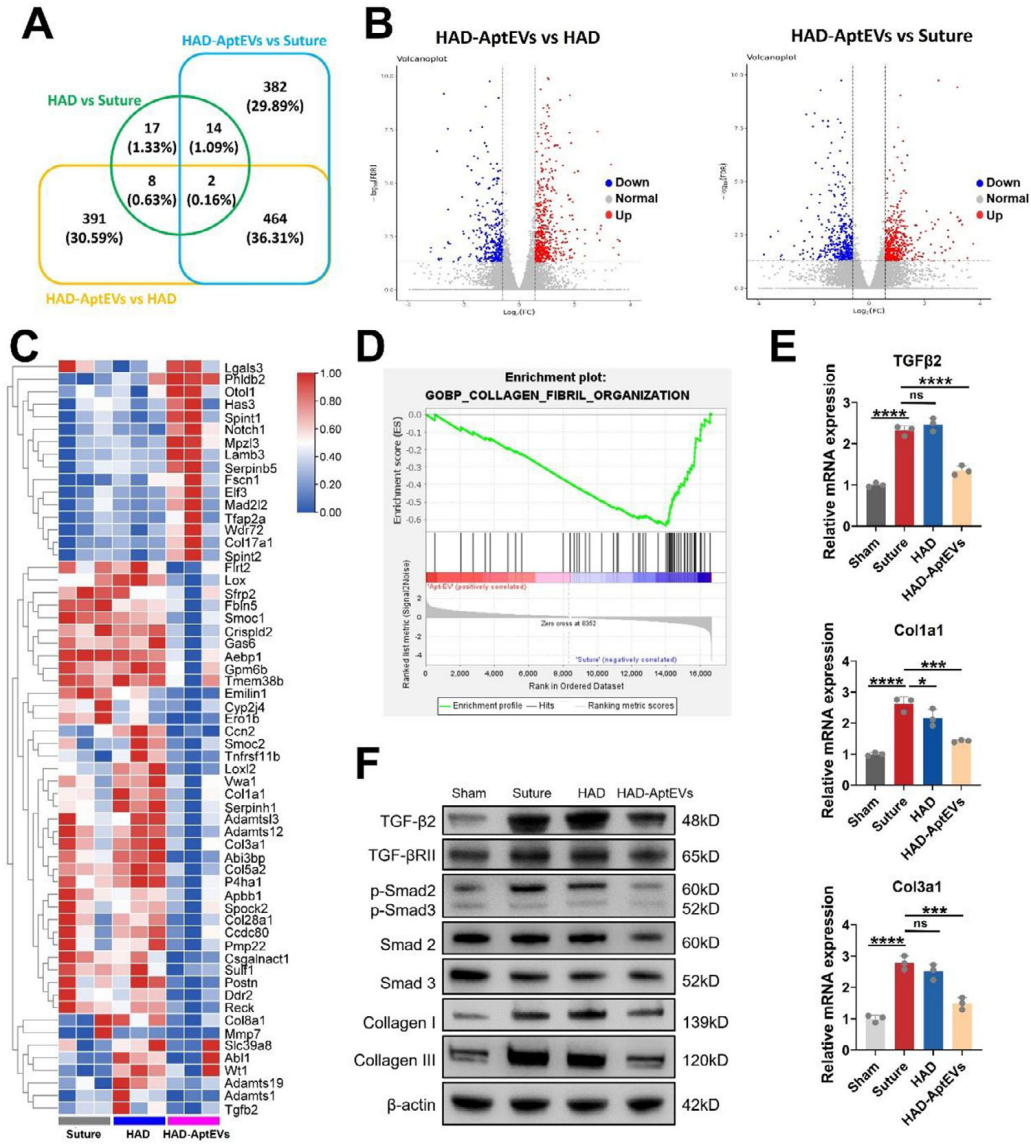
Molecular mechanisms of bladder fibrosis inhibition by the integrated ‘shield‐spear’ HAD‐AptEV patch. A) Venn diagram showing the differentially expressed genes in bladder tissues of rats in the Suture, HAD, and ‘shield‐spear’ HAD‐AptEV groups. B) Volcano plots illustrating the differentially expressed genes between HAD‐AptEV versus Suture and HAD groups. C) Heatmap depicting differentially expressed genes related to extracellular matrix assembly in bladder tissues of rats in Suture, HAD, and HAD‐AptEV groups. D) Enrichment plot of the gene set ollagen fibril organization" from Gene Set Enrichment Analysis comparing HAD‐AptEVs versus HAD, showing a downregulation trend. E) mRNA levels of Tgfb2, Col1a1, and COL3a1 in healing bladder tissues of each group 4 weeks postsurgery. F) Protein expression (TGF‐β2, TGF‐βRII, p‐Smad2, p‐Smad3, Smad2, Smad3, COLI, and COLIII) in healing bladder tissues of each group at 4 weeks post‐surgery, revealing the molecular mechanisms of fibrosis‐free bladder remodeling by inhibiting the TGFβ signaling pathway. n = 3 biological replicates. Data are presented as mean ± standard deviation. Error bars represent standard deviation. *p < 0.05, ***p < 0.001, ****p < 0.0001. ns = not significant. Source data are available as a Source Data file.

To further explore the potential molecular mechanism by which the HAD‐AptEV hydrogel achieves fibrosis‐free adhesion wound healing, high‐throughput sequencing via RNA‐Seq was conducted to examine the transcriptomic levels of tissues at the bladder defect repair site 4 weeks postsurgery across the HAD‐AptEV, HAD, and Suture groups. Differentially expressed genes (DEGs) were screened (selection criteria of log2FC > 0.585, *p* < 0.05). In total, 1278 DEGs were identified by pairwise comparison among the three groups. Venn diagrams displayed the overall changes and overlaps in DEGs between HAD‐AptEV versus HAD, HAD‐AptEV versus Suture, and HAD versus Suture groups (**Figure** **7**A). As shown in volcano plots, there were 506 upregulated genes and 360 downregulated genes between the HAD‐AptEV and HAD groups, whereas there were 430 upregulated and 432 downregulated DEGs between the HAD‐AptEV and Suture groups (Figure 7B). Between the HAD and Suture groups, there were only 17 upregulated and 24 downregulated DEGs, which were relatively fewer in number. This suggests that the impact and regulatory effects of HAD‐AptEVs on the transcriptome of injured bladder tissues are primarily achieved through the aptamer‐modified EVs carried by them.

As discussed previously, the in vivo experiments indicate that the HAD‐AptEV composite material exerted a significant inhibitory effect on the fibrosis healing process of bladder injuries. To corroborate this conclusion using transcriptomic analysis, DEGs related to the ECM assembly were analyzed. A total of 60 DEGs were involved in the ECM assembly process, and their expression levels across different groups are illustrated using a heatmap (Figure 7C). Notably, several fibrosis markers (such as Col1a1, Col3a1, Lox, and Loxl2) and the fibrosis‐related molecule TGF‐β2 were significantly downregulated in the HAD‐AptEV group. Furthermore, using the Gene Set Enrichment Analysis (GSEA) tool, gene expression enrichment in the “Collagen fibril organization” category of the Gene Ontology (GO) enrichment analysis between HAD‐AptEVs and HAD was analyzed. The results indicated that the HAD‐AptEV group showed a significant downtrend in the expression of this gene set (NES = −2.64), with the enrichment map presenting a typical trough shape (Figure 7D). To confirm the accuracy of the transcriptome sequencing analysis, qPCR was employed to quantify the mRNA levels of three representative DEGs, namely Tgfb2, Col1a1, and Col3a1, in each group. The results validated the downregulation of these indicators at the gene expression level, mediated by HAD‐AptEVs (Figure 7E). To further elucidate the regulatory details of the ‘shield‐spear’ HAD‐AptEV gel patch in the anti‐fibrotic process, we investigated some key genes within the TGF‐β signaling pathway, including TGF‐β2, Smad2, Smad3, Smad4, COL I, and COL III. Western Blot analysis at the protein level confirmed that HAD‐AptEVs could significantly reduce the level of TGF‐β2 in the injured bladder tissues and decrease the phosphorylation level of downstream Smad2/3 as well as the accumulation of COL I and COL III. The downregulation of the TGF‐β/Smad signaling pathway also accounts for the observed reduction in wound fibrosis during the healing process (Figure 7F).^[^ ^10^, ^29^ ^]^

**Figure 8:**
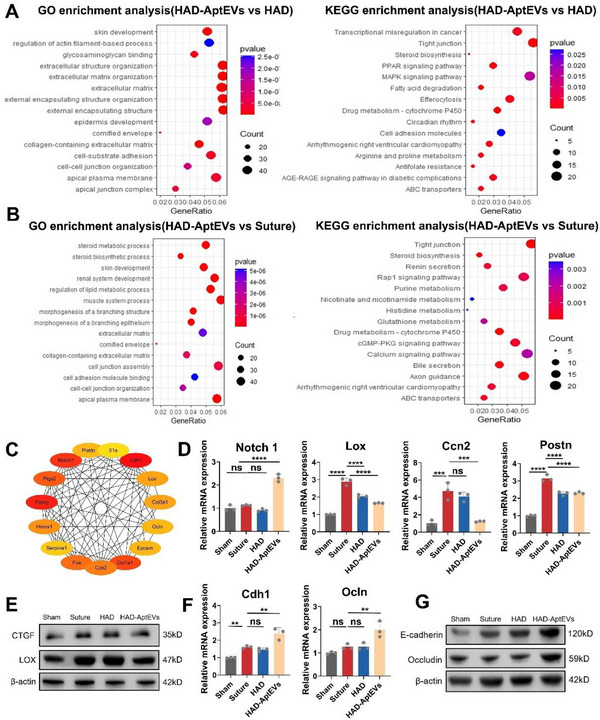
Molecular mechanisms of bladder defect healing by the integrated “Shield‐spear” HAD‐AptEV patch. A, B) Top 15 enriched gene sets from Gene Ontology and Kyoto Encyclopedia of Genes and Genomes enrichment analyses of differentially expressed genes between HAD‐AptEV and HAD groups A) and Suture group B) in rat bladder tissues. C) Top 15 hub genes (MNC method) from differentially expressed genes between HAD‐AptEV and HAD groups. D) mRNA expression levels of extracellular matrix organization‐related hub genes (Postn, Lox, Ccn2, and Notch1). E) Protein expression levels of fibrosis‐related hub genes (CTGF and LOX). F) mRNA expression levels of cell adhesion molecules‐related hub genes (Cdh1 and Occludin). G) Protein expression levels of hub genes regulating cell‐cell adhesion (E‐cadherin and Occludin), revealing the molecular mechanisms of bladder defect healing by “Shield‐spear” HAD‐AptEV hydrogel by promoting cell adhesion and enhancing cell barrier function. n = 3 biological replicates. Data are presented as mean ± standard deviation. Error bars represent standard deviation. **p < 0.01, ***p < 0.001, ****p < 0.0001, and ns = non‐significant. Source data are available as a Source Data file.

To uncover the potential regulatory mechanisms and pathways by which HAD‐AptEVs inhibit bladder fibrosis, gene enrichment analysis was conducted on DEGs identified by RNA‐seq. **Figure** **8**A presents the enrichment of the top 15 gene sets identified through GO and Kyoto Encyclopedia of Genes and Genomes (KEGG) enrichment analyses of DEGs between HAD‐AptEVs and HAD. Consistent with previous conclusions, the GO enrichment analysis highlighted entries predominantly associated with “ECM organization” and “collagen‐containing ECM.” This elucidates the anti‐fibrotic mechanism of the ‘shield‐spear’ HAD‐AptEV patch from a gene enrichment perspective.

Additionally, it is worth discussing that in the RNA‐Seq analysis, the simple HAD group did not exhibit significant anti‐fibrotic effects within the bladder tissue at the site of the bladder defect. This is because HAD primarily exerts its effects by inhibiting the accumulation of GATA6^+^ macrophages in the peritoneal cavity and reducing the recruitment of fibroblasts to diminish bladder adhesions, thereby isolating the bladder from extravesical pro‐fibrotic factors. However, simple HAD cannot modulate the microenvironment within the bladder tissue at the site of the defect. In contrast, HAD‐AptEVs preferentially release AptEVs to the wound site, targeting and promoting SCs to secrete BDNF, inhibiting the activation of fibroblasts and smooth muscle cells within the bladder, and reducing collagen synthesis to mitigate the occurrence of neurogenic fibrosis. During bladder sampling, we separated the bladder from adhesions with other abdominal organs and only collected tissue from the periphery of the injury site. Therefore, we could not observe the anti‐fibrotic effects of HAD, while HAD‐AptEVs still demonstrated excellent anti‐fibrotic properties.

Additionally, it is noteworthy that GO analysis identified enrichments in “cell‐cell junction organization,” and KEGG analysis indicated enrichments in “tight junction” and “cell adhesion molecules,” among other entries. This suggests that in the bladder tissue of the ‘shield‐spear’ HAD‐AptEV group, cellular adhesion and connections, as well as other life activities, were significantly regulated. Cell adhesion plays an important regulatory role in tissue repair; cell adhesion molecules not only facilitate adhesion and aggregation between cells, but also regulate cell migration, differentiation, and proliferation through signal transduction.^[^ ^30^ ^]^ E‐cadherin, a cell adhesion protein, plays a crucial role in maintaining cell‐cell adhesion and polarity of epithelial cells.^[^ ^31^ ^]^ Occludin, an important protein in tight junctions, is upregulated, indicating enhanced cellular barrier functions.^[^ ^32^ ^]^ Strengthening tight junctions contributes to the prevention of bacterial invasion and maintenance of the stability of the microenvironment, which is particularly important for organs such as the bladder, which stores urine. Western Blot results showed that by upregulating the protein expression of Occludin, the ‘shield‐spear’ HAD‐AptEV gel patch may help accelerate the self‐repair process of bladder tissue, restore the integrity of tight junctions, and thus promote the structural and functional recovery of the bladder (Figure 8G). Moreover, Western Blot results confirmed that, compared to the sole HAD gel, the introduction of S100Apt‐EVs may further enhance the adhesion between bladder epithelial cells (Figure 8G), strengthening cell‐cell adhesion and tight junctions, which contributes to the restoration of the integrity and function of the urinary epithelium. Such tight cell connections are essential for the integrity and functional repair of bladder tissue. In the GSEA conducted using the GSEA tool to assess the differences in gene expression between the HAD‐AptEV and HAD groups, downregulation of entries related to fibrosis, such as “collagen fibril organization” and “collagen‐containing ECM,” was enriched (Figure S14A, Supporting Information), along with the upregulation of entries related to cell adhesion, such as “cadherin binding” in cell‐cell adhesion (Figure S14B, Supporting information). Figure 6b shows the GO and KEGG enrichment analyses of DEGs between HAD‐AptEVs and Sutures. Using GSEA, the conclusions derived from the GO analysis of the gene expression differences between these two groups were similar to those observed when comparing HAD‐AptEVs and HAD (Figure S15, Supporting Information).

To further clarify the core genes that regulate HAD‐AptEV resistance to fibrosis and promote wound healing, the DEGs between HAD‐AptEVs and HAD were analyzed using the STRING tool to construct a protein‐protein interaction network. The network was then imported into the Cytoscape software, and the top 15 hub genes were selected using the Cytohubba plugin with the MNC method (Figure 8C). Among the 15 core genes identified, there were five downregulated genes (Col1a1, Col3a1, Postn, Lox, and Ccn2), one upregulated gene (Notch1) associated with ECM organization, and two upregulated genes (Cadherin1 and Occludin) associated with cell adhesion molecules. The mRNA expression levels of these core genes were validated using qPCR (Figure 8D,F), and the protein levels of the selected genes were assessed using Western Blotanalysis (Figure 8E,G). This provides further evidence that HAD‐AptEVs may play a crucial role in downregulating the TGF‐β/Smad pathway, which is essential in inflammation‐associated tissue fibrosis, thereby reducing collagen deposition and tissue fibrous adhesion proliferation; additionally, by modulating cell‐cell adhesion and tight junctions, the integrated ‘shield‐spear’ HAD‐AptEV patches promote the healing of bladder injuries, thus balancing the contradiction between healing and anti‐fibrosis from two aspects, ultimately achieving fibrosis‐free bladder reconstruction.

### 2.7 Fibrosis‐free Bladder Reconstruction of the Integrated ‘shield‐spear’ HAD‐AptEV Patch in Beagle Models

**Figure 9:**
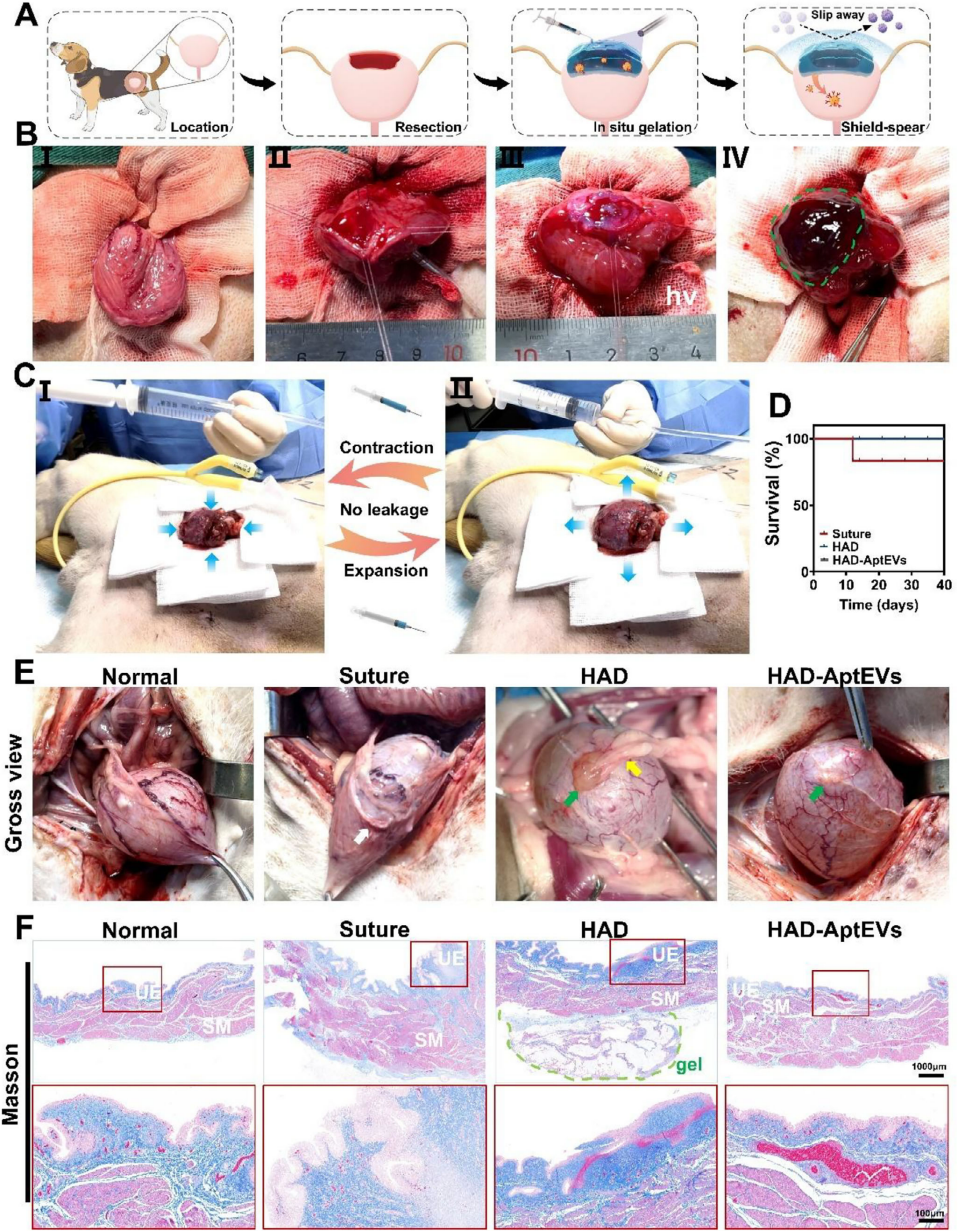
Bladder fibrosis‐free reconstruction using the integrated ‘shield‐spear’ HAD‐AptEV biological patch in beagle bladder defects. A, B) Schematic representation A) and actual image B) of beagle bladder defect modeling and HAD‐AptEV patch repair process. C) Illustration of bladder expansion and contraction after 4 weeks of HAD‐AptEV patch repair in beagles. Physiological saline injection and aspiration through the urinary catheter into the bladder were performed to observe bladder expansion and contraction, with no observed leakage or bleeding. D) Survival curves of beagles in each group 4 weeks postoperatively. E) Macroscopic images of bladder healing in beagles in each group 4 weeks postoperatively; white arrows indicate fibrotic hyperplasia of the bladder wall tissue; green arrows indicate gel fragments adhering to the bladder surface; yellow arrows indicate postoperative fibrous adhesion tissue. F) Masson's trichrome‐stained sections of tissue at the site of bladder defect healing in each group of beagles after 4 weeks (scale bars: 1000 µm for the upper row; 100 µm for the lower row); UE represents urothelium; SM represents smooth muscle.

The in vivo effect of the integrated ‘shield‐spear’ HAD‐AptEV biological patch on promoting bladder fibrosis‐free reconstruction was investigated in a beagle model (**Figure** **9**A). Beagles were anesthetized with propofol, followed by abdominal preparation and disinfection (Figure S16, Supporting Information). After laparotomy and bladder exposure, a 2 cm × 2 cm penetrating defect was created on the bladder wall using surgical scissors, followed by injection of the precursor HAD hydrogel solution to completely cover the bladder defect, and subsequent in situ crosslinking with UV light to achieve complete sealing of the defect (365 nm LED, 20 mW cm^−2^, 3 s) (Figure 9B; Movie S2, Supporting Information). The model was validated against clinical scenarios. In this study, the 30% bladder wall excision mimics trauma‐induced muscle loss and post‐TURBT (transurethral resection) defects. During the procedure of treating bladder defects with the HAD gel, physiological saline was repeatedly injected and aspirated through a urinary catheter inserted into the bladder to observe successful contraction and expansion of the bladder treated with HAD (Figure 9C; Movie S3, Supporting Information), confirming the good sealing properties of the HAD gel patches, even in beagle bladders with thicknesses similar to those of human bladders. Successful internal leakage of blood and urine was also observed after sealing the bladder defect with HAD (Figure S17, Supporting Information). Beagles in the Suture group underwent traditional surgical suturing at the bladder defect site (Figure S18, Supporting Information). Postoperatively, the beagles underwent cardiac monitoring and urinary catheter placement, among other treatments (Figure S19, Supporting Information). Survival curves of beagles in each group are shown in Figure 9D. One beagle in the Suture group died on postoperative day 11 due to excessive fibrotic adhesion formation between the bladder suture site and intra‐abdominal intestinal tissues, as well as anastomotic site infection (Figure S20A,B, Supporting Information), whereas beagles in the remaining groups were in a normal condition.

Four weeks postoperatively, gross observations revealed that beagles in the Suture group exhibited the most severe fibrotic adhesion, characterized by proliferative connective tissue formation at the suture sites, connecting the bladder to the surrounding intestine and abdominal walls. Abnormal adhesions resulted in abnormal traction and restricted bladder movement, indicating relatively poor repair (Figure 9E, white arrows indicate fibrotic thickening). In the HAD group, noticeable HAD fragments adhered to the bladder defect site. Along with bladder defect healing, some adhesions between the HAD surface and intestinal tissues were observed, albeit less severe than those in the Suture group, with no apparent fibrotic scar formation (Figure 9E, green arrows indicate residual HAD at the top of the bladder; yellow arrows indicate fibrotic adhesions). Bladder repair in the HAD‐AptEV group was markedly superior to that in the other groups, with a smooth bladder wall surface, absence of proliferative fibrotic connective tissue formation, and no adhesions between the peritoneum and other organs. Most gel patches degraded with wound healing, with a few degraded fragments adhering to the top of the bladder and gradually oxidizing to a yellow‐brown color (Figure 9E, green arrows represent residual HAD‐AptEV patches on the top of the bladder). Bladder motility and voiding were normal in the HAD‐AptEV group with no tissue traction or restriction.

An essential criterion for evaluating bladder structural restoration is the continuous repair of the urothelium (UE) lining the bladder wall.^[^ ^4^, ^14^ ^]^ Four weeks after surgery, the newly formed bladder tissues in each group were histopathologically analyzed using Masson's trichrome staining. The repair level of the bladder wall UE and collagen deposition were also assessed (Figure 9F; Figure S20C, Supporting Information). In the Suture group, although the sutured UE layer healed and appeared continuous, there was extensive collagen deposition between the newly formed UE layer and the smooth muscle layer (SM), resulting in fibrosis (the blue‐stained area was significantly larger than that in the Normal group). Abundant collagen deposition between the SM and UE layers of the bladder wall is a classical mechanism underlying bladder fibrosis,^[^ ^8^ ^]^ consistent with the macroscopic observation of postoperative fibrous hyperplasia (Figure 9E). In the HAD group, residual HAD gel was attached to the newly formed bladder outer wall. Compared to the Suture group, there was less collagen deposition in the newly formed bladder UE and SM layers in the HAD group, with limited inflammatory cell infiltration. However, compared to the Normal group, the newly formed UE layer in the HAD group appeared discontinuous and intermittently arranged, with a few newly formed blood vessels. Under the synergistic action of Apt‐EVs, the bladder repair area in the HAD‐AptEV group exhibited abundant blood vessels, regenerating continuous and intact UE and SM layers on the bladder luminal surface that closely resembled the structure in those in the Normal group. There was no excessive fibrous adhesion on the outer wall, indicating scarless bladder reconstruction. These gross and histological observations demonstrate that our designed ‘shield‐spear’ Janus bladder patch offers the advantages of simplicity, satisfactory adhesion, and adaptation to the dynamic urethral microenvironment, providing durable protection against bladder injury. Under the synergistic effects of HAD anti‐collagen attachment and Apt‐EVs promoting SC activation, the integrated ‘shield‐spear’ Janus HAD‐AptEV patch can effectively seal bladder defects in a dynamic and moist environment and achieve anti‐fibrotic bladder healing.

**Figure 10:**
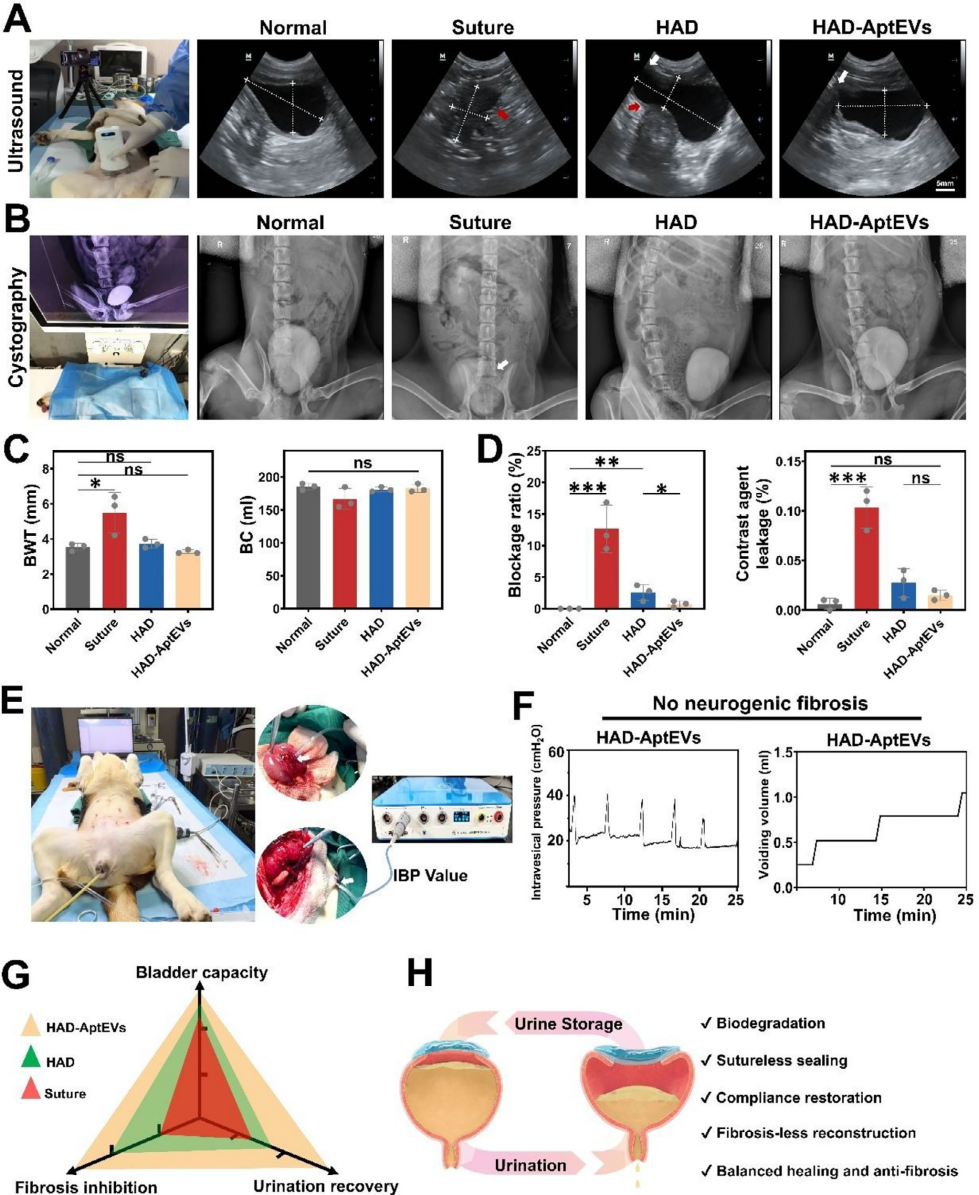
Functional assessment following bladder reconstruction with the integrated ‘shield‐spear’ HAD‐AptEV patch. A) Ultrasound images of the beagle bladders in each group 4 weeks postoperatively. White arrows indicate the site of material sealing, while red arrows indicate areas of adhesive tissue compression. White lines represent the measured distances along the longitudinal and transverse axes of the bladder. Scale: 5 mm. B) Retrograde cystography images of beagles 30 minutes after contrast agent injection, 4 weeks postoperatively. White arrows indicate the site of material sealing. C) Average bladder wall thickness and bladder capacity of the beagles in each group. BWT: bladder wall thickness; BC: bladder capacity. D) Rates of obstruction and contrast agent leakage in each group 4 weeks postoperatively, measured and analyzed using ImageJ software; white arrows denote areas of bladder filling defects in the Suture group. E‐F) Urodynamic testing of beagles in each group 4 weeks postoperatively E), with urodynamic analysis of the HAD‐AptEV group F). G) Degrees of bladder capacity, voiding recovery, and anti‐fibrosis in the Suture, HAD, and HAD‐AptEV groups. H) The integrated ‘shield‐spear’ HAD‐AptEV patch demonstrates rapid crosslinking, stable wet adhesion, safe degradation, and balanced inhibition of collagen deposition to facilitate bladder reconstruction. All data are presented as mean ± SD from at least three independent experimental samples. *p < 0.05, **p < 0.01 and ***p < 0.001. ns = no significance.

In ultrasonographic urethral image, the Suture group exhibited localized thickening of the bladder wall at the suture site, suggesting significant inflammation and fibrosis. This led to apparent narrowing, possibly due to inflammatory infiltration promoted by suturing, resulting in fibrous adhesion and subsequent bladder constriction (**Figure** **10**; Figures S20 and S21, Supporting Information). Ultrasound images showed unclear bladder delineation and increased high‐density noise in the Suture group, indicating extensive fibrous proliferation and tissue adhesion enveloping the outer bladder wall (Figure 10A, red arrows indicate fibrous adhesion compression). Additionally, slight high‐density shadows were observed in the bladder in the Suture group, suggesting the presence of minor calculus formation attributable to decreased bladder compliance, restricted mobility, urinary difficulties, and prolonged urine retention caused by fibrous proliferation. The HAD group demonstrated increased bladder wall thickness compared to the Normal group (Figure S21, Supporting Information), but it was significantly less than the Suture group, implying the anti‐inflammatory and fibrosis‐reducing effects of HAD. However, noticeable adhesions between the adjacent prostate tissue and the bladder were observed, resulting in slight constriction of the bladder due to compression (Figure 10A–C, white arrows denote the HAD sealing site; red arrows denote compression from adjacent tissue). Conversely, the bladder lumen in the HAD‐AptEV group appeared patent and well filled, with bladder wall thickness and capacity closely resembling those of the Normal group (Figure 10C; Figure S21, Supporting Information), suggesting a significantly enhanced anti‐inflammatory and fibrosis‐inhibiting capability conferred by the addition of AptEVs to the patch.

Unlike other imaging technologies,^[^ ^33^ ^]^ urethrography was specifically performed to assess bladder tissue regeneration.^[^ ^34^ ^]^ At the 15th minute (Figure S22, Supporting Information) and 30th minute (Figure 10B) of urethral contrast radiography, X‐ray contrast imaging revealed bladder filling defects and contrast agent leakage in the Suture group, suggesting internal bladder obstruction and incomplete repair. Neither the HAD nor the HAD‐AptEV group exhibited significant filling defects or leakage (Figure 10D). Compared to the Suture group, the bladder walls in the HAD and HAD‐AptEV groups appeared more uniform and continuous on imaging, with good contrast agent filling, resembling those in the Normal group without apparent bladder contour depression (Figure 10B–D), indicating the effective promotion of structural repair of bladder defects by the HAD gel and the integrated ‘shield‐spear’ HAD‐AptEV patch.

Four weeks postoperatively, bladder catheterization was performed in all groups, and bladder urodynamic assessments were performed (Figure 10E; Figure S23, Supporting Information). In the HAD‐AptEV group, a regular voiding cycle was observed with no signs of obstruction or excessive activity during the storage and voiding processes. The bladder function pattern in the HAD‐AptEV group resembled that in the Normal group (Figure S24, Supporting Information). In contrast, the urodynamic curve of the Suture group showed a continuous increase in bladder pressure without a sudden drop in pressure, indicative of bladder dysfunction and impaired voiding capacity, which could be attributed to decreased compliance due to bladder wall fibrosis (Figure S24, Supporting information). The urodynamic performance of the HAD group was similar to that of the Normal and HAD‐AptEV groups, with pressure peaks observed during the storage and voiding phases. However, in the second voiding cycle, the voiding peak appeared erratic and unstable, suggesting the possibility of bladder spasms in the HAD group, which may be associated with mild bladder fibrosis (Figure S24, Supporting Information). By comparing bladder urodynamic test results with contrast‐enhanced imaging results, we found that the restoration of bladder capacity, voiding recovery, and fibrosis inhibition in the HAD‐AptEV group correlated positively with improvements in imaging and urodynamics (Figure 10G). Overall, these gross and quantitative functional assessments demonstrate that the integrated ‘shield‐spear’ HAD‐AptEV patch effectively promoted the restoration of bladder compliance, achieving normal storage and voiding, highlighting the potential clinical value in achieving fibrosis‐free bladder reconstruction (Figure 10H).

**Figure 11:**
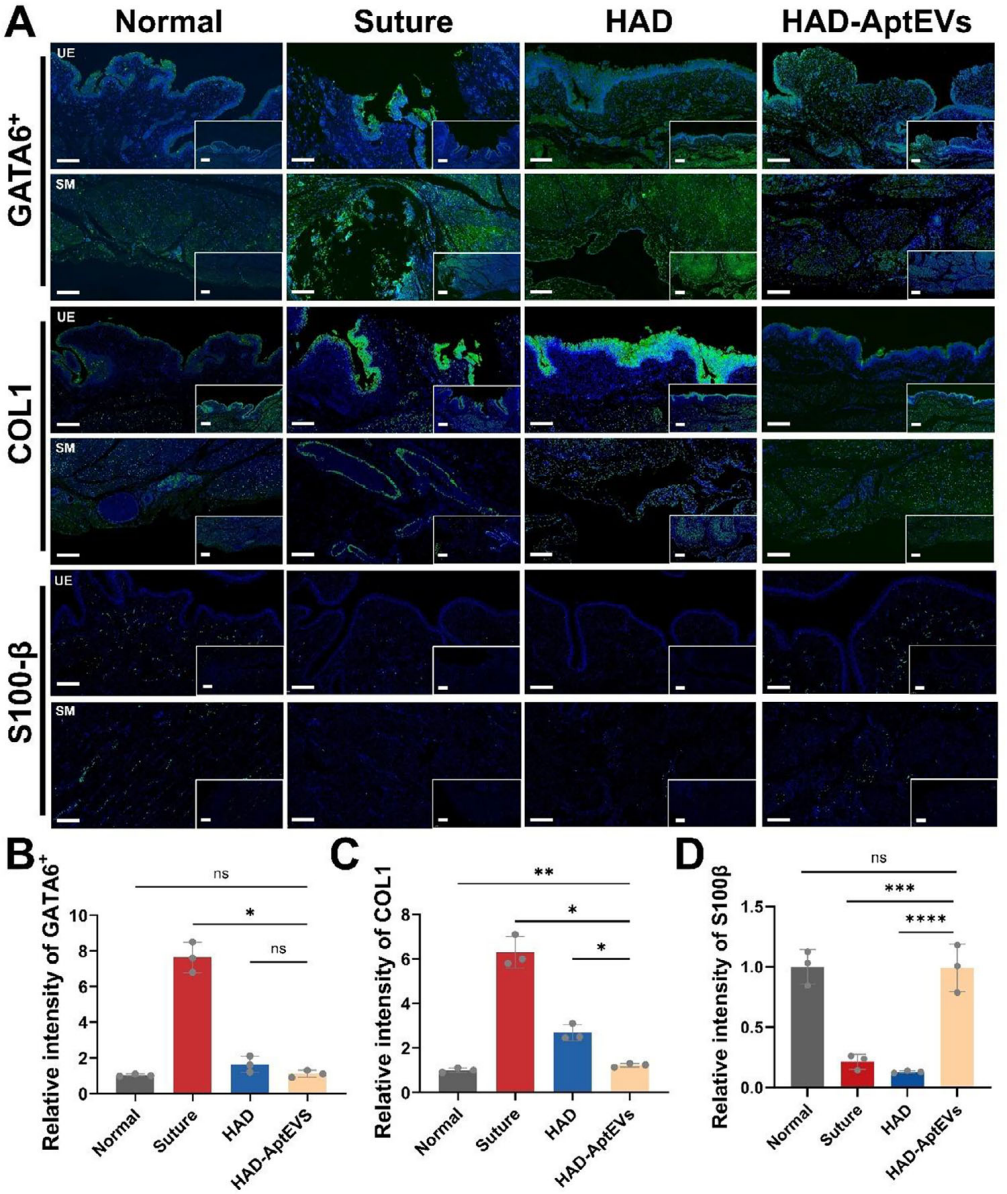
Immunofluorescent examinations of fibrosis‐free wound healing of the beagle bladders. A) Immunofluorescence staining of the beagle bladder for evaluating collagen deposition (GATA6^+^) and fibrogenesis (COL1 and S100β) after different treatments (i.e., Suture, HAD, HAD‐AptEVs and Normal groups) for 4 weeks. Scale bar for the large image is 200 µm; scale bar for the small image is 500 µm. B–D) Quantitative expression levels of b) GATA6^+^ macrophages, C) COL1, and D) S100β after different treatments (i.e., Suture, HAD, HAD‐AptEVs and Normal groups) for 4 weeks. The hydrogel dressing compositions are the same as in Figure 10. n = 3 biologically independent samples. Data are presented as mean ± SD. All data are presented as mean ± SD from at least three independent experimental samples. All error bars represent SD. *p < 0.05, **p < 0.01, ns = no significance.

Immunofluorescence examinations were used to further evaluate protein expression in the repaired bladders (**Figure** **11**). The Suture group showed uncontrolled aggregation of GATA6^+^ macrophages in the urothelium and smooth muscle layer (Figure 11A,B), with highly expressed collagen I protein (the marker of myofibroblasts. This indicates that the suture treatment leads to overproliferating myofibroblasts, deposited collagen fibers and inevitable fibrosis in the bladder wall (Figure 11A–C). The collagen I and GATA6^+^ expression level under the urothelium was relatively lower in the HAD and HAD‐AptEV groups than that in the Suture group, implying that the effective inhibition of the TGFβ/Smad signaling pathway is conducive to antifibrogenic function. This benefit is attributed to the anti‐GATA6^+^ activity of the anionic ‘shield’ provided by the HAD hydrogel. However, thin and discontinuous urothelial cells in the HAD group mean unsatisfactory barrier‐free healing outcomes and unfavorable wound recovery, which is attributed to the singular anti‐fibrotic effects of HAD. The HAD‐AptEV group not only moderately counteracted the deposition of fibroblasts and collagen through HAD but also activated the Cadherin signaling pathway via S100Apt‐EVs, promoting tight cell‐to‐cell connections. As a result, on the sections, the HAD‐AptEV group exhibited the highest expression level of S100β (Figure 11A–D). This accelerated the complete restoration of the bladder layers, resulting in morphology that closely resembles that of the Normal group. All these results suggested that the synergetic effect of both anionic ‘shield’ HAD hydrogel and S100Aptamer‐EVs has the potential to promote the regeneration of urothelium layers without connective tissue or hypertrophic scar formation.

## 3 Conclusion

In this study, we developed an integrated ‘shield‐spear’ HAD‐AptEV biological patch via Schiff base chemistry and Michael addition to address the dual challenges of fibrosis inhibition and tissue repair in large bladder defects. The anionic ‘shield’ hydrogel (HAD) suppresses abnormal ECM deposition by neutralizing TGFβ/Smad signaling and disrupting GATA6+ macrophage‐fibroblast crosstalk, while the SC‐targeted ‘spear’ (S100Apt‐EVs) promotes urothelial regeneration via Cadherin pathway activation. Leveraging differential adhesion properties, the Janus‐structured patch achieves directional EV release toward wound sites while resisting macrophage/fibroblast adhesion post‐photocrosslinking, thus resolving the paradox between ECM deposition and anti‐fibrotic requirements. In beagle models, the patch demonstrated rapid in situ gelation (<5 s), robust underwater adhesion, and sustained mechanical stability under dynamic bladder pressures. Postoperative evaluations confirmed complete defect healing without fibrosis, scar formation, or bladder wall hypertrophy, with restored urodynamic parameters comparable to healthy controls. Notably, the unified ‘shield‐spear’ design overcomes limitations of multilayer scaffolds, offering enhanced surgical manageability and biocompatibility. While preclinical results highlight its potential for fibrosis‐free reconstruction, clinical translation requires further validation of human‐specific responses. This work provides a paradigm‐shifting strategy for balancing tissue repair and fibrotic suppression in complex organ regeneration, with broad applicability to other dynamic tissue engineering scenarios.

## Conflict of Interest

The authors declare no conflict of interest.

## Supporting information



Supporting Information

Supporting Information

Supporting Information

Supporting Information

## Data Availability

The data that support the findings of this study are available in the supplementary material of this article.

## References

[1] a) A. Atala , S. B. Bauer , S. Soker , J. J. Yoo , A. B. Retik , Lancet 2006, 367, 1241; 16631879 10.1016/S0140-6736(06)68438-9

[2] H. Baumert , P. Simon , M. Hekmati , G. Fromont , M. Levy , A. Balaton , V. Molinié , B. Malavaud , Eur. Urol. 2007, 52, 884. 17229515 10.1016/j.eururo.2006.11.044

[3] a) A. Wierzbicka , M. Krakos , P. Wilczek , D. Bociaga , J. Biomed. Mater. Res. B. Appl Biomater. 2023, 111, 730; 36237176 10.1002/jbm.b.35179

[4] a) D. Xiao , M. Yang , M. Zhang , L. Rong , Y. Wang , H. Cheng , X. Sui , S. P. Sheikh , M. Lu , Chem. Eng. J. 2021, 425, 131624;

[5] a) S. J. Kim , J. Kim , Y. G. Na , K. H. Kim , Int. Neurourol. J. 2021, 25, S3; 34053205 10.5213/inj.2142174.087 PMC8171243

[6] a) J. Zindel , M. Peiseler , M. Hossain , C. Deppermann , W. Y. Lee , B. Haenni , B. Zuber , J. F. Deniset , B. G. J. Surewaard , D. Candinas , P. Kubes , Science 2021, 371, abe0595; 10.1126/science.abe0595 33674464

[7] S. Balog , S. Jeong , K. Asahina , Faseb. J. 2024, 38, 23327. 10.1096/fj.202301187R 38019178

[8] J. Liao , X. Li , Y. Fan , Bioact. Mater. 2023, 26, 387. 36969107 10.1016/j.bioactmat.2023.02.026 PMC10030827

[9] M. von Siebenthal , A. Akshay , M. Besic , M. P. Schneider , A. Hashemi Gheinani , F. C. Burkhard , K. Monastyrskaya , Int. J. Mol. Sci. 2023, 24, 2451. 36768773 10.3390/ijms24032451 PMC9916488

[10] S. Wang , Z. Wang , W. Yang , Z. Xu , H. Dai , F. He , S. Yan , X. Shi , Adv. Mater. 2024, 36, 2311264. 10.1002/adma.202311264 38330187

[11] a) P.‐Z. Hang , J. Liu , J.‐P. Wang , F.‐F. Li , P.‐F. Li , Q.‐N. Kong , J. Shi , H.‐Y. Ji , Z.‐M. Du , J. Zhao , Eur. J. Pharmacol. 2023, 938, 175420; 36427535 10.1016/j.ejphar.2022.175420

[12] a) X. Wu , Z. Wang , J. Xu , L. Yu , M. Qin , J. Li , S. Liu , W. Zheng , Z. Li , J. Ouyang , Y. Li , G. Li , L. Wang , W. Huang , Y. Wu , Theranostics 2023, 13, 5365; 37908723 10.7150/thno.87639 PMC10614681

[13] Z. Li , L. Liu , Y. Chen , Acta Biomater. 2020, 110, 119. 32438111 10.1016/j.actbio.2020.04.034

[14] Y. Zhao , Y. He , J.‐H. Guo , J.‐S. Wu , Z. Zhou , M. Zhang , W. Li , J. Zhou , D.‐D. Xiao , Z. Wang , K. Sun , Y.‐J. Zhu , M.‐J. Lu , Acta Biomater. 2015, 23, 91. 26049152 10.1016/j.actbio.2015.05.032

[15] G. Li , Q. Han , P. Lu , L. Zhang , Y. Zhang , S. Chen , P. Zhang , L. Zhang , W. Cui , H. Wang , H. Zhang , Research 2020, 2020, 2603048. 32851386 10.34133/2020/2603048 PMC7436332

[16] a) S. Zhu , L. Chen , M. Wang , J. Zhang , G. Chen , Y. Yao , S. Song , T. Li , S. Xu , Z. Yu , B. Shen , D. Xu , Z.‐L. Chi , W. Wu , J. Controlled Release 2023, 363, 641; 10.1016/j.jconrel.2023.10.012 37820984

[17] S.‐H. Chen , H.‐K. Kao , J.‐R. Wun , P.‐Y. Chou , Z.‐Y. Chen , S.‐H. Chen , S.‐T. Hsieh , H.‐W. Fang , F.‐H. Lin , APL. Bioeng. 2022, 6, 046103. 36345317 10.1063/5.0118862 PMC9637024

[18] a) D. Zheng , H. Ruan , W. Chen , et al., Bioact. Mater. 2023, 25, 500; 37056271 10.1016/j.bioactmat.2022.07.022 PMC10087114

[19] R. C. Ching , P. J. Kingham , Neural Regener. Res. 2015, 10, 743. 10.4103/1673-5374.156968 PMC4468764 26109947

[20] H. Shi , B. E. Hoffman , J. T. Lis , Proctl. Natl. Acad. Sci 1999, 96, 10033. 10.1073/pnas.96.18.10033 PMC17837 10468557

[21] Z.‐W. Luo , F.‐X.‐Z. Li , Y.‐W. Liu , S.‐S. Rao , H. Yin , J. Huang , C.‐Y. Chen , Y. Hu , Y. Zhang , Y.‐J. Tan , L.‐Q. Yuan , T.‐H. Chen , H.‐M. Liu , J. Cao , Z.‐Z. Liu , Z.‐X. Wang , H. Xie , Nanoscale 2019, 11, 20884. 31660556 10.1039/c9nr02791b

[22] X. Wu , W. Guo , L. Wang , Y. Xu , Z. Wang , Y. Yang , L. Yu , J. Huang , Y. Li , H. Zhang , Y. Wu , G. Li , W. Huang , Adv. Funct. Mater. 2021, 32, 2110066.

[23] S. Chen , M. L. Tomov , L. Ning , C. J. Gil , B. Hwang , H. Bauser‐Heaton , H. Chen , V. Serpooshan , Adv. Biol. 2023, 7, 2300124. 10.1002/adbi.202300124 37132122

[24] Y. Liang , X. Zhao , T. Hu , B. Chen , Z. Yin , P. X. Ma , B. Guo , Small 2019, 15, 1900046. 10.1002/smll.201900046 30786150

[25] a) G. M. Saed , W. Zhang , M. P. Diamond , Fertil. Steril. 2001, 75, 763; 11287032 10.1016/s0015-0282(00)01799-4

[26] X. Z. Shu , K. Ghosh , Y. Liu , F. S. Palumbo , Y. Luo , R. A. Clark , G. D. Prestwich , J. Biomed. Mater. Res. A. 2004, 68, 365. 14704979 10.1002/jbm.a.20002

[27] L. Wang , P. Chen , Y. Pan , Z. Wang , J. Xu , X. Wu , Q. Yang , M. Long , S. Liu , W. Huang , C. Ou , Y. Wu , Sci. Adv. 2023, 9, adh1753. 10.1126/sciadv.adh1753 PMC10403204 37540739

[28] a) W. Pan , P. Xin , S. Patrick , S. Dean , C. Keating , G. Clawson , J. Vis. Exp. 2010; 10.3791/2039 PMC3156072 20689511

[29] Y. Hua , K. Wang , Y. Huo , Y. Zhuang , Y. Wang , W. Fang , Y. Sun , G. Zhou , Q. Fu , W. Cui , K. Zhang , Nat. Commun. 2023, 14, 7632. 37993447 10.1038/s41467-023-43421-w PMC10665446

[30] J. Wang , Y. Chen , G. Zhou , Y. Chen , C. Mao , M. Yang , ACS Appl. Mater. Interfaces 2019, 11, 34736. 31518114 10.1021/acsami.9b12643

[31] S. Cerdido , M. Abrisqueta , J. Sánchez‐Beltrán , A. Lambertos , M. Castejón‐Grin , C. Muñoz , C. Olivares , J. C. García‐Borrón , C. Jiménez‐Cervantes , C. Herraiz , Cancer Lett 2024, 581, 216484. 38008393 10.1016/j.canlet.2023.216484

[32] R. Rao , Ann. N. Y. Acad. Sci. 2009, 1165, 62. 19538289 10.1111/j.1749-6632.2009.04054.x PMC6202026

[33] T. Xu , J. Hu , C. Fang , T. Luo , J. Liu , K. Zhang , ACS Mater. Lett. 2023, 5, 1892.

[34] M. Yang , Y. Zhang , C. Fang , L. Song , Y. Wang , L. Lu , R. Yang , Z. Bu , X. Liang , K. Zhang , Q. Fu , Adv. Mater. 2022, 34, 2109522. 10.1002/adma.202109522 35120266

