# Supplementary material for: Integrated ‘Shield‐Spear’ Biological Patch for Fibrosis‐Free Bladder Reconstruction
Source: Adv Sci (Weinh). 2025 Aug 13;12(41):e03975. doi: 10.1002/advs.202503975 (PMC12591207; doi:10.1002/advs.202503975)
Supplement: Supplementary file 1 — Supporting Information [file ADVS-12-e03975-s003.docx]

**Integrated ‘Shield-Spear’ Biological Patch for Fibrosis-Free Bladder Reconstruction**

Xiaoqi Wu^1#^, Huitong Ruan^2#,*^, Xiaolin Zhang^1#^，Weihan Zheng^3#^, Muhetaierjiang Mutailifu^2^, Ling Wang^3^, Liu Yu^3^, Ruijun Peng^3^, Rui Zhao^3^, Zihan Wang^3^, Jie Xu^3^, Shaochuan Li^4^, Yaobin Wu^3*^, Wenguo Cui^2*^, Mujun Lu^1*^

X. Wu, X. Zhang, M. Lu

*^1^* *Department of Urology and Andrology, Ren Ji Hospital, School of Medicine, Shanghai Jiao Tong University, Shanghai 200001, China*

*E-mail:* [*lumujun@163.com*](mailto:lumujun@163.com)

*H. Ruan, M.* *Mutailifu, W. Cui*

*^2^ Department of Orthopaedics, Shanghai Key Laboratory for Prevention and Treatment of Bone and Joint Diseases, Shanghai Institute of Traumatology and Orthopaedics, Ruijin Hospital, Shanghai Jiao Tong University School of Medicine, 197 Ruijin 2nd Road, Shanghai 200025, China.*

*E-mail:* [*wgcui80@hotmail.com*](mailto:wgcui80@hotmail.com)*;* [*/ruanhuitong@sjtu.edu.cn*](mailto:/ruanhuitong@sjtu.edu.cn)

*W. Zheng, L. Wang, L. Yu, R. Peng, R. Zhao, Z, Wang, J. Xu, Y. Wu*

*^3^ Guangdong Engineering Research Center for Translation of Medical 3D Printing Application, Guangdong Provincial Key Laboratory of Medical Biomechanics, Department of Human Anatomy, School of Basic Medical Sciences, Southern Medical University, Guangzhou, 510515, China*

*E-mail:* [*wuyaobin2018@smu.edu.cn*](mailto:wuyaobin2018@smu.edu.cn)

*S, Li*

*^4^ South China Agricultural University, Guangzhou, 510642, China*

### *Methods*

The Medical Sodium Hyaluronate Gel (HA, 20173644735) was provided by Hangzhou Xiehe Medical Supplies Company located in Hangzhou, China. Sigma-Aldrich (Shanghai, China) supplied Ethyl(dimethylaminopropyl) carbodiimide (EDC), N-hydroxysuccinimide (NHS), 2-aminoethyl methacrylate hydrochloride (AEMA), and dopamine hydrochloride. Cell Membrane Staining Kit (PKH67) purchased from bestbio biotechnology (Shanghai, China). Dulbecco's Modified Eagle's Medium (DMEM), Dulbecco’s phosphate-buffered saline (DPBS), fetal bovine serum (FBS), and the Live/Dead Viability Assay Kit were purchased from Invitrogen (Life technologies).

### *Synthesis and characterization of HAD polymer*

The synthesis of HAD polymers was conducted following our previous findings. In brief, 1 g of HA was dissolved in ddH_2_O to obtain a concentration of 1% w/v. To this HA solution, EDC (4 mmol), NHS (4 mmol), and AEMA (3 mmol) were added. Subsequently, dopamine hydrochloride (DA, 3 mmol) was introduced and the solution was stirred for 24 hours. After the reaction, the monomer was dialyzed under acidic conditions for 3 days. The dialyzed HAD polymer was then lyophilized and stored in a light- and moisture-free environment. It is important to keep the polymer away from light and moisture. As a control, HAMA, which is synthesized by grafting AEMA groups onto HA polymers, was used without the presence of DA groups. The chemical structures of HAD and HAMA were analyzed using Fourier transform infrared (FT-IR). The FT-IR spectra (NICOLET 6700, Thermo) were recorded in the range of 1500–3500 cm^-1^.The degree of substitution (DS) of AEMA to the backbone of HA was calculated based on the areas under the peaks at 5.6–6.3 ppm (Aa) and 2.0 ppm (Ab). The degree of modification of DA to the backbone of HA was calculated based on the areas under the peaks at 6.6–7.4 ppm (Ad) and 2.0 ppm (Ab), using the following equations:

DS_AEMA-HA_ = (Aa ∕ Ab) × (3 ∕ 2) × 100% (1)

DS_DA-HA_ = (Ad ∕ Ab) × (3 ∕ 3) × 100% (2)

### *Mechanical characterization of HAD hydrogels*

A 3 wt% HAD solution containing 0.1 wt% LAP was poured into cylindrical molds (1 mm height × 20 mm diameter) to form photocrosslinked HAD hydrogel samples for rheological testing. The tests were conducted using a rheometer (HAAKE MARS 40, Thermo Scientific) with a parallel-plate geometry of 20 mm in diameter. Initially, a frequency sweep from 0.1 to 10 Hz was performed under a 1% strain to measure the storage modulus (G') and loss modulus (G") of the hydrogels. Subsequently, strain sweep oscillatory tests were carried out with shear strains ranging from 1% to 100% at a constant frequency of 1 Hz. All tests were performed at room temperature in a humidity-controlled environment. Additionally, steady shear viscosity measurements of the HAD precursor were conducted, with shear rates varying from 0.1 to 10 s⁻¹. Each test was repeated in triplicate to ensure accuracy.

### *Adhesion capacity of HAD hydrogels on bladder tissue surfaces*

The bursting pressure assay was carried out to quantitatively access the adhesion strength of hydrogels according to the our previously reported method^1^. The lap shear adhesion test of HAD was performed according to our previous study^1,2^. To summarize the "gel-nonadhesive" process, 300 μL of the HAD precursor containing 0.1 wt% LAP was evenly spread on fresh porcine skin substrate. The HAD precursor was then crosslinked under UV irradiation (365 nm) for 60 seconds. Once the HAD hydrogel was formed, its inner surface adhered to the porcine skin, while the outer surface remained nonadherent. Subsequently, another piece of fresh porcine skin was placed onto the outer surface of the HAD hydrogel, and the adhesion properties were tested and recorded using the Materials Test system (MTS Criterion 43, MTS Criterion) equipped with a 50N load cell at a rate of 10 mm/min. All these tests were employed 3 times.

### *Standard peel test to quantify adhesion energy of HAD*

An L-shaped support structure was fabricated using 3D printing, with dimensions of 1 cm in width, 4 cm in length, and 0.3 cm in thickness. Fresh porcine skin was cut into two rectangular strips measuring 1 cm × 4 cm. One piece of porcine skin was adhered to the inner surface of the L-shaped support using cyanoacrylate adhesive. Subsequently, 3% HAD or fibrin glue was applied to half of the outer surface area (1 cm × 2 cm). The outer surface of the second piece of porcine skin was then overlapped onto the region coated with HAD or fibrin glue. The adhesion between the two pieces of skin was cured using 365 nm ultraviolet light for 1 minute.

For the tensile testing, the porcine skin and the L-shaped support were clamped and subjected to stretching (see Supporting Information Figure S6c). The stretching rate was set at 10 mm/min, with a sensor capacity of 50 N, using an Materials Test system. All these tests were employed 3 times.

### *Extracellular vesicles isolation and identification*

Human ASCs-EVs were isolated following the methods described in a previous study^3^. Informed written consent was obtained from the donors prior to the collection of samples. Here's a brief overview of the isolation process: First, human ASCs were cultured using exosome-free fetal bovine serum (FBS). Details of the human ASCs culture can be found in the Supporting Information. Serum-free conditioned medium from human ASCs was collected and underwent a series of centrifugation steps for cell and debris removal. The medium was centrifuged at 300 g for 10 minutes at 4°C, followed by centrifugation at 16,500 g for 10 minutes at 4°C. The supernatant was then subjected to ultracentrifugation at 120,000 g for 70 minutes at 4°C using an Optima L-100 XP ultracentrifuge with an SW 32 ti rotor (κ-factor:204, Beckman Coulter Inc., CA, USA). The pelleted human ASCs-EVs were treated with a BCA Assay Kit based on the manufacturer's instructions (Beyotime Biotechnology, Shanghai, China) to determine the total protein content. The protein contents were measured using a spectrophotometer (Varioskan, Thermo Fisher Scientific Inc., MA, USA) at an absorbance of 562 nm, and the values were extrapolated against a standard curve.

Furthermore, the human ASCs-EVs were characterized using various techniques including transmission electron microscopy (TEM), nanoparticle tracking analysis, and western blotting. To visualise the EVs, PKH-67 was used to label the EVs according to the manufacturer's instructions (Sigma-Aldrich).

### *Preparation of S100Apt-EVs*

The structure of the aptamer was reported in a previous study^4,5^. The 5'-end of the aptamer was modified with an aldehyde group, which could react with amino group-containing molecules on EVs through a Schiff base reaction^5^. Briefly, 200 nM of the aldehyde-modified aptamers (obtained from Sangon Biotech) were added to 1.0 mg/mL EVs in PBS. The mixture was allowed to react in a rotating mixer at 4°C overnight. To remove any unconjugated aldehyde-aptamer, the solution was subjected to three washing steps using 100 kDa ultrafiltration tubes. The resulting aptamer-conjugated EVs (referred to as S100Apt-EVs) were then available for specific linkage to Schwann cells through high-affinity recognition.

### *Uptake assessments of S100Apt-EVs*

Internalization assays were conducted to evaluate the cellular uptake efficacy of the aptamer in promoting EVs. Cy5-labeled Apt-CHO was employed for this purpose, while Cy5-labeled aldehyde-modified random sequence (Rd) was used as a negative control. The EVs were labeled with PKH67. The groups were then mixed and cultured with Schwann cells. After a 12-hour incubation period, fluorescence microscopy was performed to visualize the internalized EVs, and the fluorescence intensity was subsequently quantified.

***S100Apt-EVs promote neuroprotection and increase the length and number of branching Schwann cells***

The expression level of BDNF protein was determined through Western blotting analysis. RSC96 cellswere co-incubated with different samples, including 200 μg/mL EVs, S100Apt-EVs, S100Apt-EVs treated with proteinase K (50 μg/mL), and a control group. After 24 hours of co-incubation, the cells were lysed to extract the proteins. The protein samples were then subjected to electrophoresis, transmodelling, and exposure to obtain protein bands. The protein bands were quantified using image analysis software Image J. Similarly, the expression levels of NT-3 and NGF proteins were verified using a similar approach. Fluorescence microscopy was conducted to investigate the impact of various coculture treatment on the formation of Schwann cell branch projections.

### *Controlled released of S100Apt-EVs from HAD-AptEV hydrogel*

To generate HAD-AptEV hydrogel at a final concentration of 3% HAD and 1mg/mL S100Apt-EVs, the S100Apt-EVs were mixed into the HAD precursor solution and crosslinked. In order to visualize the distribution of S100Apt-EVs within the hydrogel, the S100Apt-EVs were pre-stained with PKH26 (red), while the HAD precursor solution was loaded with FITC (green). Confocal microscopy was used to observe the HAD and HAD-AptEV hydrogels. The release profile of EVs in vitro was assessed using a BCA protein assay kit (Thermo Scientific Pierce, China). Initially, a HAD-AptEV hydrogel containing S100Apt-EVs (1mg/mL) was photocrosslinked for 10 s using a 365 nm UV-light. The hydrogel was then placed in 24-well plates (n = 5), with 200 µL of PBS (pH = 7.4) added to each well. The HAMA+EVs group was prepared using the same method. At each time point (day 1, 3, 5, 7, 9, 11, 13, 15,28 and 35) with 5 repetition groups, 100 µL of the supernatant was collected and replaced with 100 µL of fresh PBS. The content of released exosomes was measured, and the percentage of exosomes released was calculated. In a rat bladder defect model, the release of 1 mL of HAD-AptEV (AptEV at a concentration of 1 mg/mL) and 1 mL of HAMA+EVs (EVs at a concentration of 1 mg/mL) was observed using small animal in vivo imaging on days 0, 14, 28 and 35.

### *Hemicastration in rat bladder defect models*

Animal experiments were approved by the Ethics Committee of Renji Hospital Affiliated to Shanghai Jiao Tong University School of Medicine, with approval number KY2022-180-B. Eight-week-old Sprague-Dawley (SD) rats (Shanghai Sippr BK Laboratory Animals Ltd., Shanghai, China) with matched body weights were acclimated for 1 week. They were labeled with numbers and randomly divided into four groups: HAD-AptEV group (treatment of bladder defects with HAD-S100AptEVs, n = 6), HAD group (treatment of bladder defects with HAD, n = 6), and the suture group (closure of bladder defects using sutures, n = 6). The allocation of rats to the groups was determined using a random number table, and blinding was ensured to eliminate bias. The regeneration of bladder morphology was assessed through retrograde cystography, gross examination of the bladder, gross longitudinal section of the bladder.

Process: After anesthetizing the rat, a 2 cm × 2 cm defect was manually created at the apex of the bladder. Hemostasis was immediately achieved using gauze to absorb both blood and urine. Once the defect area was sufficiently dry, a 3 wt % HAD precursor solution, noted for its high viscosity and limited flowability based on prior shear analysis data, was injected around the edges of the defect. UV curing at 365 nm was applied to polymerize the precursor at the defect’s perimeter. Subsequently, the precursor solution was gradually injected from the outer edges towards the center, with simultaneous curing, until the entire defect was sealed. After sealing, additional HAD precursor solution was applied to the interface between the gel and the bladder defect to reinforce the closure. In areas where bleeding or urine leakage persisted, further precursor solution was applied and cured to ensure a secure seal of the HAD gel at the defect site.

***Bladder reconstruction of the integrated ‘Shield-spear’ HAD-AptEV Patch in beagle models***

All animal procedures were performed in accordance with the guidelines for Care and Use of Laboratory Animals of Shanghai Jiao Tong University Agriculture and Biology Experimental Internship Field Co., Ltd. The assigned approval number is JDLL20240106. Twelve healthy female beagles, weighing between 13 to 16 kg, were randomly divided into four groups (n=3 per group): Normal group, Suture group, HAD group, and HAD-AptEV group. The purpose of this study was to assess the efficacy of the integrated ‘Shield-Spear’ HAD-AptEV Patch for bladder reconstruction in beagle models. The regeneration of bladder morphology was assessed through retrograde cystography, gross examination of the bladder, gross longitudinal section of the bladder, bladder regenerated area, hydrogel degradation, fibrosis, and inflammation.

Process: Following anesthesia administration to the beagle, a 2 cm × 2 cm defect was manually created at the apex of the bladder. Hemostasis was immediately achieved using gauze to absorb blood and exuding urine. Once the defect site was sufficiently dry, a 3 wt% HAD precursor solution was injected around the perimeter of the defect. The solution, characterized by high viscosity and poor flowability as reported in previous shear analysis studies, was carefully applied. UV curing at 365 nm was employed to polymerize the precursor at the edges of the defect. Subsequently, the precursor solution was incrementally injected from the periphery towards the center of the defect, with concurrent curing to ensure gradual and complete sealing of the defect. Once the defect was sealed, additional HAD precursor solution was applied at the gel-tissue interface to reinforce the seal. In areas with persistent bleeding or urine leakage, further applications of the precursor solution were performed, followed by UV curing to ensure a secure seal of the HAD gel over the defect site. The Sture group was treated with surgical sutures.

Alternatives：After using gauze to stop the bleeding and absorb the exuding urine from the bladder and defect, we used tweezers to align the smooth muscle layers on both sides of the defect. We then applied the 3wt% HAD precursor solution to the defect and cured it with 365nm UV light.

### *Histology*

After 4 weeks, beagle dogs were euthanized, and the bladders were harvested for histological and immunohistochemical analysis. Tissues were fixed in 10% formalin, embedded in paraffin, and sectioned. Masson staining was performed to evaluate tissue regeneration and integration of the patch. For immunofluorescence staining, bladder sections were incubated with anti-Collagen Type I (COL1)rabbit Polyclonal antibody (1:500, Proteintech) , anti-smooth muscle actin（αSMA）rabbit Polyclonal antibody (1:3000, Proteintech), and Anti-GATA6 rabbit Polyclonal antibody(1:400 Bioss). Subsequently, the sections were incubated with goat anti-rabbit Alexa Fluor Cy3-conjugated secondary antibody (1:500, Abcam). Finally, the nuclei were stained with DAPI solution (Beyotime) and the sections were observed under a fluorescence microscope (Olympus).

***Gene ontology (GO) and KEGG enrichment analysis***

Gene Ontology (GO; http://www.geneontology.org) represents a comprehensive framework aimed at the annotation of genes and proteins according to their involvement in biological processes, molecular functions, and cellular components. Meanwhile, The Kyoto Encyclopedia of Genes and Genomes (KEGG; http://www.genome.jp/kegg/) serves as an online repository that catalogs the biological pathways associated with genes and biochemical substances. To facilitate the annotation of enriched GO terms and KEGG pathways, the R package clusterProfiler was employed.

### *Statistical analysis*

The experiments were performed in triplicate for each sample (n ≥ 3), ensuring the robustness and reliability of the results. The experimental data obtained were analyzed using a one-way analysis, followed by Tukey’s significant difference post hoc test for further statistical analysis and comparison.To ensure representative results, at least 6 images were captured and analyzed, which were derived from 3 independent replications of the samples.

**Table S1.** Comprehensive properties of recently used biomaterials for bladder defect repair.

| **Biomaterials** | **Wet-adhesiveness**  **(kPa)** | **Suturing** | **In-situ Curing Time (min)** | **Oxidation agent** | **Anti-stone or adhesion** | **Inhibition of oxidative stress** | **Compliance of bladder (μl/cmH2O)** | **X=**  **Bladder defect repair efficiency** | **Y=**  **Neuroregeneration** |
| --- | --- | --- | --- | --- | --- | --- | --- | --- | --- |
| **BAMG-HS** | **< 5** | **√** | **> 5** | **×** | **×** | **×** | **> 15** | **1** | **1** |
| **BAMG-SF** | **< 5** | **√** | **< 5** | **×** | **×** | **×** | **> 15** | **2** | **1** |
| **BAMG-HS-EVs** | **< 5** | **√** | **< 5** | **×** | **√** | **√** | **> 15** | **3** | **2** |
| **ATE/HA-PLCL** | **< 5** | **√** | **> 5** | **×** | **√** | **×** | **> 30** | **1** | **2** |
| **HA-PLCL** | **< 5** | **√** | **> 5** | **×** | **×** | **×** | **> 15** | **1** | **1** |
| **AM-SF** | **< 5** | **√** | **> 5** | **×** | **×** | **√** | **> 30** | **1** | **2** |
| **Chitosan scaffold** | **> 5** | **√** | **> 5** | **×** | **√** | **√** | **> 30** | **2** | **3** |
| **BAMG-ASCs** | **< 5** | **√** | **> 5** | **×** | **×** | **×** | **< 15** | **1** | **0** |
| **Fibrin glue** | **< 5** | **×** | **> 5** | **×** | **×** | **×** | **< 15** | **1** | **0** |
| **HAD-S100Apt-EVs**  **(This study)** | **> 12** | **×** | **< 5** | **×** | **√** | **√** | **> 20** | **4** | **3** |

- If the wet-adhesiveness is less than 5kPa, the score of hydrogels was 0, otherwise it is 1;
- If the hydrogel needs suturing treatment, the score of hydrogels was 0, otherwise it is 1;
- If the *in situ* curing time is less than 5 minutes, the score of hydrogels was 1, otherwise it is 0;
- If the gelation of hydrogels needs oxidation agent, the score of hydrogels was 0, otherwise it is 1;
- If the hydrogel exhibits the efficiency of anti-stone or adhesion, the score is 1, otherwise it is 0;
- If the hydrogel can inhibit oxidation during the healing process, the score of hydrogels was 1, otherwise it is 0;
- If the compliance of bladder is over 15 μl/cmH_2_O after the hydrogel treatment, the score of hydrogels was 1, otherwise it is 0 ;
- X: Bladder repair efficiency=the sum score of "wet-adhesiveness", "Suturing", "*in situ* Curing time" and “Oxidation agent”.
- Y: Antifibrotic efficiency = the sum score of "Anti-stone or adhesion", "Inhibition of oxidative stress" and “Compliance of bladder”.


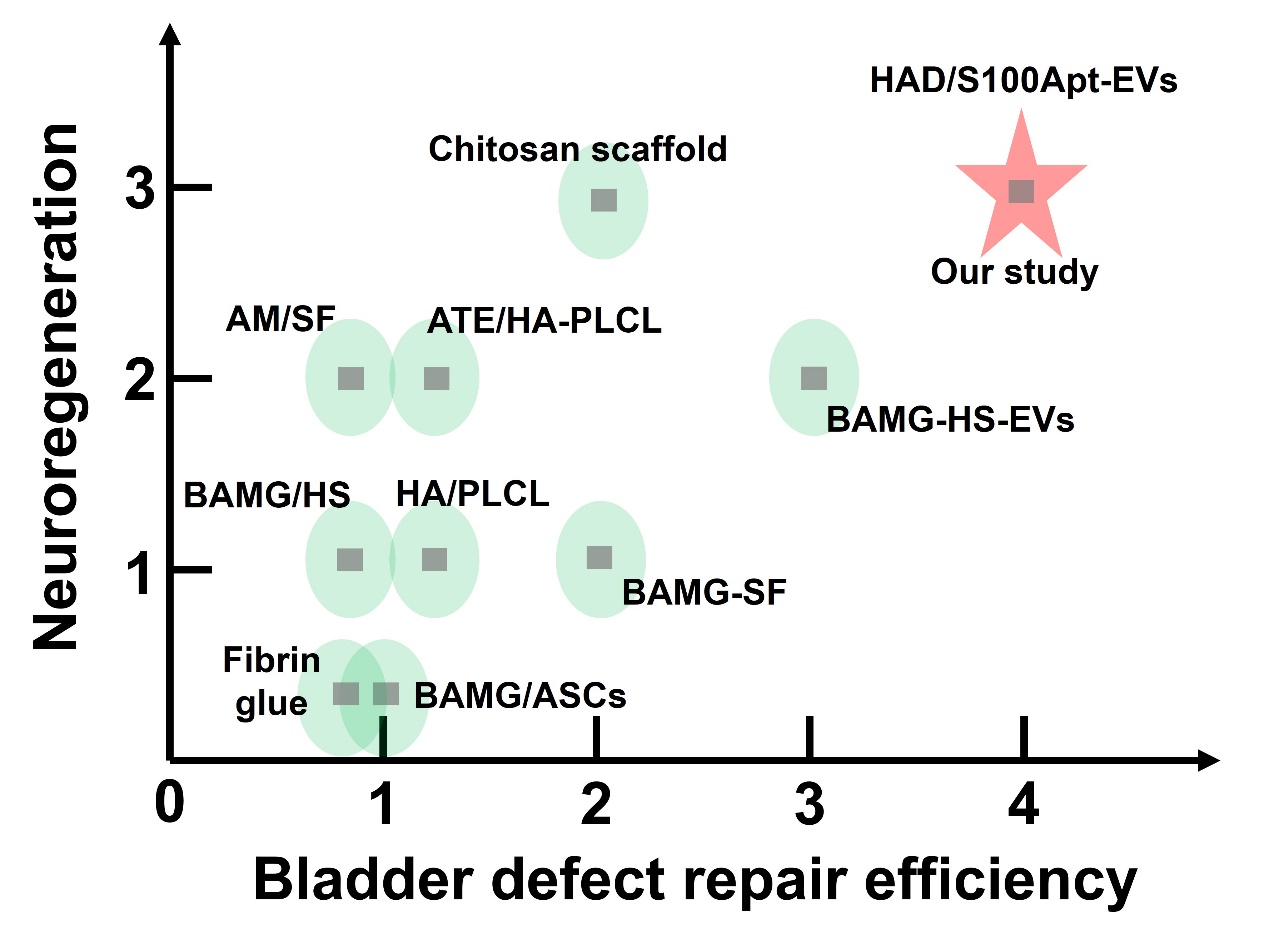


**Figure S1** Comparison of this work with other studies. The optimal HAD hydrogel

combines tissue adhesion, antifibrotic efficiency and sutureless repair efficiency. The scatter plot qualitatively compares the HAD hydrogel with those reported in the literature^3,6-12^.

.

**
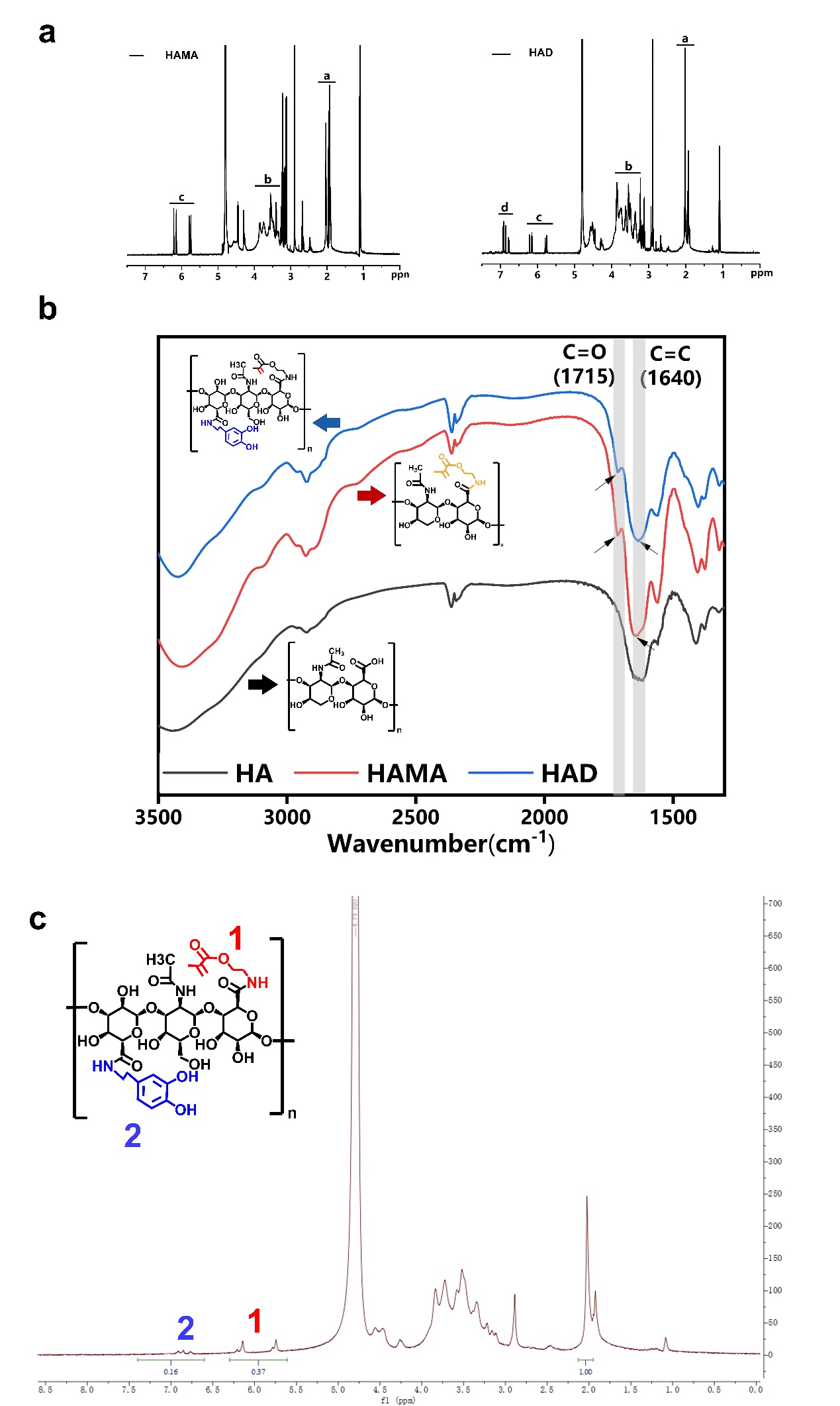
**

**Figure S2** a) ^1^HNMR spectra of HAD formulations. a labeled the C(=O)CH3 in HA (δ =2.1 ppm); b) labeled protons in the ring structures of HA (δ =4.0 ~3.0 ppm); c) labeled the C=Cof 2-aminoethyl methacrylate hydrochloride (AEMA) (δ=5.68 and 6.13 ppm); d) labeled protons in the catechol ring of dopamine(DA) (δ=6.5~7.2 ppm). b) For the FT-IR spectra of the HAD formulations, the appearance of absorption bands at 1715 cm^-1^ and 1640 cm^-1^ were assigned to the carbonyl group groups, vinyl groups, respectively. c) The degree of modiﬁcation of AEMA to the backbone of HA was calculated based on the areas under the peaks at 5.6–6.1 ppm (1). The degree of modiﬁcation of DA to the backbone of HA was calculated based on the areas under the peaks at 6.5–7.2 ppm (2).


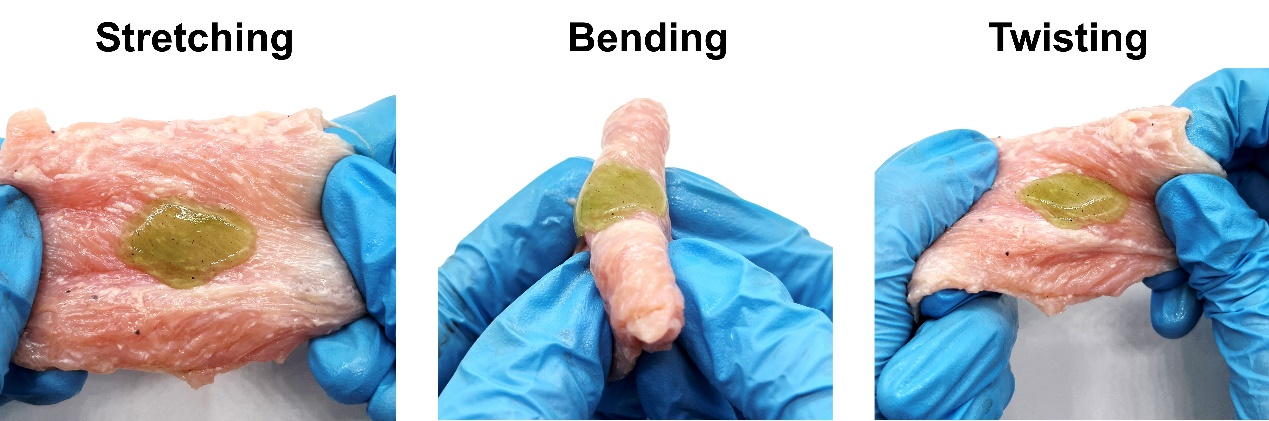


**Figure S3** Hydrogels demonstrated robust adherence to the ex vivo porcine bladder under torsion at various angles.


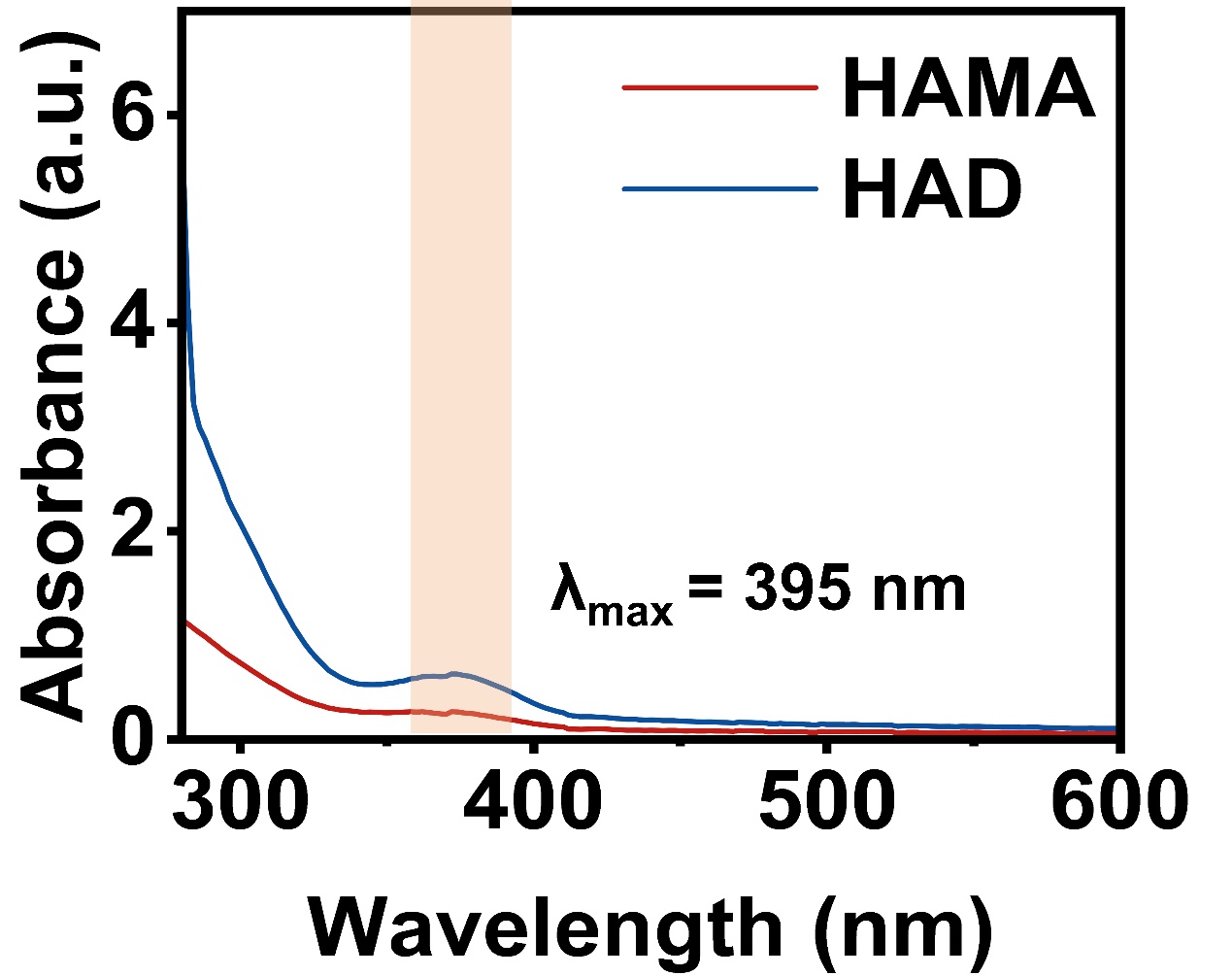


**Figure S4** UV–vis spectra of HAD hydrogel solutions after oxidation at 37 °C. Quinone peak: λ max (ε) = 395 nm


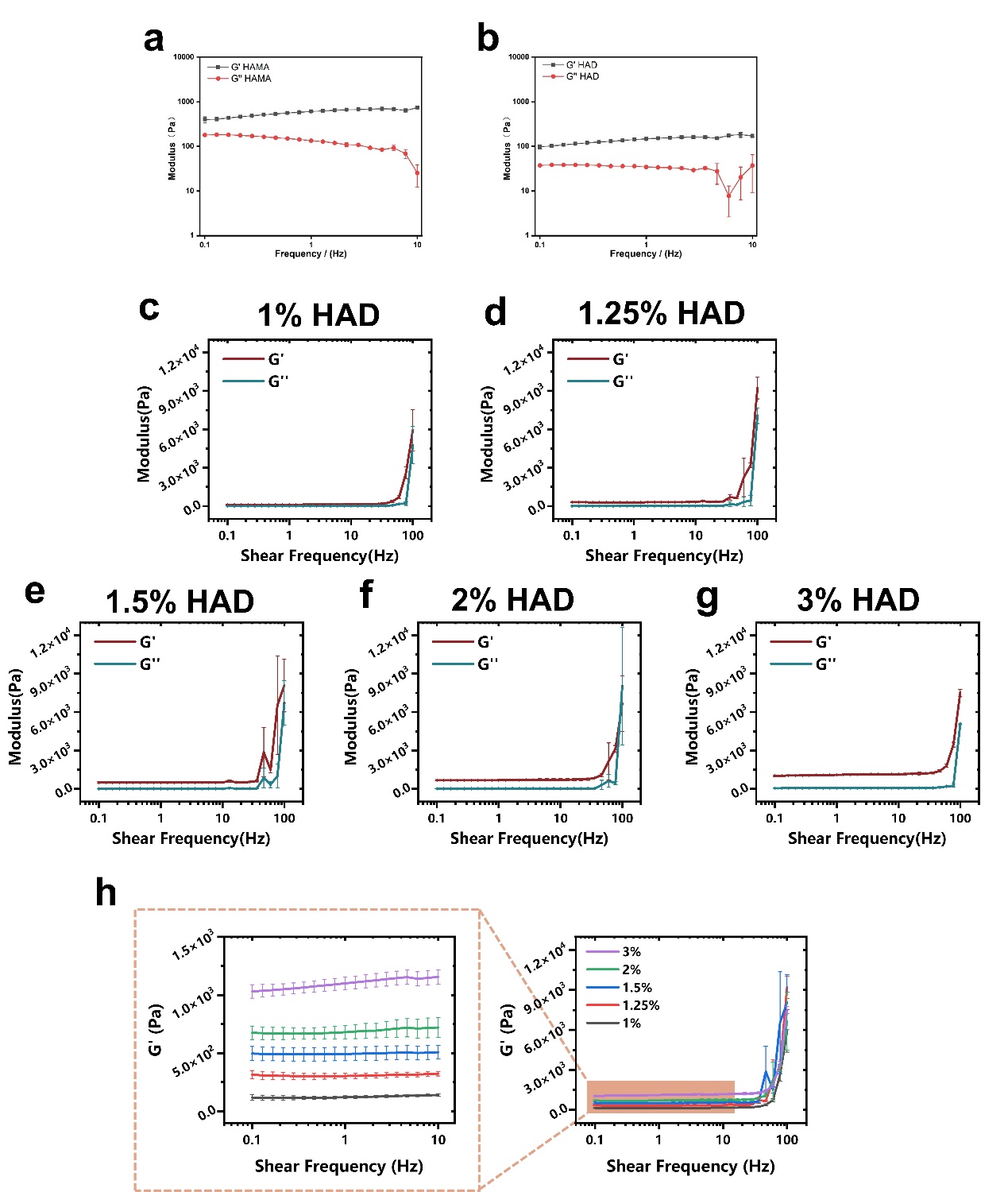


**Figure S5** Rheological properties of HAMA a) and HAD hydrogels b) with the frequency-sweep (0.1–10 Hz, strain of 1%) test. c-g): Rheological data of hydrogels with different HAD concentrations. h): The modulus of HAD hydrogels at different concentrations remained stable between 0.1-10 Hz.


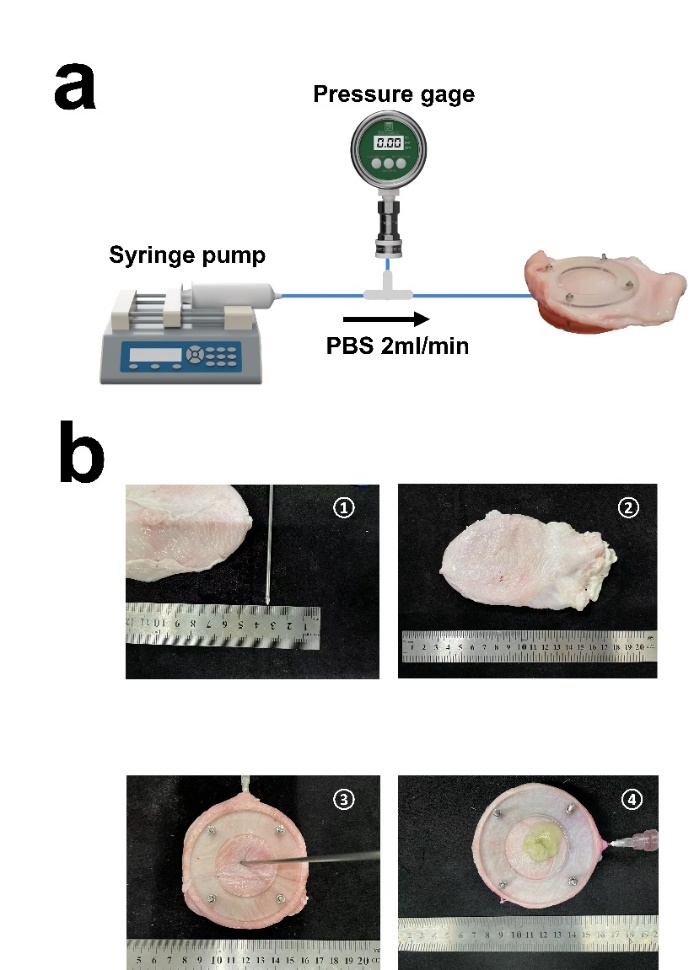


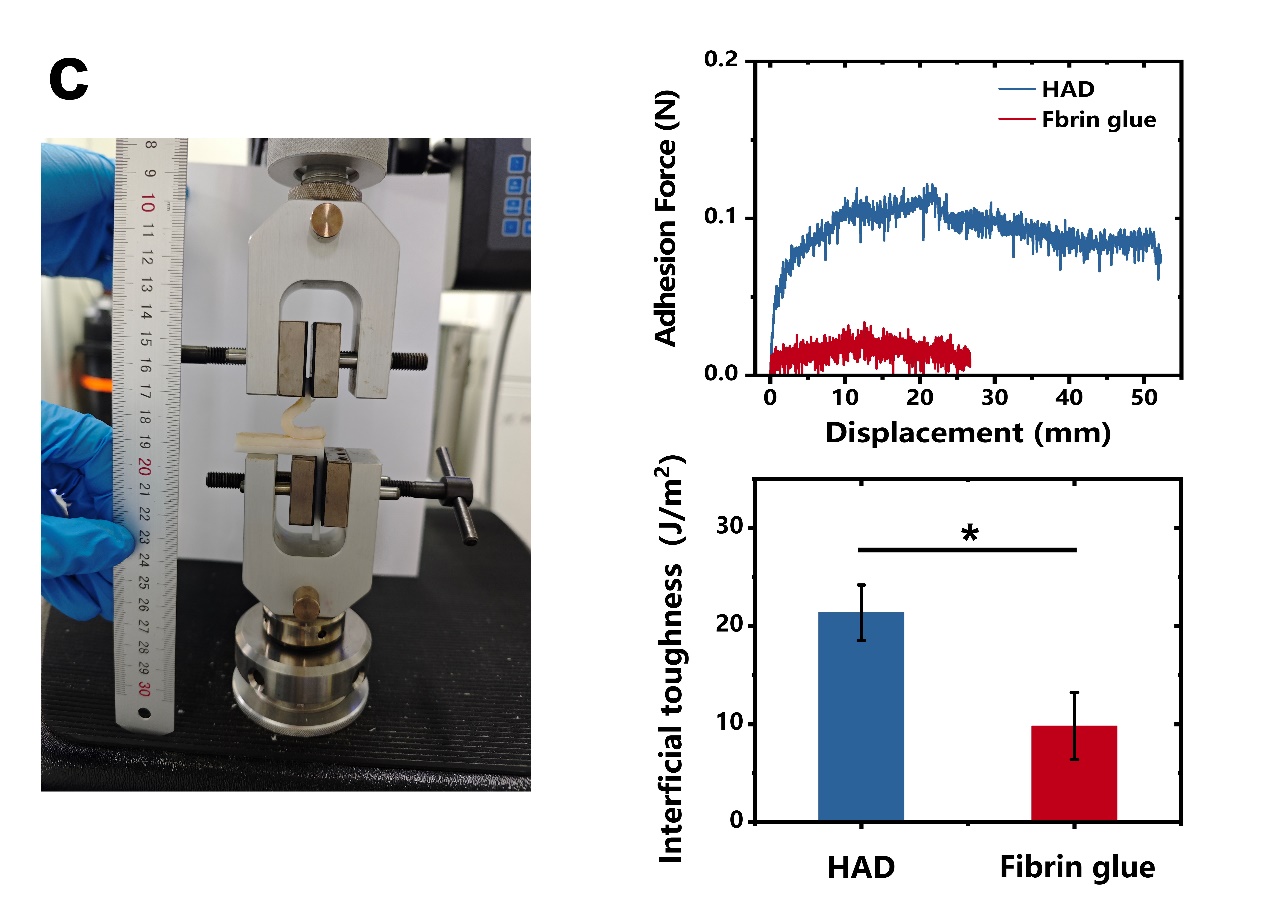


**Figure S6 Adhesive property of HAD hydrogel.** a) Schematic diagram of the bursting press test. b) The process of preparing the bursting adhesion test of HAD hydrogels on porcine bladder. c) Standard peel test to quantify adhesion energy of HAD.


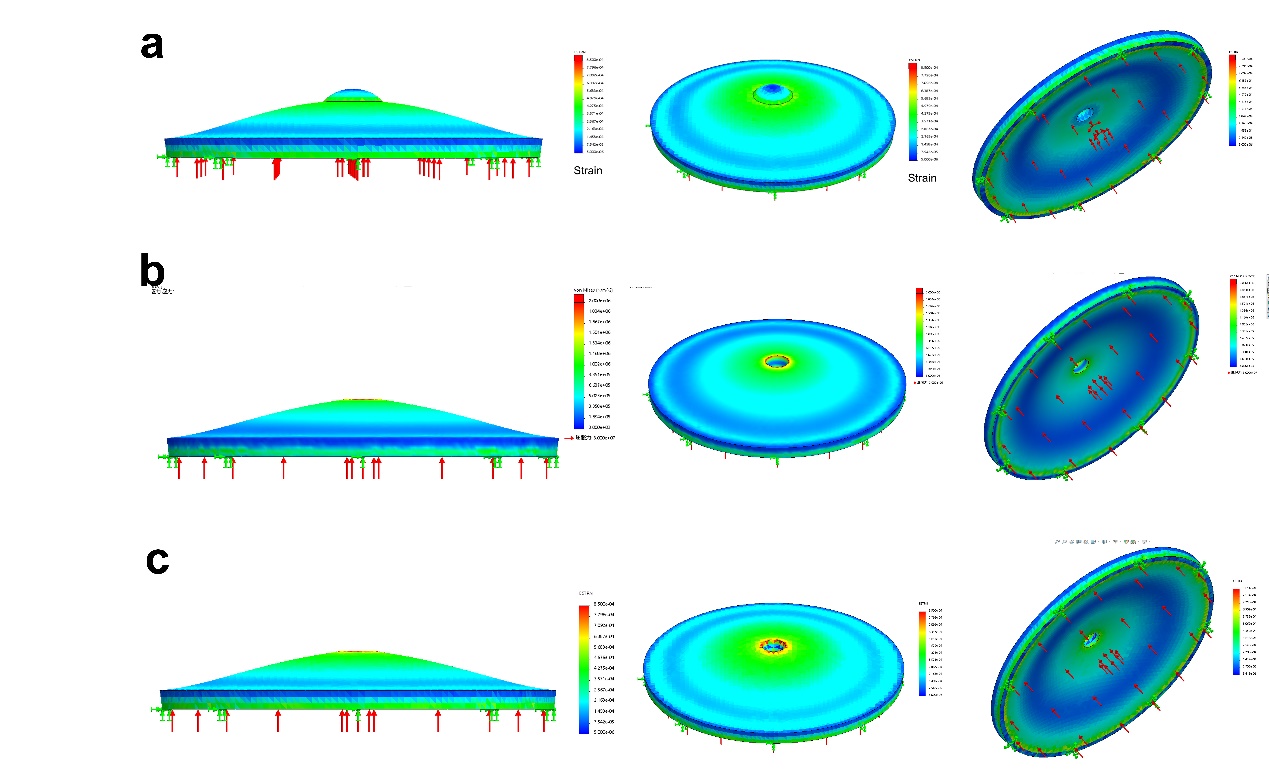


**Figure S7** Finite element analysis of HAD sealant plug for porcine bladder defects. a) Strain of HAD gel on porcine bladder. b) Stress in the bladder defect in the untreated group. c) Strain of bladder defect in untreated group. Finite element cloud map analysis of each group was obtained under the condition of water flow rate of 2ml/min.


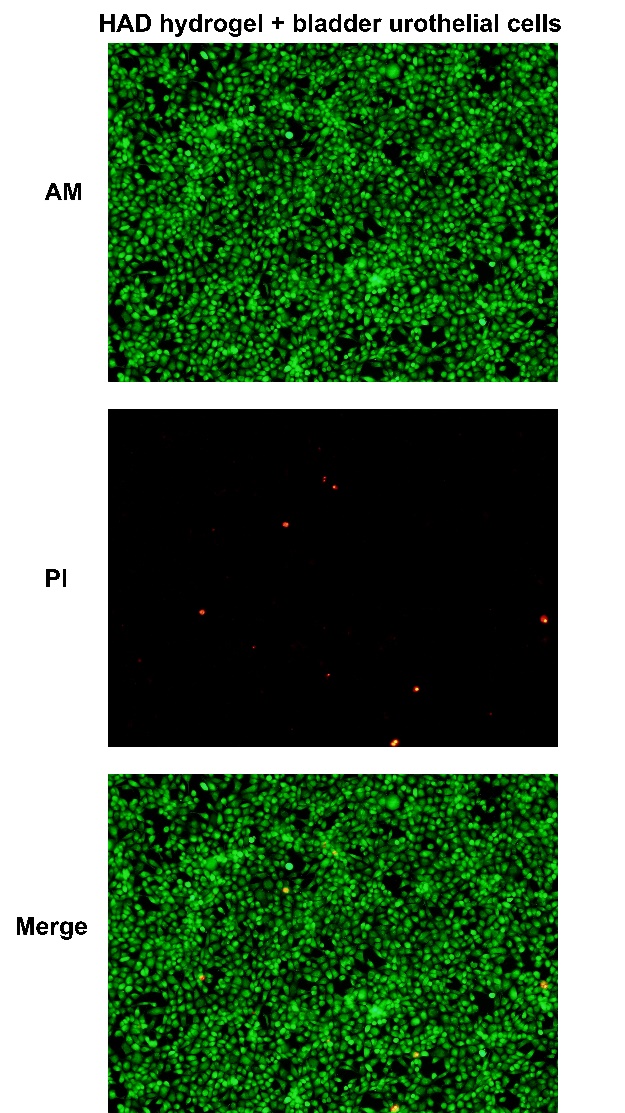


**a**

**
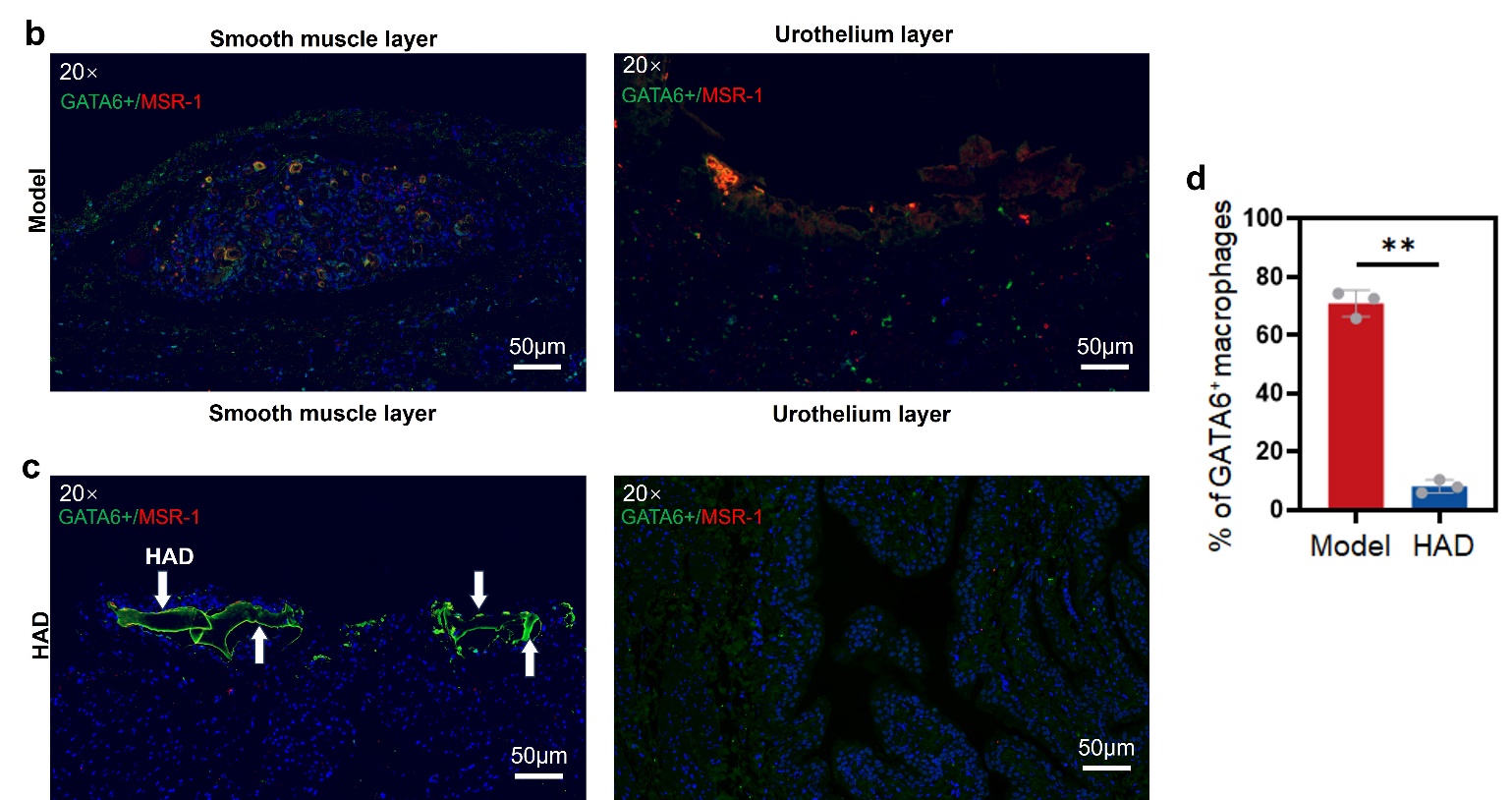
**

**Figure S8 a)** Fluorescence images of bladder epithelial cells stained with live/dead dyes after 24 h of culture on the HAD hydrogel. Green and red represent live and dead cells, respectively. Standard culture system:5% CO2 concentration, 10% fetal bovine serum, and DMEM basal medium. The control group used TCP. b) Aggregation of GATA6 macrophages in the SM and UE layers of the model group. c) Aggregation of GATA6 macrophages in the SM and UE layers of the HAD group. UE: urothelial layer. SM: smooth muscle layer. d) Quantitative analysis of GATA6 macrophages on the surface of HAD gel and wound sites in the Model group. The tissue samples were obtained from SD rat bladder tissue 4 weeks after surgery. Three areas were randomly selected at the wound site for quantitative analysis in each slice.


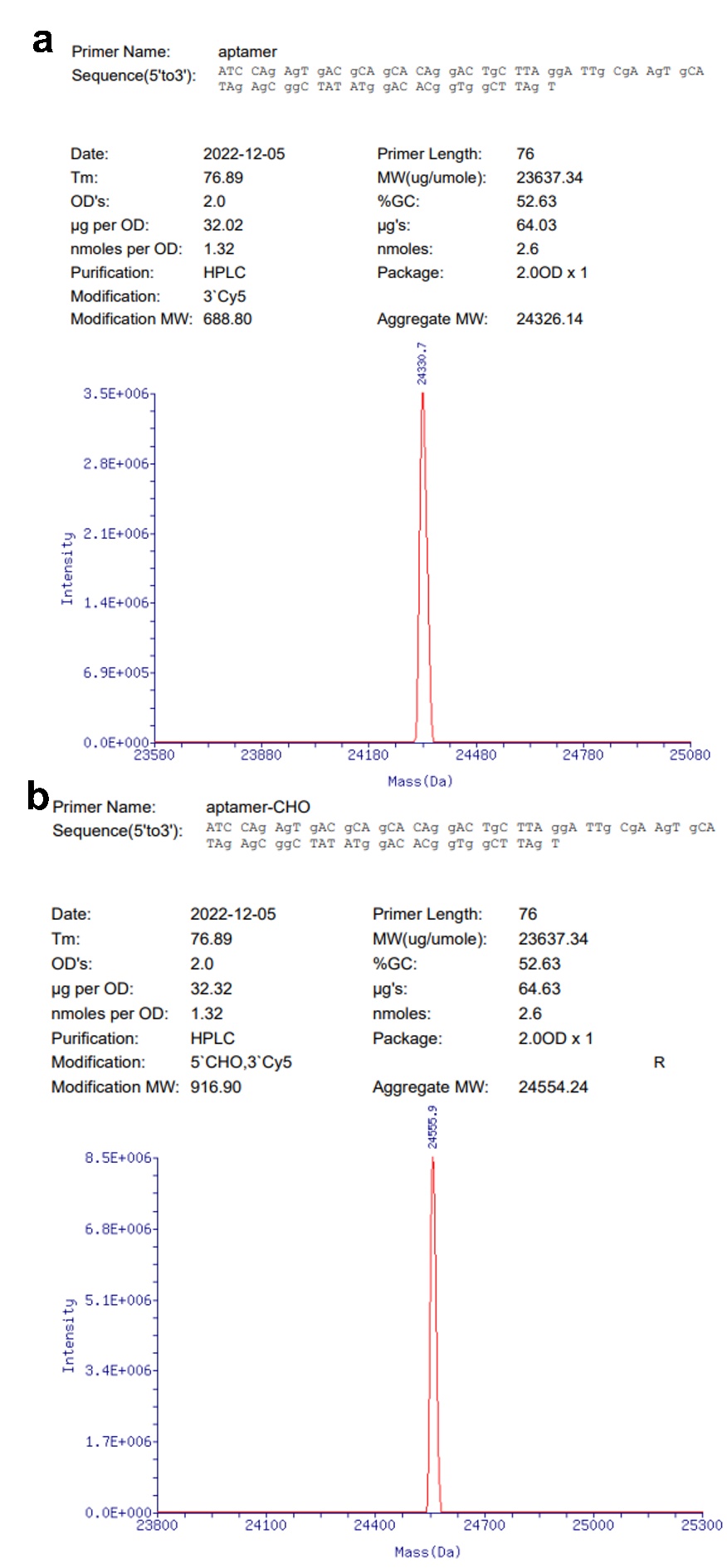


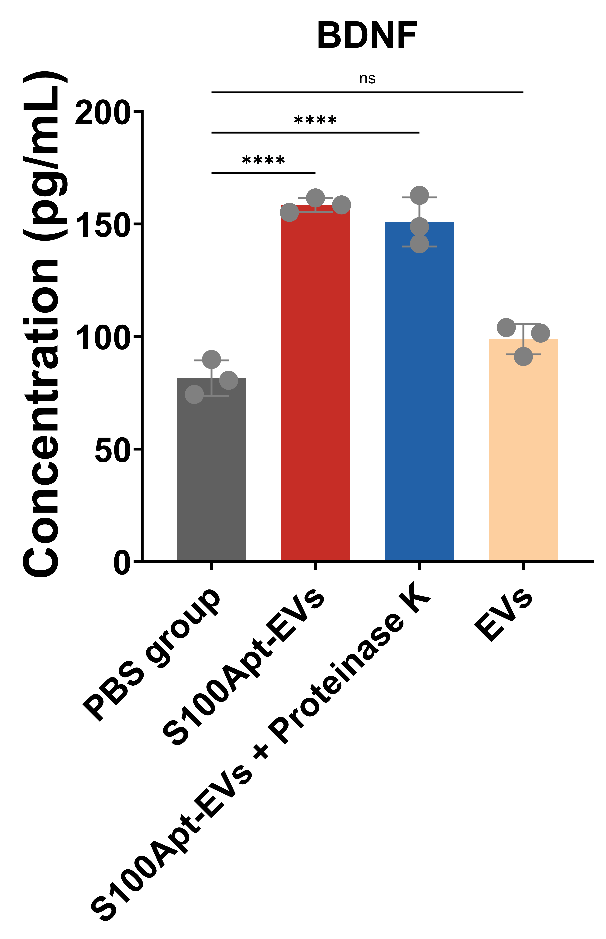


**c**

**Figure S9** a) DNA mass spectrometry analysis of S100Aptamer and b) S100Aptamer-CHO. c) ELISA was used to measure the amount of BDNF secreted in the culture supernatant of RSC96 cells in each group.


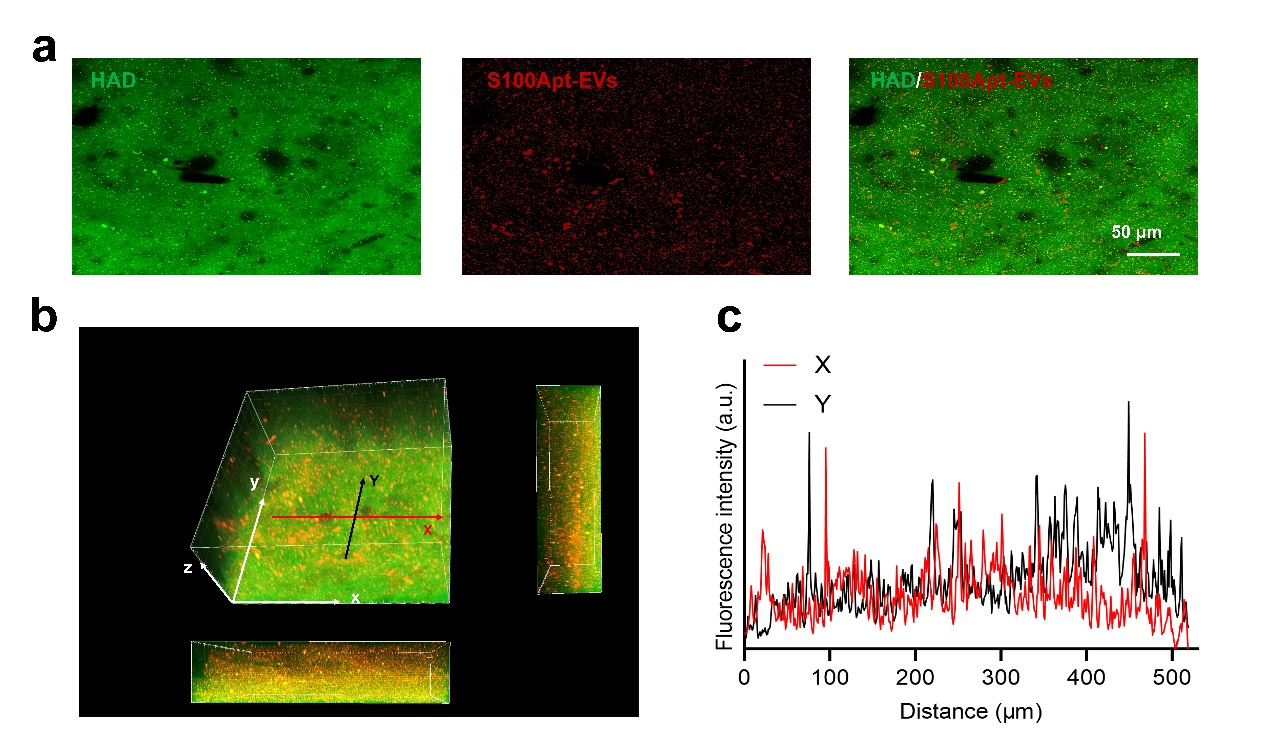


**Figure S10** Distribution of EVs in the hydrogel" and bolded the relevant text in the manuscript a) Fluorescence images showing ADSC-EVs (red; labeled with PKH26) encapsulated within HAD hydrogels (green; labeled with FITC). b) 3D views of confocal HAD+EVs hydrogels images and c) fluorescence intensity analysis of their cross sections revealing the homogeneous ADSC-EVs distribution within HAD+EVs hydrogel.


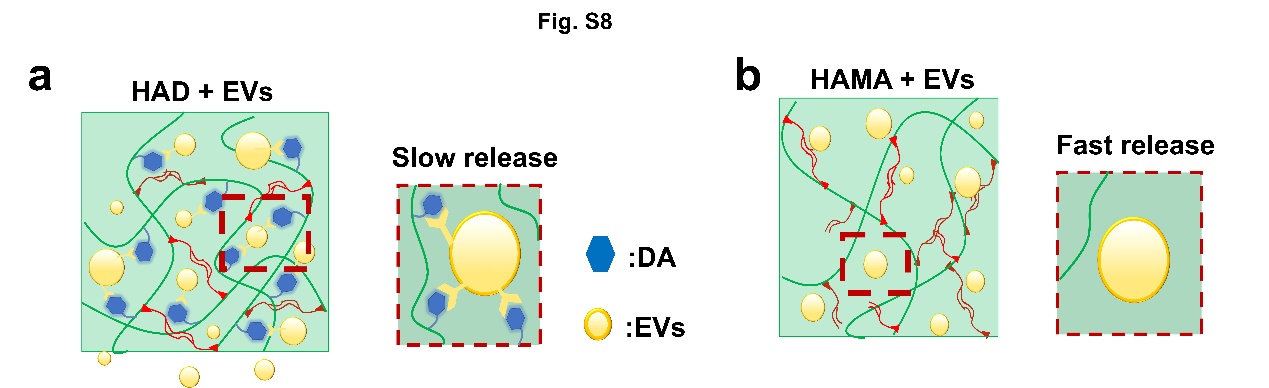


**cc**


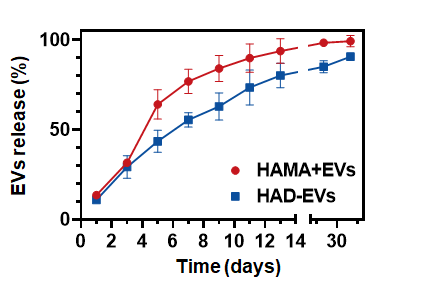


**Figure S11 a,b)** Schematic diagram of ADSC-EVs release from HAD+EVs and HAMA+EVs hydrogels. c) HAD can effectively bind EVs, and demonstrates a more sustained release performance.


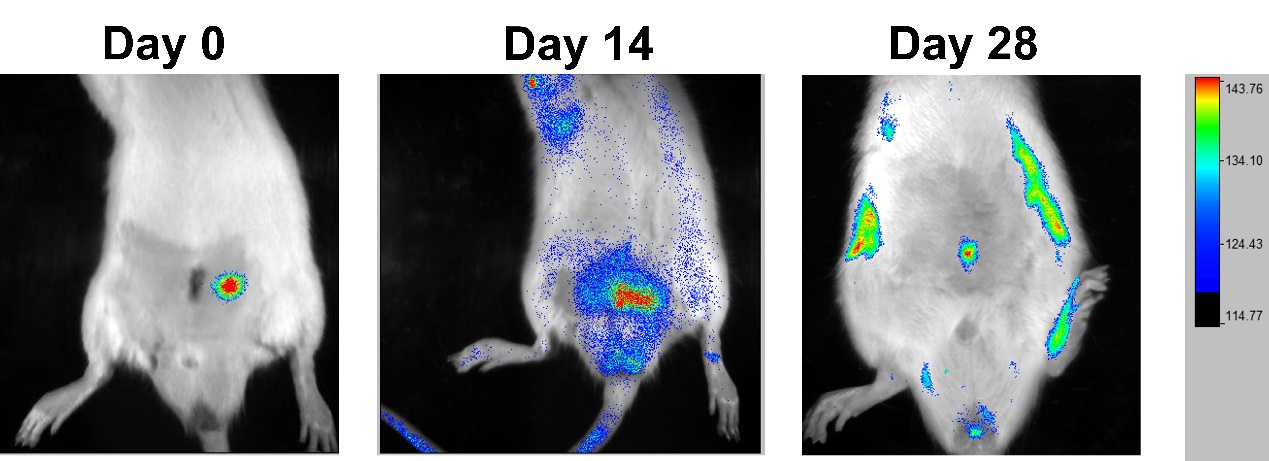


**Figure S12** In vivo live imaging of HAMA+EVs gel in rats.


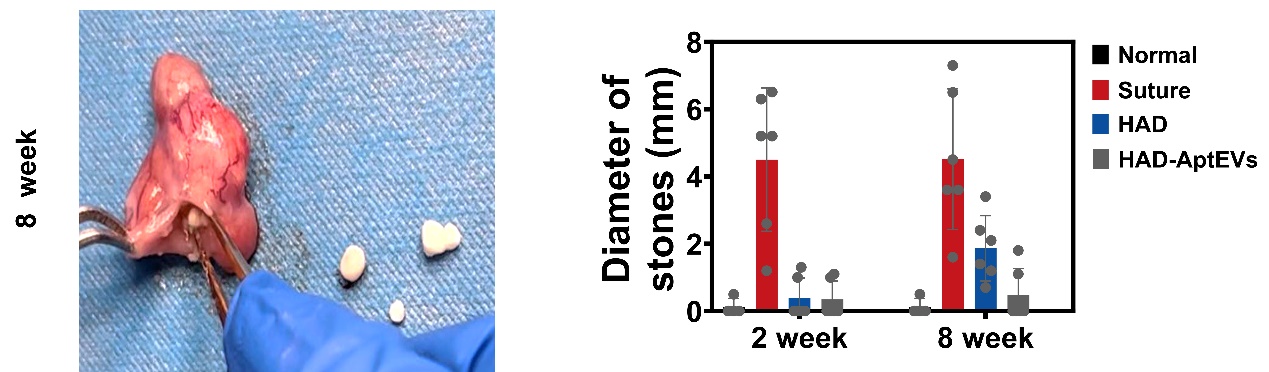


**Figure S13** Number of bladder stones in the Suture group.


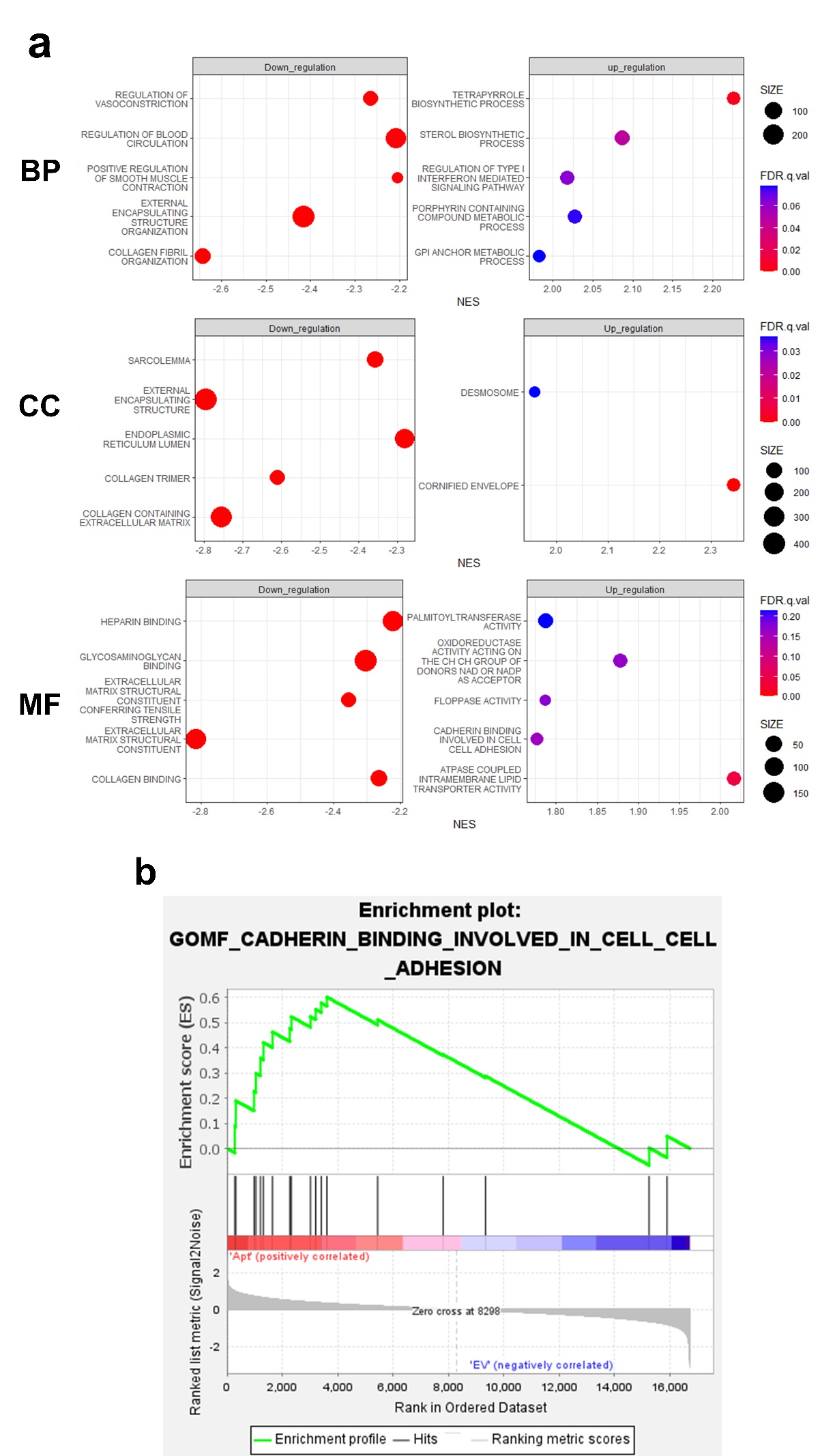


**Figure S14 GSEA Analysis of HAD-AptEVs vs. HAD Group.** a) GO enrichment analysis under GSEA for HAD-AptEVs vs. HAD group: Downregulation in fibrosis-related entries (such as collagen fibril organization, collagen-containing extracellular matrix); b) Enrichment plot for the gene set of cadherin binding in cell-cell adhesion in GSEA analysis for HAD-AptEVs vs. HAD, showing an upward trend.

**
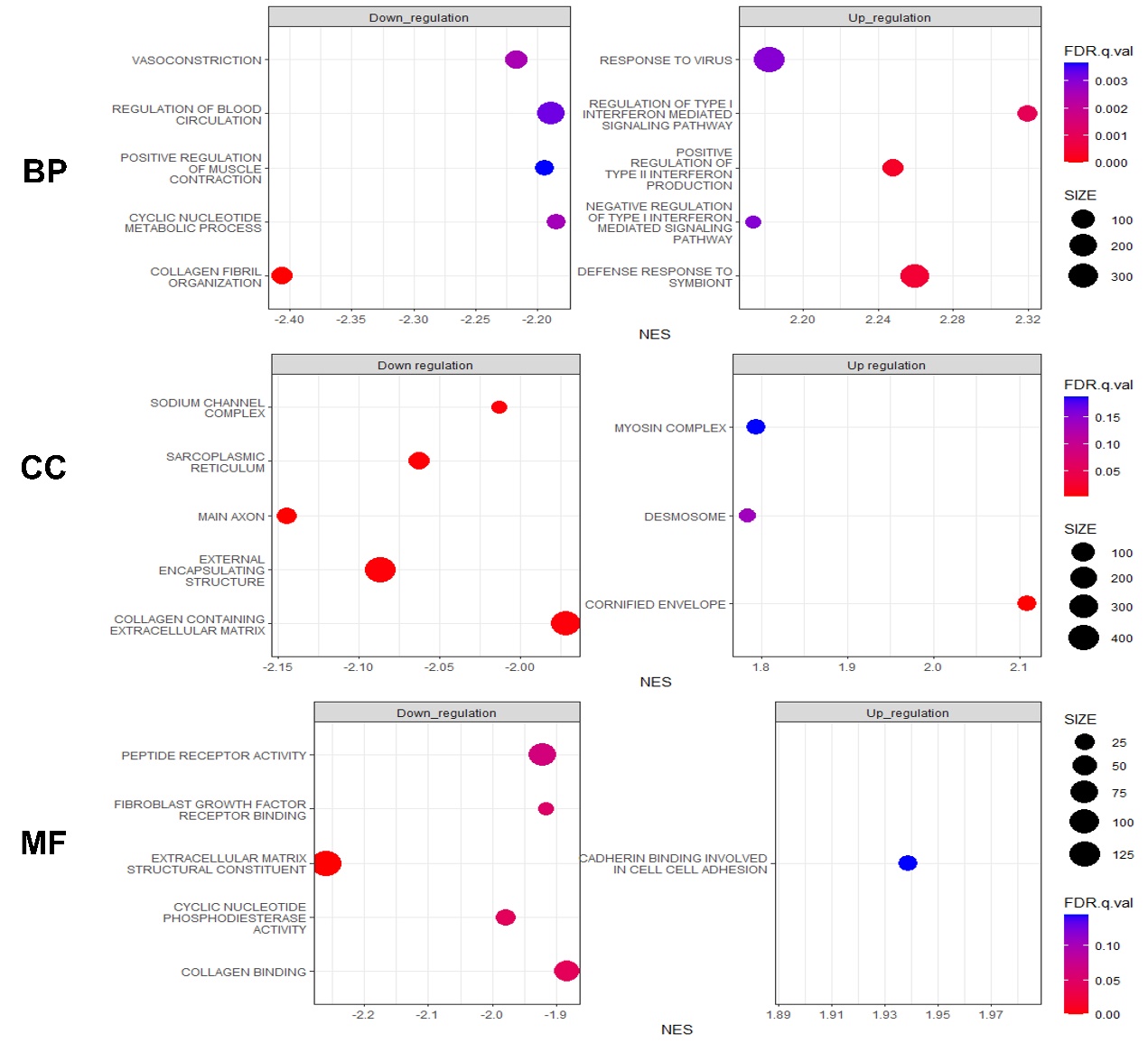
**

**Figure S15 GSEA Analysis of HAD-AptEVs vs. Suture Group:** GO enrichment analysis within GSEA for HAD-AptEVs vs. Suture group indicates a downregulation in fibrosis-related entries (such as collagen fibril organization, collagen-containing extracellular matrix, etc.) and an upregulation in entries related to cell adhesion (such as cadherin binding in cell-cell adhesion).


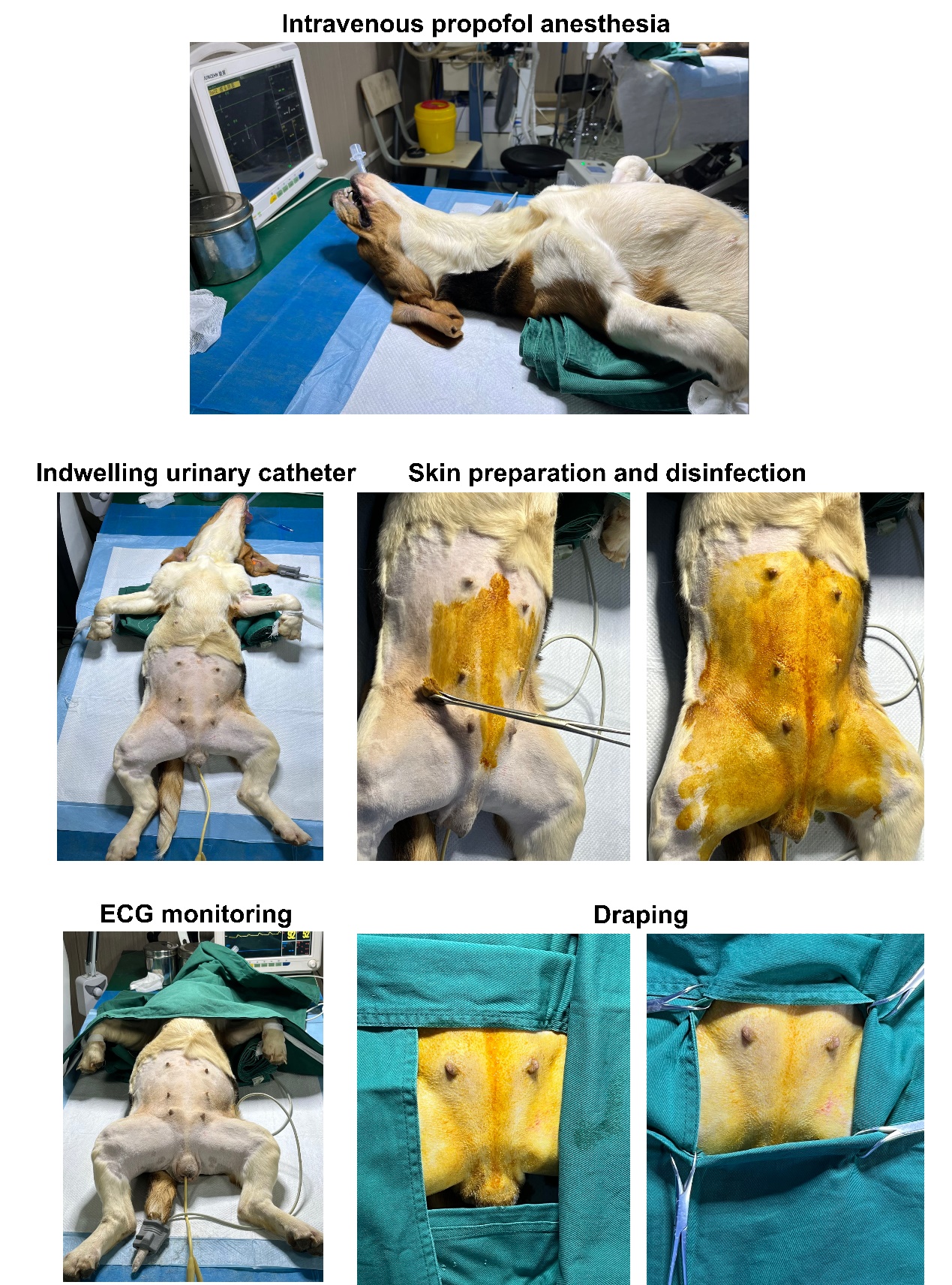


**Figure S16 Preoperative preparation of beagle dogs in each group:** skin preparation, intravenous propofol anesthesia, tracheal intubation, indwelling urinary catheter, ECG monitoring, disinfection, and draping.


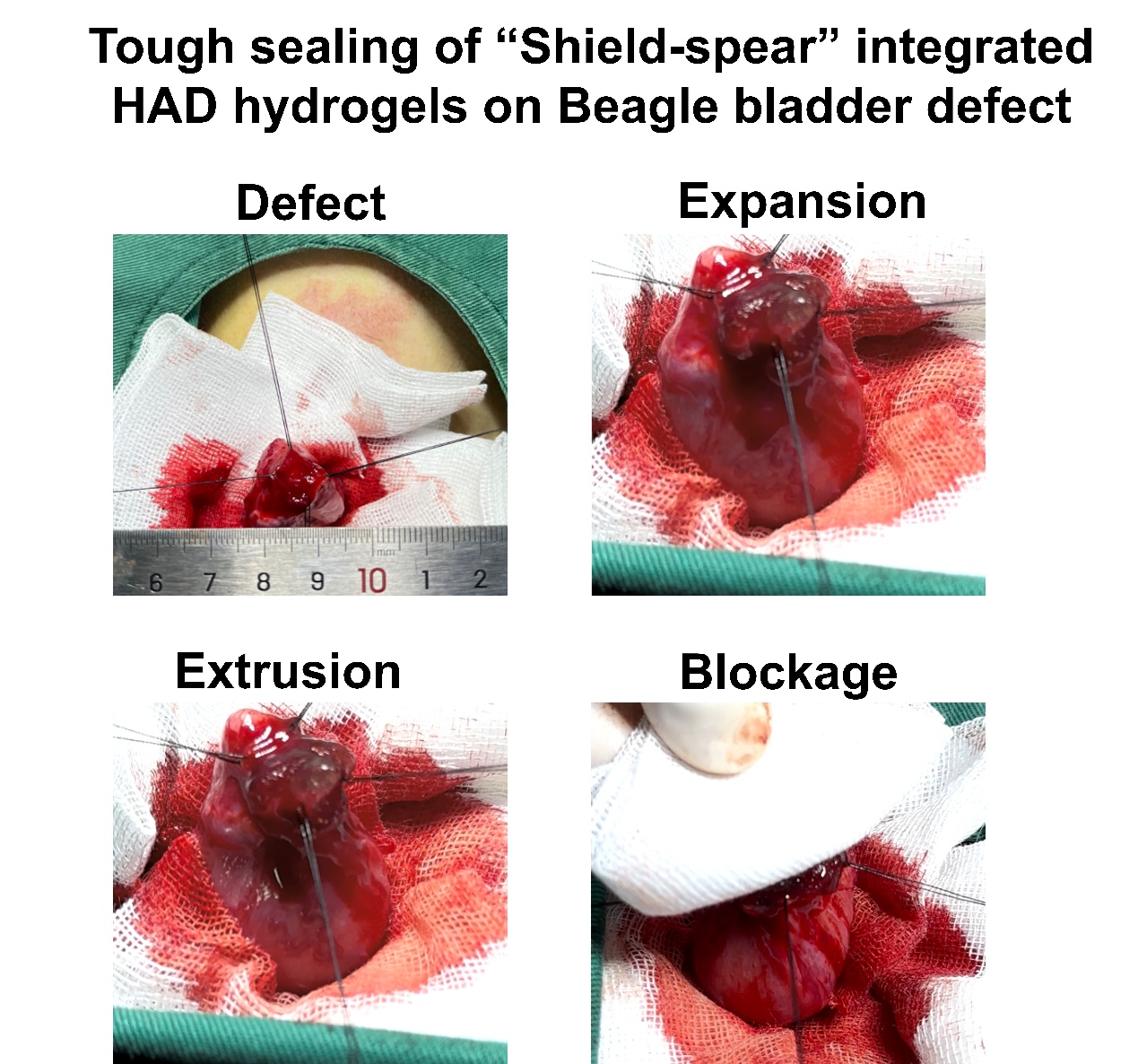


**Figure S17** HAD gel patch can achieve hemostasis and seal urinary fistula in beagle bladder defect model.


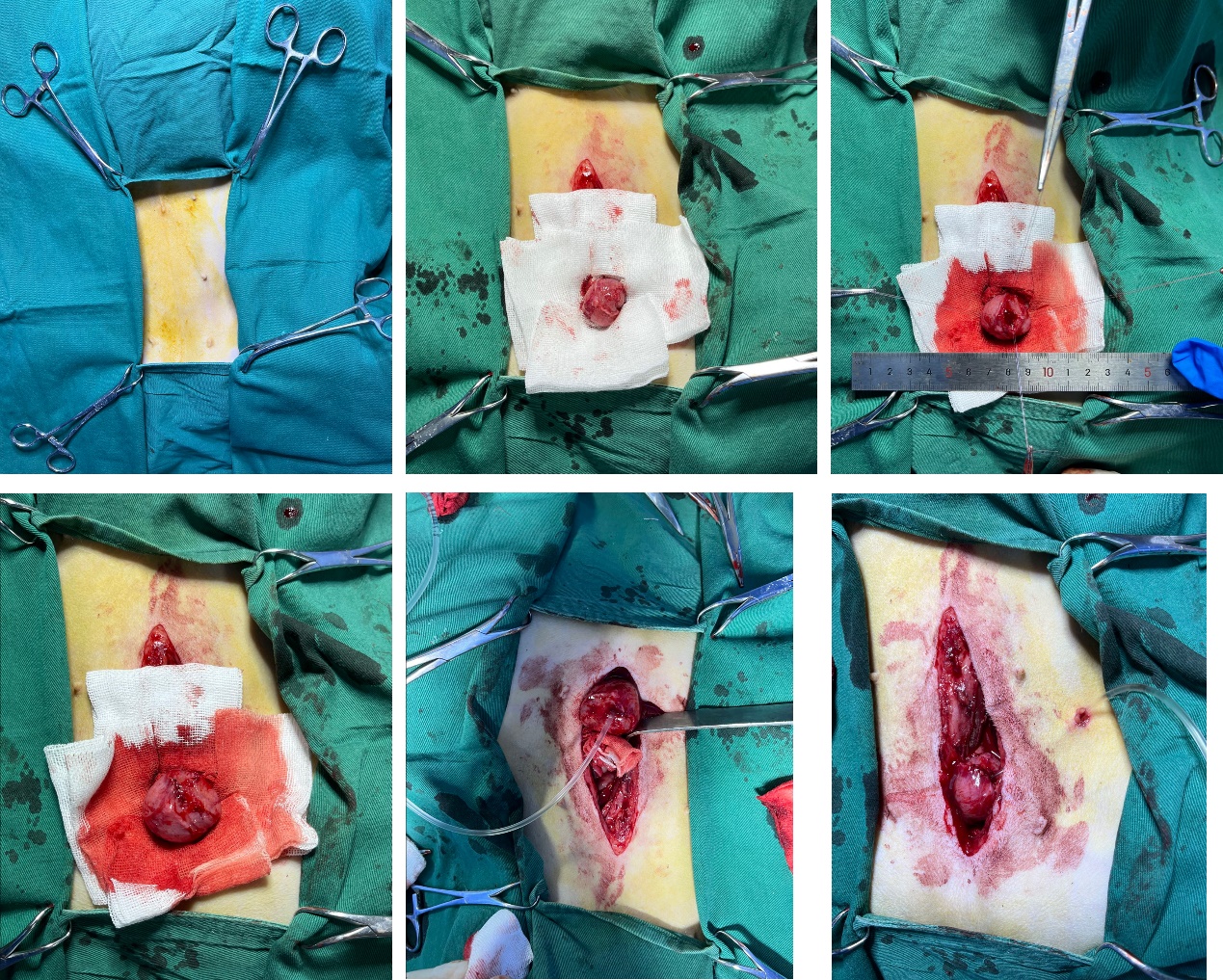


**Figure S18** The operation process in the Suture group.


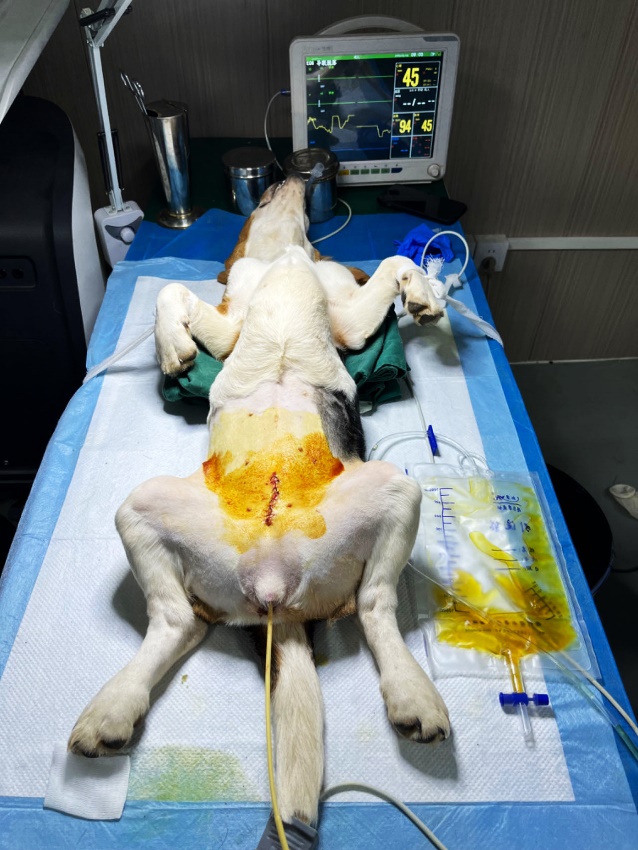


**Figure S19** ECG monitoring, anesthesia recovery and indwelling urinary catheter treatment for beagle dogs after surgery.


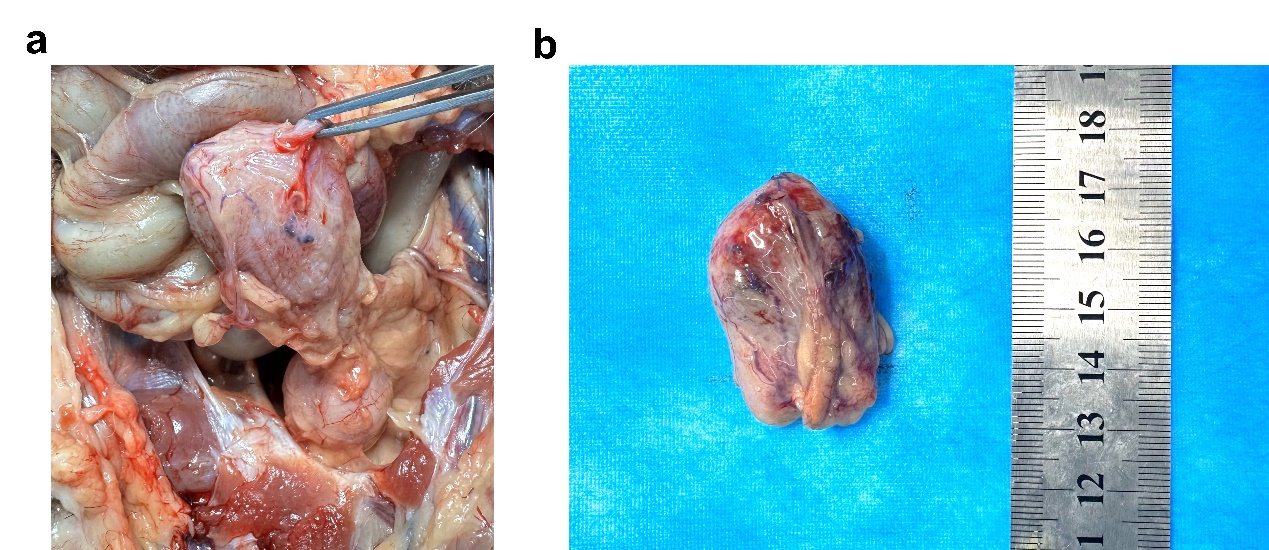


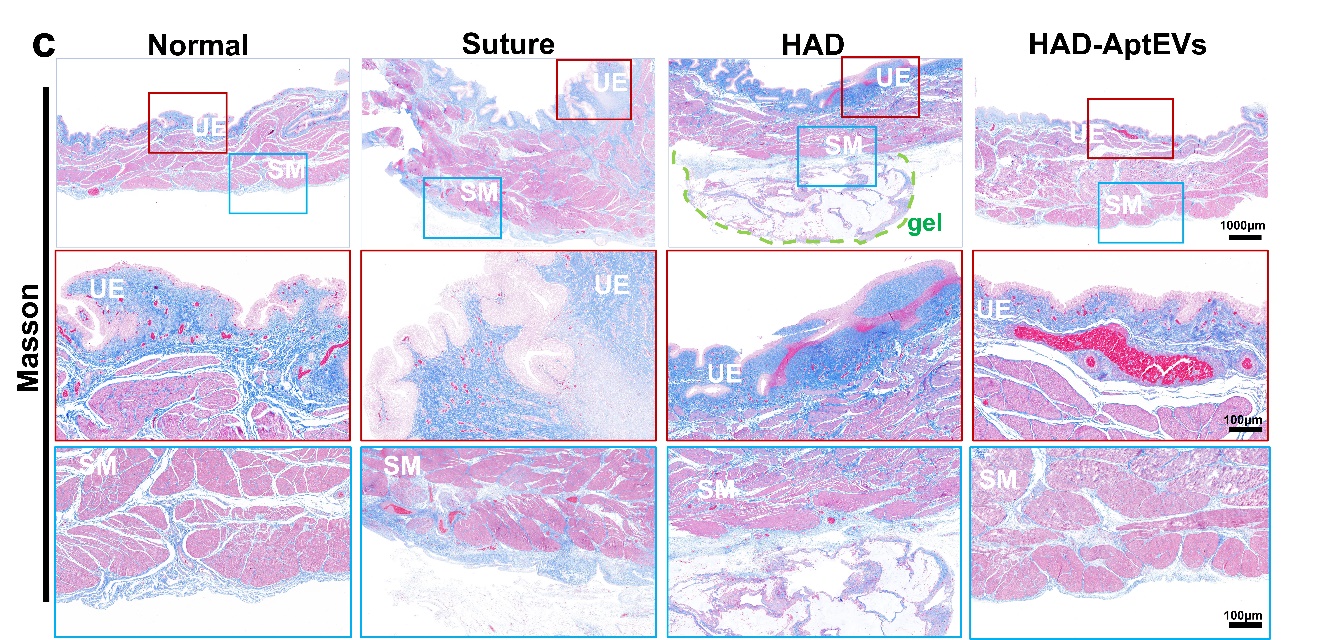
**Figure S20 a，b)** In the Suture group, beagles developed severe fibrous adhesions between the bladder wall and the intestinal tissues, as well as the abdominal wall, leading to the death of the sutured beagles 11 days postoperatively. c) Masson's trichrome-stained sections of tissue at the site of bladder defect healing in each group of beagles after 4 weeks (scale bars: 1000 μm for the upper row; 100 μm for the middle and lower row); UE represents urothelium; SM represents smooth muscle.


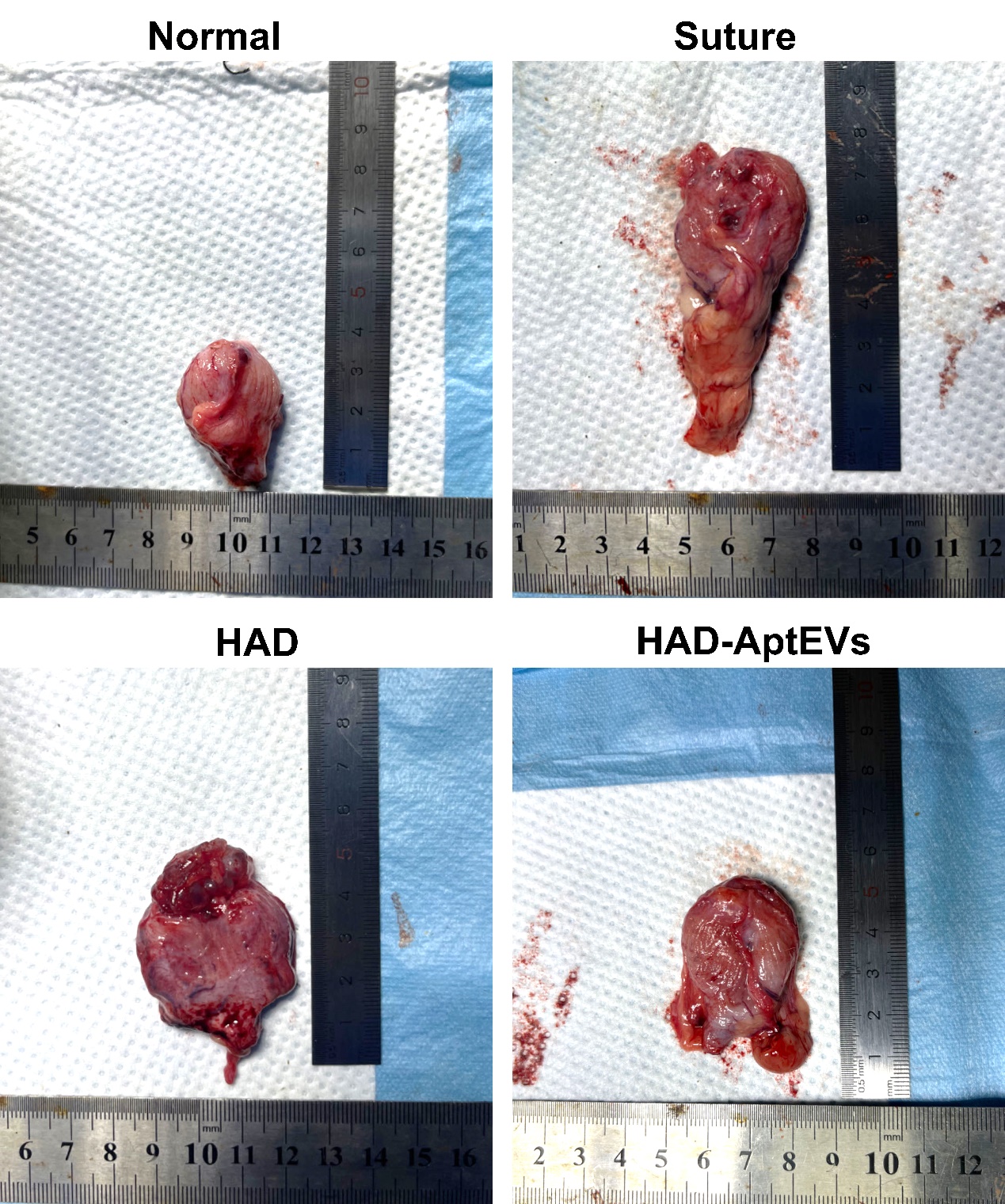


**Figure S21** Representative bladder samples from each group of beagle dogs at 1 month post-surgery.


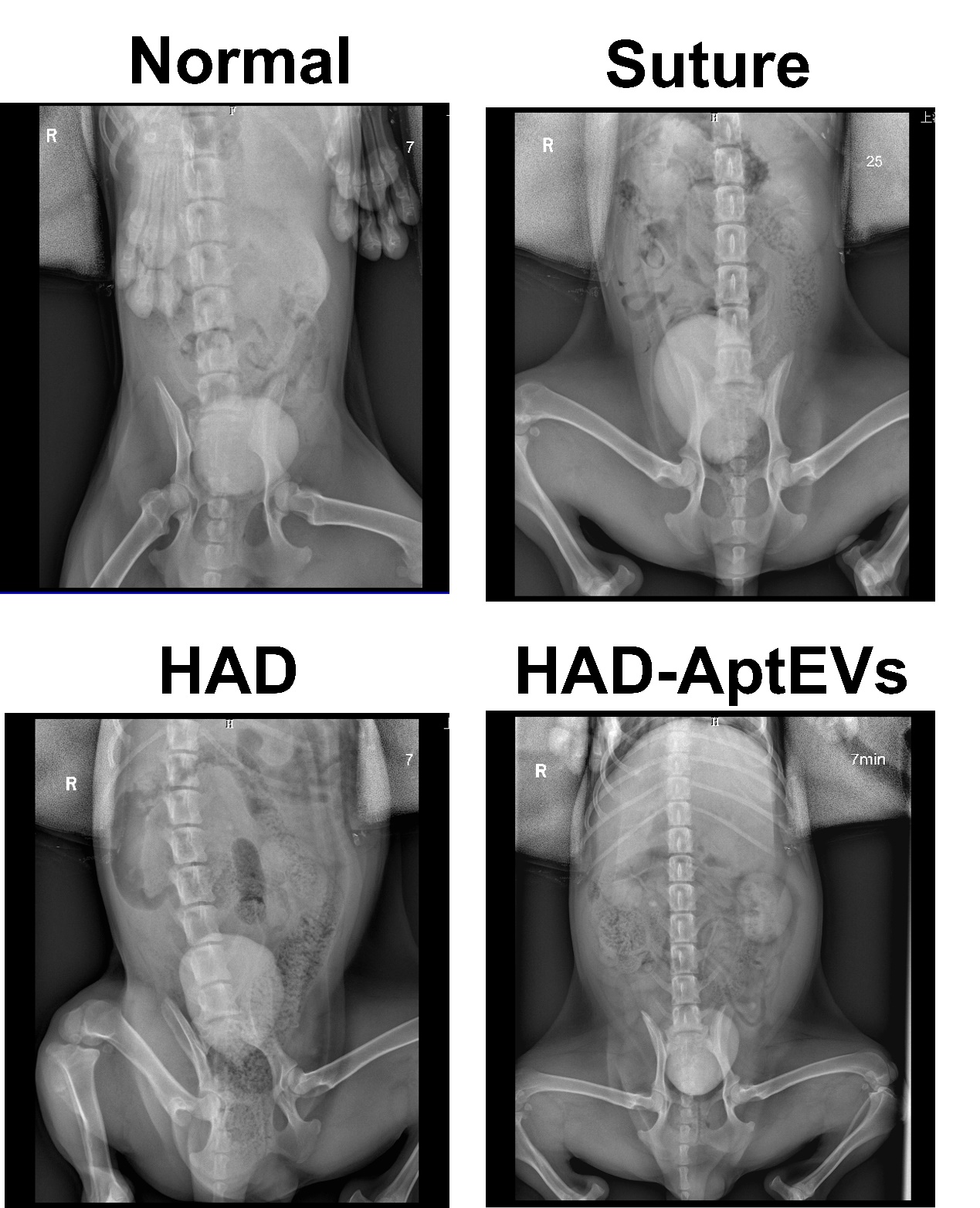


**Figure S22** The degree of bladder filling at the 10th minute of retrograde cystography in each group.


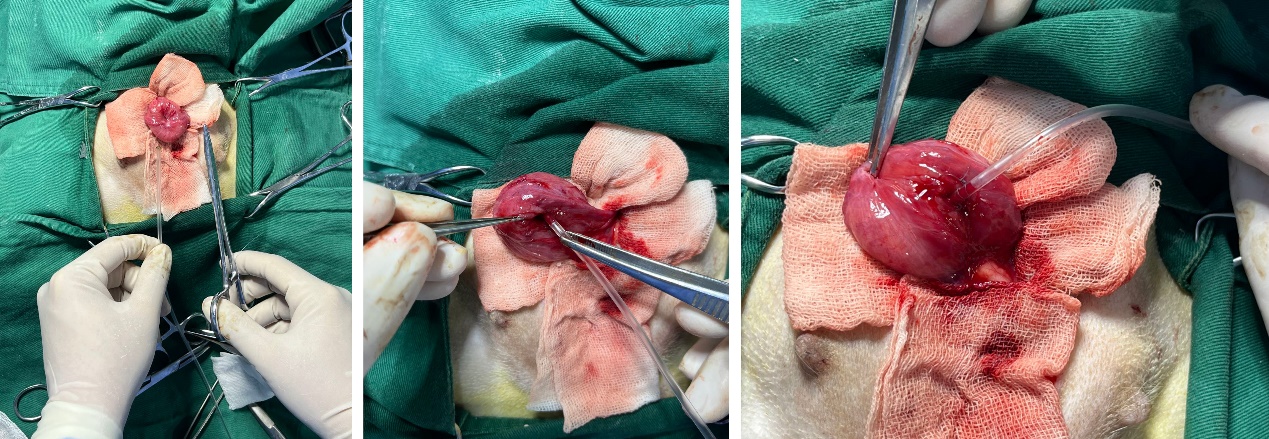


**Figure S23** Establishment of urinary dynamics channel in Beagle dogs: One end of a PE-50 catheter is implanted into the bladder of Beagle dogs using purse-string sutures, while the other end is passed through the subfascial layer to the abdominal wall skin and secured with a clip. It is then prepared for connection to the biosensor for pressure measurement.


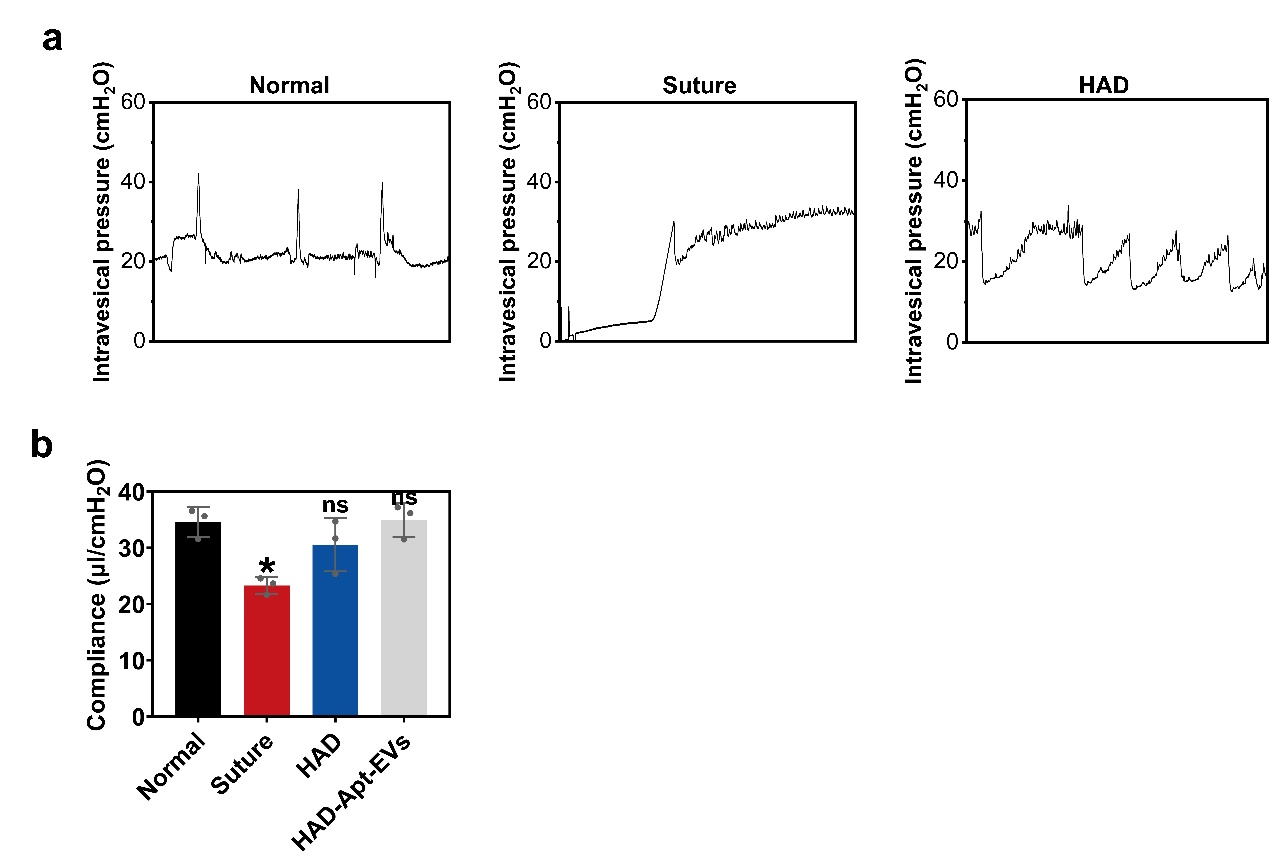


**Figure S24** **Urodynamic evaluation of beagle dogs in each group.** a) Real-time urodynamic curves in each group. b) Peak urodynamic values in each group. The statistical data are represented as the mean±SD (Compared with Normal group. *P<0.05, **P<0.01, ***P<0.001, ns= not significant).

**References**

1. Wu, X.*, et al.* Photocurable injectable Janus hydrogel with minimally invasive delivery for all-in-one treatment of gastric perforations and postoperative adhesions. *Theranostics* **13**, 5365-5385 (2023).

2. Wu, X.*, et al.* An Injectable Asymmetric‐Adhesive Hydrogel as a GATA6+ Cavity Macrophage Trap to Prevent the Formation of Postoperative Adhesions after Minimally Invasive Surgery. *Advanced Functional Materials* **32**(2021).

3. Xiao, D.*, et al.* MicroRNA-126 from stem cell extracellular vesicles encapsulated in a tri-layer hydrogel scaffold promotes bladder angiogenesis by activating CXCR4/SDF-1α pathway. *Chemical Engineering Journal* **425**, 131624 (2021).

4. Sun, W.*, et al.* A Novel DNA Aptamer Targeting S100P Induces Antitumor Effects in Colorectal Cancer Cells. *Nucleic Acid Therapeutics* **30**, 402-413 (2020).

5. Luo, Z.W.*, et al.* Aptamer-functionalized exosomes from bone marrow stromal cells target bone to promote bone regeneration. *Nanoscale* **11**, 20884-20892 (2019).

6. Xiao, D.*, et al.* Trilayer Three-Dimensional Hydrogel Composite Scaffold Containing Encapsulated Adipose-Derived Stem Cells Promotes Bladder Reconstruction via SDF-1α/CXCR4 Pathway. *ACS Appl Mater Interfaces* **9**, 38230-38241 (2017).

7. Hua, Y.*, et al.* Four-dimensional hydrogel dressing adaptable to the urethral microenvironment for scarless urethral reconstruction. *Nat Commun* **14**, 7632 (2023).

8. Feng, C.*, et al.* Electrospun Nanofibers with Core-Shell Structure for Treatment of Bladder Regeneration. *Tissue Eng Part A* **25**, 1289-1299 (2019).

9. Wang, Q.*, et al.* The morphological regeneration and functional restoration of bladder defects by a novel scaffold and adipose-derived stem cells in a rat augmentation model. *Stem Cell Res Ther* **8**, 149 (2017).

10. Xiao, D.*, et al.* Comparison of morphological and functional restoration between asymmetric bilayer chitosan and bladder acellular matrix graft for bladder augmentation in a rat model. *RSC Advances* **7**, 42579-42589 (2017).

11. Chen, S.H.*, et al.* Thermosensitive hydrogel carrying extracellular vesicles from adipose-derived stem cells promotes peripheral nerve regeneration after microsurgical repair. *APL Bioeng* **6**, 046103 (2022).

12. Zhou, Z.*, et al.* Adipose-derived stem-cell-implanted poly(ϵ-caprolactone)/chitosan scaffold improves bladder regeneration in a rat model. *Regen Med* **13**, 331-342 (2018).
